# Peer community health workers improve HIV testing and ART linkage among key populations in Zambia: retrospective observational results from the Z‐CHECK project, 2019–2020

**DOI:** 10.1002/jia2.26030

**Published:** 2022-11-01

**Authors:** Brianna R. Lindsay, Linah Mwango, Mona‐Gekanju Toeque, Siphiwe Lucy Malupande, Elizabeth Nkhuwa, Clement Nchimunya Moonga, Andrew Chilambe, Henry Sakala, Ina Kafunda, Pawel Olowski, Adebayo Olufunso, Jackson Okuku, Nzali Kancheya, Daliso Mumba, Lottie Hachaambwa, Robb Sheneberger, Natalia Blanco, Marie‐Claude Lavoie, Cassidy W. Claassen

**Affiliations:** ^1^ Center for International Health Education and Biosecurity MGIC‐an affiliate of the University of Maryland Baltimore Lusaka Zambia; ^2^ Center for International Health Education, and Biosecurity University of Maryland School of Medicine Baltimore Maryland USA; ^3^ Ciheb Zambia Lusaka Zambia; ^4^ Centre for Infectious Disease Research in Zambia (CIDRZ) Lusaka Zambia; ^5^ U.S. Centers for Disease Control and Prevention Zambia Lusaka Zambia; ^6^ National AIDS/TB/STI Council Zambia Lusaka Zambia

**Keywords:** people in prisons and enclosed settings, female sex workers, HIV testing yield, ART linkage, people who inject drugs, men who have sex with men

## Abstract

**Introduction:**

Zambia has made tremendous progress towards HIV epidemic control; however, gaps remain among key populations (KPs), such as female sex workers (FSWs), men who have sex with men (MSM), people who inject drugs (PWID) and people in prisons and enclosed settings due to cultural, social and legal barriers. The University of Maryland, Baltimore Zambia Community HIV Epidemic Control for Key Populations (Z‐CHECK) project aimed to improve HIV case‐finding, linkage and treatment adherence at the community level for KPs in Zambia. We describe Z‐CHECK strategies and examine HIV positivity yield and antiretroviral therapy (ART) linkage among KPs to inform ongoing programme improvement.

**Methods:**

Z‐CHECK recruited, trained and deployed peer community health workers (CHWs) for KP groups, with ongoing mentorship in community engagement. CHWs offered HIV testing in safe spaces and escorted newly HIV‐diagnosed clients for same‐day ART initiation. Z‐CHECK also reached out to KP community leaders and gatekeepers for KP mobilization and trained healthcare workers (HCWs) on KP services and sensitivity. We conducted a retrospective observational review of routinely collected aggregate data for KPs aged ≥15 years at high risk for HIV transmission across five districts in Zambia from January 2019 to December 2020.

**Results:**

Z‐CHECK provided HIV testing for 9211 KPs, of whom 2227 were HIV positive (positivity yield, 24%). Among these, 1901 (85%) were linked to ART; linkage for MSM, FSW, PWID and people in prisons and enclosed settings was 95%, 89%, 86% and 65%, respectively. Programme strategies that contributed to high positivity yield and linkage included the use of peer KP CHWs, social network testing strategies and opportunities for same‐day ART initiation. Challenges to programme implementation included stigma and discrimination among HCWs, as well as KP CHW attrition, which may be explained by high mobility.

**Conclusions:**

Peer CHWs were highly effective at reaching KP communities, identifying persons living with HIV and linking them to care. Engaging KP community gatekeepers resulted in high diffusion of health messages and increased access to health resources. The mobility of CHWs and HCWs is a challenge for programme implementation. Innovative interventions are needed to support PWID and people in prisons and enclosed settings.

## 1 INTRODUCTION

Zambia has a generalized HIV epidemic, with a prevalence of 11.1% among adults aged 15–49 [1] and an estimated 960,000 people living with HIV [2]. Southern Province has one of the highest prevalences at 12.5% [2]. While outreach efforts to the general population have been strengthened, traditional approaches often fail to serve marginalized groups. HIV disproportionately affects key populations (KPs) who face substantial psychosocial and structural obstacles to accessing health services. These barriers hamper further progress towards achieving 95‐95‐95 HIV epidemic control, including failures of diagnosis, linkage to treatment, retention and viral load suppression, particularly among KPs [3].

HIV transmission remains high among KPs, as defined by the Zambian National AIDS Strategic Framework 2017–2021 [4]. Sex work is thought to play a major role in HIV transmission in sub‐Saharan Africa and female sex workers (FSWs) are disproportionately affected by the HIV epidemic and have a higher risk of acquiring HIV [5, 6]. There are an estimated 160,000 FSWs in Zambia, with HIV prevalence estimates ranging from 29% to 56.4% [5, 7, 8]. Men, particularly those under 30, engaged in migrant work, men who have sex with men (MSM) and transgender (TG) persons are all at high risk of HIV transmission [7, 9, 10, 11, 12, 13]. MSM are estimated to number over 114,000, with high HIV prevalence at 21% [14]. As of 2021, there were an estimated 12,680 trans persons, of whom nearly 40% have not yet been reached with HIV testing [8, 15]. There are an estimated 26,840 people who inject drugs (PWID), with an HIV prevalence of 24% [8]. Over 22,000 people are incarcerated, with HIV prevalence estimates ranging from 14% to 27% [16, 17, 18]. Overall, KPs face high HIV prevalence resulting from complex determinants of transmission risk, including structural and individual factors [19, 20]. Stigma and discrimination in the healthcare setting are particularly challenging for MSM [9, 21, 22], while for FSWs, stable sexual partner concerns, lack of awareness of pre‐exposure prophylaxis (PrEP) and mistrust in PrEP are key barriers [23]. Meanwhile, interventions to decrease HIV transmission, such as voluntary medical male circumcision (VMMC) and PrEP, have low uptake among KPs [7].

Innovative strategies are required to find, test and link KPs to antiretroviral therapy (ART) and help sustain viral suppression. Community‐based differentiated service delivery (DSD) models leverage existing healthcare service delivery to decentralize and integrate HIV care and treatment with other HIV and non‐HIV services [24, 25] by task‐shifting routine tasks from clinical to non‐clinical staff [26]. Such community‐based approaches can reach underserved KPs who are not served by standard healthcare [27].

The Zambia Community HIV Epidemic Control for Key Populations (Z‐CHECK) project, implemented by the University of Maryland, Baltimore (UMB) and the Centre for Infectious Disease Research Zambia and in collaboration with the Ministry of Health (MOH), aimed to enhance HIV testing services (HTS), linkage to care and treatment, and adherence support at the community level for KPs. To identify KPs at risk of HIV infection, Z‐CHECK implemented the Community HIV Epidemic Control (CHEC) DSD model whereby community health workers (CHWs) reach KPs in their communities and offer HIV prevention and testing services. CHEC has previously been highly effective at reaching underserved populations in Zambia [28, 29]. This paper presents the Z‐CHECK approach to case finding and examines the HIV positivity yield and ART linkage rate with sub‐analyses by KP classification and district.

## 2 METHODS

### 2.1 Study design and setting

We used routinely collected aggregate data from January 2019 to December 2020 to conduct a retrospective observational study. We compare five districts (Mazabuka, Choma, Monze, Kaloma and Livingstone) in Southern Province where Z‐CHECK has been implemented since October 2016.

### 2.2 Study population

KPs served by the project include FSWs, MSM, TG, PWID and people in prisons and enclosed settings aged 15 and above. At the KP community members’ first encounter with the KP‐CHW, individuals were assessed for behavioural risk and assessed for classification as a KP. FSWs are defined as persons born female and involved in transactional sex in the past 6 months. MSM refers to males who engage in same‐sex sexual behaviour regardless of identified sexual orientation. PWID are identified as any person who has injected illegal drugs in the past 6 months. People in prisons and enclosed settings included were identified and served through one of five prisons in each of the five districts. Incarcerated persons receive health services within the correctional facility clinic or nearby government health facilities. We did not include TG persons in our analysis due to the low number of individuals reached.

### 2.3 Z‐CHECK intervention

Z‐CHECK offered HIV prevention and testing services to KPs using the UMB CHEC model, which ensures that HIV prevention and testing needs of KPs are achieved with minimal barriers. The programme was initiated through engaging KP civil society organizations to increase awareness, improve knowledge and build trust. Community members were then trained in KP sensitivity, safety and security services through either a 1‐day training for CHW or 2‐day training for healthcare workers (HCWs) (Supporting documents).

We recruited KP peer CHWs who were trained in community mobilization, psychosocial counselling, HTS and measures to enhance KP safety and security. These KP CHWs were then deployed to reach out to their communities with HIV prevention messages, screen for HIV risk and offer HTS. CHWs were supervised by a community liaison officer (CLO) who ensures that quality HIV services are offered and acts as a bridge between the CHWs to both the healthcare facilities and the community being served.

KP classification determined both the outreach and services individualized for each risk group. People in prisons and enclosed settings were identified at correctional facilities. For FSWs, MSM and TGs, interactions occurred in community safe spaces. FSWs were identified and recruited at hotspots, including brothels and lodges. PWID were engaged through shooting galleries or houses where injection use occurs.

HIV prevention, treatment and care modalities were initiated based on geographic location, resources and KP classification (Table 1). Services were offered through index testing outreach, community mobile hotspots or venues, or a social networking strategy (SNS) incentive‐based programme. The SNS programme identified “seeders” within KP networks through partnerships with civil society organizations and a KP screening form. Each seeder was then given coupons for distribution to their network members. Each subsequent KP who presented to the site with a coupon was offered psychosocial support, a risk assessment and HTS. Screening for high risk included questions about prior HIV testing history and results. KP who reported fever, cough, night sweats, weight loss, unprotected sex, sharing needles, soreness or unusual discharge from the genital regions or pregnancy were considered to be at substantial risk. For KPs who tested HIV positive, an additional screening tool was used to determine if they were newly diagnosed or previously identified as positive. Newly diagnosed persons were offered index testing, partner notification services and ART on site. All SNS sites offered same‐day ART initiation; if patients deferred treatment, they were referred to the nearest facility for ART. HIV‐negative KPs at substantial risk were offered prevention interventions, such as condoms and lubricants, PrEP, VMMC and cervical cancer screening. PWID were counselled on injection‐related harm reduction.

**Table 1:** Description of key population HIV prevention, treatment and care services, Southern Province, Zambia (January 2019–December 2020)

Intervention	KP	Location	Staffing	Frequency	Notes
Social network strategy	FSW MSM TG PWID	Facility	Nurse, CLO, CHW and recruiter	Weekly (3 days/week)	Mobilization is through peer‐to‐peer approach using SNS coupons.
KP Prev (condoms, lubricants, PEP and PrEP) HTS (testing and linkage to care, treatment and support) STI and TB screening (testing and linkage to treatment) ART adherence and retention support	FSW MSM TG PWID People in prisons and enclosed settings	Facility, hotspots, brothels and safe spaces	Nurses, CLOs and CHWs	Daily	Information, education and communication on HIV/AIDS done and how it can be prevented. HTS is done from anywhere provided that it is convenient and safe for both the RoC and the HCP. Screening is also prioritized during KP Prev and all STIs are managed using syndromic approach. This is key as we offer ART or PrEP adherence support to sustain efficacy of the medication.
Viral load testing	FSW MSM TG PWID People in prisons and enclosed settings	Health facility	Nurses	6 months after starting ART thereafter annually or as needed	This is our programme's efficiency measure for attaining epidemic control.
Harm reduction counselling	PWID	Facility, hotspots, brothels and safe spaces	Nurses, CLOs and CHWs	Every clinical appointment or as needed	

Abbreviations: ART, antiretroviral therapy; CHW, community health worker; CLO, community liaison officer; FSW, female sex worker; HCP, healthcare provider; KP, key population; KP Prev, individual and/or small group‐level HIV prevention interventions designed for the key populations; MSM, men who have sex with men; PEP, post‐exposure prophylaxis; PrEP, pre‐exposure prophylaxis; PWID, people who inject drugs; RoC, recipient of care; SNS, social network strategy; STI, sexual transmitted infection.

### 2.4 Outcomes and variables

Outcomes of interest included: (1) number of individuals tested for HIV; (2) positivity yield, defined as the number of individuals newly diagnosed as HIV positive divided by the total number of individuals who received HTS; and (3) linkage rate, defined as the number of individuals newly enrolled on ART divided by the number of individuals newly diagnosed as HIV positive.

HTS was disaggregated by modality: voluntary counselling and testing (VCT) and community testing, including community mobile testing and other community platforms. VCT includes standalone centres affiliated with a facility, including drop‐in centres and KP wellness centres. Community mobile covers testing in temporary locations and any other community‐based sites, including social network testing. To define outcomes and testing modalities, we used guidance from the PEPFAR Monitoring, Evaluation, and Reporting Indicator Reference Sheet (MER) version 2.4 [30].

### 2.5 Data sources and statistical analysis

Aggregated data were abstracted from routine MOH facility testing and linkage registers. At the community level, each CHW collected patient‐level information on community testing and elicitation forms and data were aggregated and reported at the affiliated facility level and combined by the district for analysis. These data were manually entered and stored in the District Health Information Software 2 data platform [31]. Personal identification codes were used to ensure that only unique individuals were reported. Descriptive statistics were calculated on outcomes of interest. The Mann–Kendall test was used to evaluate the trend across time depending on the variable distribution. All analyses were performed using SAS 9.4 (Carey, NC) and R‐4.0.3 [32].

### 2.6 Ethical approval

Ethical approval was granted by the institutional review boards (IRBs) at the ERES Converge Zambian IRB (Ref No. 2020‐Mar‐015), the National Health Research Authority (NHRA00029/04/2020) and the University of Maryland School of Medicine (HP‐00086064). This project was reviewed in accordance with CDC human research protection procedures and was determined to be non‐research, and informed consent was waived by the IRBs.

## 3 RESULTS

### 3.1 Programme implementation

Z‐CHECK engaged 50 community mobilizers (10 per district) to conduct demand generation for HIV prevention and testing services. Each mobilizer was assigned a KP type, equipped with information on prevention interventions and HTS, and was expected to reach at least 25 KPs monthly. Z‐CHECK engaged 25 KP‐CHWs to offer HTS in a KP‐friendly manner. All CHWs were assessed and certified as HIV testers by the MOH. Five CLOs were placed in each district to offer supervision to CHWs, and one technical lead to provide oversight support and develop strategies to reach KPs.

### 3.2 HIV testing and testing modalities

**Figure 1:**
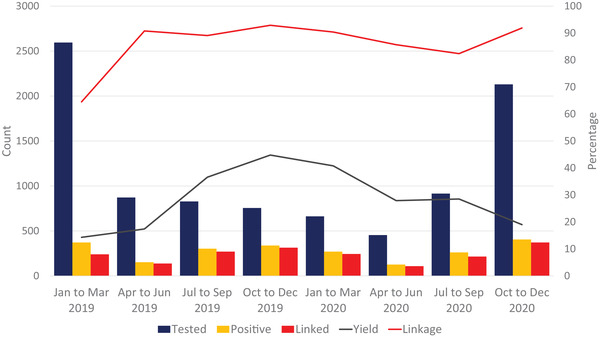
HIV testing yield and linkage to ART in key populations between January 2019 and December 2020 in the Southern Province of Zambia.

Between January 2019 and December 2020, a total of 9211 KP clients were tested for HIV with an overall positivity yield of 24.2% (Table 2). From January to March 2019 and October to December 2020, more tests were conducted than in any of the other quarterly periods, 2595 and 2130, respectively. In the other quarters, HTS remained relatively consistent, ranging from 454 in April–June 2020 to 915 in July–September 2020 (Figure 1). A median of 850 (IQR 732–1218) individuals was tested per quarter. Mazabuka district had the lowest positive yield at 18.0% and Monze had the highest at 38.7% (Table 2).

**Table 2:** HIV testing and newly diagnosed of KPs sub‐groups disaggregated by districts and periods between January 2019 and December 2020 in the Southern Province of Zambia

	People in prisons and enclosed settings			PWID			MSM			FSW			Total		
	Tested	HIV positive	Yield	Tested	HIV positive	Yield	Tested	HIV positive	Yield	Tested	HIV positive	Yield	Tested	HIV positive	Yield
	*N*	*n*	(%)	*N*	*n*	(%)	*N*	*n*	(%)	*N*	*n*	(%)	*N*	*n*	(%)
**District**															
Choma	120	50	41.7	0	0	–	15	4	26.7	830	272	32.8	965	326	33.8
Kalomo	94	11	11.7	0	0	–	0	0	–	162	44	27.2	256	55	21.5
Livingstone	2215	269	12.1	358	83	23.2	1292	277	21.4	2718	870	32.0	6583	1499	22.8
Mazabuka	406	40	9.9	0	0	–	6	2	33.3	540	129	23.9	952	171	18.0
Monze	72	18	25.0	3	2	66.7	1	1	100.0	379	155	40.9	455	176	38.7
**Periods**															
Jan–Mar 2019	1382	159	11.5	51	13	25.5	297	31	10.4	865	169	19.5	2595	372	14.3
Apr–Jun 2019	331	41	12.4	27	3	11.1	200	17	8.5	314	91	29.0	872	152	17.4
Jul–Sep 2019	272	37	13.6	46	9	19.6	145	71	49.0	365	186	51.0	828	303	36.6
Oct–Dec 2019	122	32	26.2	72	32	44.4	117	53	45.3	444	221	49.8	755	338	44.8
Jan–Mar 2020	179	41	22.9	28	3	10.7	142	55	38.7	313	171	54.6	662	270	40.8
Apr–Jun 2020	132	18	13.6	14	2	14.3	91	17	18.7	217	89	41.0	454	126	27.8
Jul–Sep 2020	125	19	15.2	28	6	21.4	118	14	11.9	644	222	34.5	915	261	28.5
Oct–Dec 2020	364	41	11.3	95	17	17.9	204	26	12.7	1467	321	21.9	2130	405	19.0
**Totals**	2907	388	13.3	361	85	23.5	1314	284	21.6	4629	1470	31.8	9211	2227	24.2
**Trend**	*p* = 0.80, *Z* = 0.25, tau = 0.11			*p* = 0.90, *Z* = –0.12, tau = –0.07			*p* = 0.90, *Z* = –0.12, tau = –0.07			*p* = 1.0, *Z* = 0.0, tau = 0.0			*p* = 0.71, *Z* = 0.37, tau = 0.14		

Abbreviations: FSW, female sex worker; MSM, men who have sex with men; PWID, people who inject drugs.

Community mobile and other community testing accounted for most of the HTS overall across the 24 months, 5834 (63.3%). HTS modality was not disaggregated by KP classification; however, community testing led to an overall higher yield, 29.5%. In 2019, aside from the first quarter (January–March 2019), most of the testing was done via community and a relatively modest contribution from VCT in 2020 (Table 3). The highest yield was demonstrated in Choma (28.0% and 37.5%) and Monze (20.6% and 49.0%) for VCT and community testing, respectively.

**Table 3:** Key population HIV testing and newly diagnosed HIV cases by testing modality between January 2019 and December 2020 in the Southern Province of Zambia

	Voluntary counselling and testing			Community testing (community mobile and other)^a^			Total		
	Tested	HIV positive	Yield	Tested	HIV positive	Yield	Tested	HIV positive	Yield
	*N*	*n*	(%)	*N*	*n*	(%)	*N*	*n*	(%)
**District**									
Choma	378	106	28.0	587	220	37.5	965	326	33.8
Kalomo	91	10	11.0	165	45	27.3	256	55	21.5
Livingstone	2666	345	12.9	3917	1154	29.5	6583	1499	22.8
Mazabuka	77	13	16.9	875	158	18.1	952	171	18.0
Monze	165	34	20.6	290	142	49.0	455	176	38.7
**Periods**									
Jan–Mar 2019	2595	372	14.3	0	0	.	2595	372	14.3
Apr–Jun 2019	0	0	0.0	872	152	17.4	872	152	17.4
Jul–Sep 2019	0	0	0.0	828	303	36.6	828	303	36.6
Oct–Dec 2019	9	3	33.3	746	335	44.9	755	338	44.8
Jan–Mar 2020	179	41	22.9	483	229	47.4	662	270	40.8
Apr–Jun 2020	132	18	13.6	322	108	33.5	454	126	27.8
Jul–Sep 2020	125	19	15.2	790	242	30.6	915	261	28.5
Oct–Dec 2020	337	55	16.3	1793	350	19.5	2130	405	19.0
**Totals**	3377	508	15.0	5834	1719	29.5	9211	2227	24.2

^a^Testing in mobile ad hoc or temporary testing locations, such as community centres, schools and workplaces, and includes testing in mobile unit, such as tents and vans.

### 3.3 Testing and treatment cascade by KP type

From January 2019 to December 2020, 2907 people in prisons and enclosed settings were tested for HIV, with a positivity yield of 13.3%. The positivity yield in persons incarcerated varied by district, ranging from 9.9% in Mazabuka to 41.7% in Choma (Table 2). Of the KP types presented, the linkage was lowest in the inmate population (64.7%), driven by a low proportion in the first quarter of 2019 (Table 4).

**Table 4:** HIV linkage to ART by key population classification and district between January 2019 and December 2020 in the Southern Province of Zambia

	People in prisons and enclosed settings			PWID			MSM			FSW			Total		
	HIV positive	Linked	Linkage	HIV positive	Linked	Linkage	HIV positive	Linked	Linkage	HIV positive	Linked	Linkage	HIV positive	Linked	Linkage
	*n*	*N*	(%)	*n*	*N*	(%)	*n*	*N*	(%)	*n*	*N*	(%)	*n*	*N*	(%)
**Districts**															
Choma District	50	43	86.0	0	0	–	4	4	100.0	272	199	73.2	326	246	75.5
Kalomo District	11	7	63.6	0	0	–	0	0		44	35	79.5	55	42	76.4
Livingstone District	269	153	56.9	83	71	85.5	277	264	95.3	870	813	93.4	1499	1301	86.8
Mazabuka District	40	34	85.0	0	0	–	2	2	100.0	129	120	93.0	171	156	91.2
Monze District	18	14	77.8	2	2	100.0	1	0	0.0	155	140	90.3	176	156	88.6
**Periods**															
Jan–Mar 2019	159	50	31.4	13	11	84.6	31	29	93.5	169	150	88.8	372	240	64.5
Apr–Jun 2019	41	35	85.4	3	3	100.0	17	16	94.1	91	84	92.3	152	138	90.8
Jul–Sep 2019	37	32	86.5	9	6	66.7	71	63	88.7	186	169	90.9	303	270	89.1
Oct–Dec 2019	32	29	90.6	32	28	87.5	53	53	100.0	221	204	92.3	338	314	92.9
Jan–Mar 2020	41	35	85.4	3	3	100.0	55	55	100.0	171	151	88.3	270	244	90.4
Apr–Jun 2020	18	14	77.8	2	2	100.0	17	17	100.0	89	75	84.3	126	108	85.7
Jul–Sep 2020	19	16	84.2	6	4	66.7	14	14	100.0	222	181	81.5	261	215	82.4
Oct–Dec 2020	41	40	97.6	17	16	94.1	26	23	88.5	321	293	91.3	405	372	91.9
**Total**	388	251	64.7	85	73	85.9	284	270	95.1	1470	1307	88.9	2227	1901	85.4
**Trend**	*p* = 0.45, *Z* = 0.75, tau = 0.25			*p* = 0.89, *Z* = 0.13, tau = 0.08			*p* = 0.69, *Z* = 0.40, tau = 0.16			*p* = 0.32, *Z* = –0.99, tau = –0.33			*p* = 0.90, *Z* = 0.12, tau = 0.07		

Abbreviations: FSW, female sex worker; MSM, men who have sex with men; PWID, people who inject drugs.

A total of 361 PWID were tested with an overall positivity yield of 23.5%. The majority (99%) of PWID reached with HTS occurred in Livingstone (Table 2). ART linkage was 85.9% overall in this KP group, with 100% linkage observed in three of the eight quarters examined (Table 4).

FSWs represented a large proportion of the KPs reached during the period under evaluation. A total of 4629 FSWs were tested for HIV (Table 2). FSWs also accounted for 68.8% (1307/1901) of all KPs linked to treatment (Table 4), where linkage ranged from 73.2% to 93.4% across districts and 81.5% to 92.3% over time, peaking in October–December 2019.

A total of 1314 MSM were HIV tested and new positives were identified from 21.6%. HTS yield varied from 8.5% in April–June of 2019 to 49.0% in the following quarter. Linkage was highest overall among MSM (95.1%) compared to other KP types. From October 2019 to September 2020, a 15‐month period, MSM linkage was 100%.

The distribution of KP types in Southern Province varied. MSM testing candidates and PWID were found primarily in Livingstone, while FSW testing occurred across all districts (Table 2). A higher overall positivity yield was observed for FSW than MSM (31.8% vs. 21.6%). KP linkage to ART was relatively consistent over time but varied slightly regarding KP classification and district. Linkage overall ranged from an initial 64.5% in January–March 2019 to a peak of 92.9% between October and December 2020 and a slight decrease to 82.4% in July–September 2020. There was no statistically significant association of linkage over time overall, nor for any of the KP groups individually.

There were fluctuations in the number of individuals tested and yield around the initial COVID‐19 wave; 662 provided HTS and 40.8% yield overall in January–March 2020 compared to 454 tested and 27.9% in April–June 2020 (*p*‐trend = 0.71).

## 4 DISCUSSION

HIV testing and treatment programmes among KPs have been substantially strengthened in recent years. We report on one of the first implementations of KP HIV case‐finding in Zambia using a community‐based DSD model, KP CHWs and targeted testing strategies. Among KP programmes, targets and prioritization of populations (as set by MOH and PEPFAR) change over time as demonstrated by fluctuations in numbers and yield of HIV test results over these 2 years. Reporting from different testing modalities also varied over time; however, most testing was conducted in the community. This KP programme aimed to meet clients where they were most accessible and felt most comfortable. Given the vulnerability of these populations, community service delivery may be an optimal strategy to reach KPs with interventions. Other KP programmes have also reported a high linkage to ART using DSD to reach diverse populations [33]. This highlights the efficacy of the DSD model of community initiation and demonstrates the ability of CHWs and HCWs to facilitate treatment when individuals who test positive are identified.

### 4.1 Outcomes by KP type

From January to March 2019, HTS was initiated by Z‐CHECK in correctional facilities, resulting in a surge in testing overall during this period but with low linkage to treatment. After this initial quarter, interventions in correctional facilities were refocused through retraining and educational programmes and linkage to treatment improved in the last quarter of 2020. Among the four KP types, people in prisons and enclosed settings had the lowest HTS yield and lowest linkage to care. This can be attributed to high inmate turnover and structural barriers bridging prison to community health facilities, particularly inadequate HCW staffing to support same‐day ART initiation [34]. In Eastern and Southern Africa, HIV prevalence among detainees has been reported at 15.6%, while in Zambia, prevalence is estimated at between 23% and 27% [18, 35, 36].

A 2015 literature review reported no prior literature documenting the full care cascade of PWID in Africa [37]. However, a survey in Mozambique reported that among HIV‐infected PWID participants, 80% had previously been tested for HIV, 63% were aware of their HIV status and 49% (*n* = 100) reported being linked to care for their HIV infection [38]. In Kenyan PWID on methadone, ART linkage was reported to be 96% [39]. Documented linkage was relatively high (77–79%) among Hispanic PWID populations in the United States; however, the linkage was only 32% among PWID in India [37]. While PWID constituted the lowest proportion of individuals reached overall in our population, the linkage was high.

Knowledge of the availability of HIV testing among the MSM community is high, and evidence suggests that MSM in sub‐Saharan Africa are responsive to outreach and engagement in risk reduction activities [40]. In Zambia, along with many African countries, same‐sex sexual relationships are criminalized, creating a significant barrier to reaching these populations. The majority of MSM reached with the prevention, testing and ART services by Z‐CHECK were found in the Livingstone district with high yield and linkage to treatment. In contrast, a recent survey identified significant gaps in the HIV treatment cascade of KPs in Mozambique, and only 6% of HIV‐positive MSM reported being linked to care [38].

FSWs represent a large proportion of the KP communities reached by Z‐CHECK. Driven by programmatic priorities, a high number of HIV tests were conducted with FSWs from January to March 2019 and then again from October to December 2020. Given the long history of serving this population, the Z‐CHECK CHWs and HCWs have deep experience in reaching FSWs, which likely resulted in the remarkably high testing yield and linkage to treatment. Similarly, among FSWs in Cote d'Ivoire, enhanced peer outreach strategies using a similar SNS approach demonstrated higher case‐finding, linkage to treatment and treatment initiation compared to non‐social network‐based approaches [41]. Additional evidence suggests that venues associated with formal sex work could be locations of potential missed opportunities for testing high‐risk individuals [41, 42].

### 4.2 Impact of COVID‐19

The first case of COVID‐19 in Zambia was reported on 18 March 2020. At the pandemic's start, MOH established a partial lockdown across the country, which was lifted in May 2020. While the overall number of HIV tests conducted and yield varied slightly over time, linkage to care was consistent. The focus on reaching targets was renewed with the initiation of a new programme year, and a distinct focus on KPs was demonstrated by an increase in the number of clients tested and initiated on treatment. The end of 2020 in Zambia represented a key time for the delivery of prevention and treatment services because of a relatively low prevalence of COVID‐19 in the population and prioritization of resources given anticipated interruptions in activities due to upcoming election activity in 2021. All programmatic staff were trained in COVID‐19 mitigation procedures and adapted to periods of higher incidence by focusing efforts on community service delivery as much as possible. Our results show a higher yield in early 2020 compared to later in the year; however, there was no statistically significant trend over time. Reports of COVID‐19 impact on HIV programmes in Zimbabwe and Malawi indicated decreased numbers of tests conducted overall but only slight changes in yield [43].

### 4.3 Limitations/challenges

Reaching KPs is complicated by their legal status and general vulnerabilities within their communities. While the peer approach has successfully identified KPs with high HIV testing yield and linkage to treatment, stigma and discrimination can still have a demonstrative impact on engagement in care. Fear of stigma plays a major role in KPs’ hesitancy to access healthcare services, as they fear that they will be condemned by HCWs [44]. Retaining and recruiting experienced CHWs can be a barrier to programme implementation, and these difficulties may be explained by high mobility, stigma and community discrimination [45]. In our programme, CHW mobility tended to be seasonal as they would move in search of more money or for fear that it would be known to their community that they were KPs.

The strategies and data presented here represent a programme built around specific objectives of meeting KPs’ needs. Prioritizing KP individuals and resources required can be a barrier to implementation in non‐KP‐focused interventions. The use of a retrospective non‐randomized observational study design can introduce bias due to the availability of existing data and the lack of a randomly assigned control group. While we were able to describe the linkage to treatment following HIV testing, we did not have longitudinal, patient‐level data on viral load follow‐up to determine rates or time to suppression. Retention in care and viral suppression are critical components of effective treatment and transmission prevention, which were not measured in our study. We also acknowledge the limitation of a convenience sample; individuals reached via Z‐CHECK may be more willing to engage than the general population, representing selection bias. However, data under our programmatic scope were closely monitored for completeness and consistency by a monitoring and evaluation team throughout implementation. While each country in sub‐Saharan Africa (SSA) faces its own unique cultural and epidemiologic contexts, many face similar challenges in providing HIV service delivery to KPs. We believe that the Z‐CHECK approach presents a best practice of using peer CHWs and social network testing to find and link KPs to HIV service delivery, which may be applicable in other countries in the region.

## 5 CONCLUSIONS

The Z‐CHECK programme successfully used a peer CHW DSD model to reach KPs in Zambia. Peer networks played an integral role in engaging KPs and improving health services. We found the highest HIV positivity among FSWs, highlighting risk in this group, while ART linkage was lowest among inmates, reflecting the transiency of this group with subsequent challenges to treatment. We recommend scaling up KP‐friendly services through training more HCWs in KP sensitivity, safety and security services as well as collaboration with local governments to implement HIV prevention programmes targeting PWID. Overall, in this KP programme, HIV testing, identifying positive clients and linkage to treatment were high, and peer‐centred strategies effectively delivered HIV prevention and treatment services.

## COMPETING INTERESTS

The authors declare that they have no competing interests.

## AUTHORS’ CONTRIBUTIONS

All authors contributed to writing, reviewing and editing the manuscript. All authors have read and approved the final manuscript.

## FUNDING

The Z‐CHECK project and this publication have been supported by the President's Emergency Plan for AIDS Relief (PEPFAR) through the Centers for Disease Control and Prevention (CDC) under the terms of U2GGH001913.

## DISCLAIMER

The views expressed in the manuscript do not necessarily represent the views of the CDC. The findings and conclusions in this manuscript are those of the authors and do not necessarily represent the official position of the funding agencies.

## Supporting information




Psychosocial Counselling

Participant Workbook

## Data Availability

The protocol for the analysis of Z‐CHECK programme data is available upon reasonable request to the corresponding author. Aggregate data used in these analyses are accessible in PEPFAR Panorama Spotlight Monitoring, Evaluation, and Reporting (MER) Datasets.
