# Supplementary material for: Peer community health workers improve HIV testing and ART linkage among key populations in Zambia: retrospective observational results from the Z‐CHECK project, 2019–2020
Source: J Int AIDS Soc. 2022 Nov 1;25(11):e26030. doi: 10.1002/jia2.26030 (PMC9624072; doi:10.1002/jia2.26030)
Supplement: Supplementary file 1 — Psychosocial Counselling [file JIA2-25-e26030-s002.docx]

| 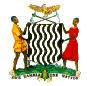  MINISTRY OF HEALTH  PSYCHOSOCIAL COUNSELLING  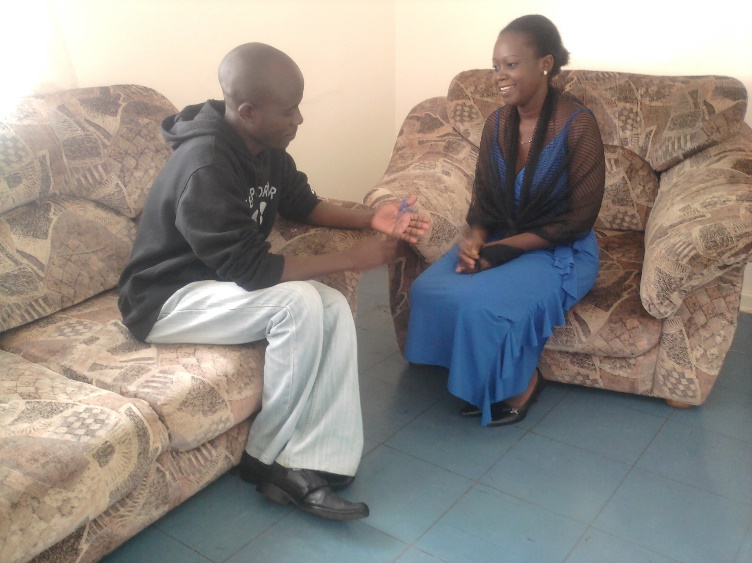  TRAINING RESOURCE MANUAL  MOH  ©  (2015) |
| --- |

**Table of Contents**

*List of abbreviations and acronyms ……...…………………………………………….… i*

*Foreword .………………….………………………………………………………………… ii*

*Acknowledgments ….…………………………………………………………………...…. iii*

*Introduction ………………………………………………………………………………… iv*

**CHAPTER ONE:** Facts about HIV and AIDS …………………….…………………….…… 1

**CHAPTER TWO:** Antiretroviral Drugs (ARVs) ……………….……………………………. 3

**CHAPTER THREE:** Prevention of Mother to Child Transmission of HIV ……….………… 7

**CHAPTER FOUR:** HIV Testing ……………….…………………….……………….……… 9

**CHAPTER FIVE:** Introduction to Counselling ……………………………………………… 11

**CHAPTER SIX:** Self-Awareness and Human Development ……………….…………...…… 17

**CHAPTER SEVEN:** Professional and Ethical Issues ……………………………………...… 23

**CHAPTER EIGHT:** Counselling supervision ……………………………………………….. 25

**CHAPTER NINE:** Cultural, Traditional and Psychosocial Issues of HIV/AIDS ……………. 27

**CHAPTER TEN:** Counselling Skills ……………….…………………….………………….. 29

**CHAPTER ELEVEN:** Counselling Special Groups ……………….…………..…….………. 36

**CHAPTER TWELVE:** Human Sexuality ……………….…………………….…….…..…… 51

**CHAPTER THIRTEEN:** Male Circumcision ……………….…………………….….……… 53

**CHAPTER FOURTEEN:** Management of Counselling Services …………………………… 55

**CHAPTER FIFTEEN:** Communication Skills and Report Writing …………………………. 57

***Appendices***

*1. Verbatim writing format ............................................................................................. 60*

*2. Journal Writing writing format .................................................................................. 61*

*3. Vocabulary of feelings ................................................................................................ 62*

*3. Consent forms ............................................................................................................. 63*

*4. Assessment Guide for Psychosocial Counselling Training Session………..…………. 64*

*5. Time Table ……………………………………………………………………………………. 65*

***Bibliography…….………………………………………………….……………..……………. 66***

**Foreword**

The Ministry of Health attaches great importance to providing the nation with equity of access to cost effective quality and affordable health care services as close to the family as possible.

Counselling for HIV and AIDS is not a very new programme in Zambia, having been established in 1987 as part of the National AIDS Task Committee of the Ministry of Health. The task committee was later transformed into the National AIDS Prevention and Control programme (NAPCP) in 1989 and given a mandate to lead a national response to the threat posed by the HIV and AIDS epidemic.

A needs assessment conducted by the Ministry of Health informed the National AIDS Prevention and Control Programme that the provision of counselling services was key in addressing the psychosocial challenges related to effects of the increasing incidences of HIV infections in the country.

The initial curriculum used to train the first group of HIV/AIDS counsellors was developed by the World Health Organization (WHO) in Geneva for use by member countries around the World.

In Zambia, the curriculum has since been revised to pave way for the inclusion of up to date information on HIV/AIDS including the new trends and developments in the area of psychosocial counselling in the country.

The Ministry of Health, being a key partner of the Zambia Counselling Council (ZCC), has a very proud record of successfully collaborating with institutions within the government private sector to build capacities in the provision of psychosocial counselling services which include supervision and training.

Additionally the Ministry of Health has endeavored to promote the best practices during the training of psychosocial counsellors, peer educators and ART adherence supporters country-wide to ensure that the end users of these competencies and skills receive the best psychosocial care and support services.

The manual is an embodiment of learning materials collected from counselling experiences that span over twenty years. Readers are also encouraged to use other reference materials in order to expand their knowledge base of the counselling profession.

It is therefore envisaged that the readers will find useful the learning materials contained in this training resource companion.

Dr. Peter Mwaba

**PERMANENT SECRETARY**

**Abbreviations and acronyms**

AIDS Acquired Immune deficiency syndrome

ART Antiretroviral treatment

CHWs Community Health Workers

CSO Central Statistical Office

CTC Counselling, Testing and Care

DBS Dry Blood Spot

DHMT District Health management Team

EGPAF Elizabeth Glaser Pediatric AIDS Foundation

HIMS Health Information Management System

HIV Human Immuno-deficiency virus

KCTT Kara Counselling and Training Trust

MDG Millennium Development Goal

MOH Ministry of Health

MHUNZA Mental Health Users Network of Zambia

NAC National AIDS Council

DNA Deoxy-ribonucleic acid

NRTI Nucleoside Reverse Transcriptase Inhibitors

NNRTI Non-Nucleoside Reverse Transcriptase Inhibitors

PLWAs People living with HIV/AIDS

RNA Ribonucleic acid

UNAIDS United Nations Joint Programme on HIV/AIDS

UNZA University of Zambia

VCT Voluntary Counselling & Testing

WHO World Health Organization

ZPCT Zambia Prevention, Care & Treatment Partnership

ZCC Zambia Counselling Council

ZVCTS Zambia Voluntary Counselling and Testing Services

**Acknowledgments**

The development of this PSYCHOSOCIAL COUNSELLING TRAINING RESOURCE MANUAL has been made possible with financial support from Global Fund in collaboration with the Ministry of Health.

The compilations and illustrations used in the production of this manual were obtained from numerous sources with the help of technical input from the following individuals and organizations:

Mr. John Mayeya (*Chief Mental Health Officer*), Dr. Susan Strasser (*Country Director, EGPAF*), Ms. Neater Sialwiindi [Late] (*CT senior technical advisor, ZPCT*), Mr. Joseph Nyirenda (PMTCT/*CT senior technical advisor, ZPCT*), Mrs. Ruth Sanyanabe Ndopu (*child counsellor*), Mr. Christopher Mwila (*child counsellor*), Mrs. Veronica Tembo (*technical advisor, EGPAF*), Mr. Bristol Cheembo *(ZVCTS coordinator*), Dr. Wezi Kaonga (*STIs & HIV specialist*), Mrs. Veronica Muntanga (*HBC & HIV/AIDS officer*), Ms. Grace Mumba Tembo (*STIs & HIV/AIDS officer*), Ms. Martha Chilufya (*Director, Bolton Health Institute of Education*), Mrs. Mercy Ulaya (*PMTCT technical officer, NAC*), Mr. Stanley Chama *(CEO, Integrated Human Development Trust)* Mrs. Monica Jalasi *(OVCs programme officer, NAC*), The Zambia Counselling Council (ZCC), Kara Counselling and Training Trust (KCTT) and the Churches Health Association of Zambia (CHAZ).

The editing and alignment of the contents of this manual to its current form was done by Mr. Pascal Kwapa *(Principal Mental Health Officer)* and Mr. Friday Nsalamo *(Senior Mental Health Officer.*

Finally, the Directorate of Disease Surveillance, Control and Research coordinated the efforts to mobilize material and technical human resources needed for the production of this first edition manual for the training of psychosocial counsellors for the Ministry of Health.

Dr. Elizabeth Chizema

**Director, Disease Surveillance, Control and Research**

**Introduction**

This manual is intended to be used as a training resource manual for counsellor trainers and trainees who wish to enhance their competencies in the practice of counselling. It has been designed to provide reference tips to all those wishing to enrich their counselling skills and practice.

The manual may be used as a complete training package as well as modular training for tailor-made courses. The contents of the manual can be adequately covered within the eight weeks and twelve weeks periods stipulated for the training of counsellors at basic psychosocial and advanced certificate levels respectively.

When conducting training, it is strongly advisable to use well qualified trainers who meet the appropriate standards for training others as stipulated by the Zambia Counselling Council and the Ministry of Health Guidelines on HIV/AIDS Counselling in Zambia. Trainers are therefore strongly urged to employ various techniques such as demonstrations, role-plays, directed reading, assignments, group discussions and debates when conducting training for would-be counsellors in order to give the learners hands-on competencies in various counselling skills and techniques.

Recommended ways for conducting standard psychosocial counselling courses using this manual include, but are not limited to, the following:

1. Three phase courses;
   1. One week of introductory sessions to the concepts and skills of counselling
   2. Six weeks of supervised counselling practice in a recognized counselling and testing institution
   3. One week consolidation, feed-back from field practice and assessment
2. Two phase courses;
   1. Two weeks of trainer-trainee contact sessions for the theories, concepts and skills of counselling. Assessment of theoretical discourse is also done at the end of this phase
   2. Six weeks of supervised counselling practice in a recognized counselling and testing institution
3. Week-end courses;
   1. Four to five consecutive week-ends of trainer-trainee contact sessions for the theories, concepts and skills of counselling, with continuous assessment through individual and group assignments. Assessment of theoretical discourse is also done during the final week-end
   2. Four weeks of supervised counselling practice in a recognized counselling and testing institution

**NOTE:**

1. Trainees should be supervised by experienced and or trained counsellor supervisors.
2. Training of psychosocial counsellors should be conducted by qualified and registered trainers
3. All prescribed assignments should be successfully completed by a trainee prior to certification
4. Registration with relevant professional bodies such as the Zambia Counselling Council (ZCC), the Health and allied professions council etc. should be encouraged and or facilitated by training institutions

# CHAPTER ONE: Facts about HIV and AIDS

The germ HIV that causes AIDS was discovered in 1981 by French and American researchers after a long period of search to find the cause of immune suppression in a number of gay people who presented mainly with pneumocystic carinii pneumonia (PCP), rare type of pneumonia and aggressive type of Kaposis sarcoma in California. Since the discovery of this deadly virus, over 70 million people are estimated to have been infected with the deadly virus. Over 35 million people have since died and currently and estimated 37 million are living with the HIV, two thirds of who are found in the Sub-Saharan African region (USAID, 2016).

**What is HIV?**

- HIV is a micro organism (germ) that destroys the immune cells of the body
- HIV belongs to the category of **single stranded** (RNA) viruses
- HIV is mutative in nature (no specific shape)
- HIV first invades the inside of the host cell (T-lymphocyte) and integrates its RNA into the DNA of host cell (**deprogramming**)
- It then makes copies of itself using the DNA in the host cell (**HIV replication)**

**AIDS** is a state of lowered immune response to infections and allergies and is characterized by reduced CD4 count, high viral load and presence of **opportunistic infections**.

**Opportunistic infections** are infections that take advantage of the lowered immunity to invade the body. Common opportunistic infections are: TB, Shingles (Herpes zoster), Cryptococcal (fungal) meningitis, Aggressive Kaposis sarcoma, Skin rashes, Diarrhoeal diseases, Severe Herpes Genitalis and herpes labialis

**Modes of HIV transmission**

1. HIV is transmitted mainly through unprotected, penetrative ***sexual intercourse*** with an infected partner. About 95% of new HIV infections in adults are transmitted sexually.
2. From ***infected mother*** to child during pregnancy, at birth or during breastfeeding. About 5% of new HIV infections are as a result of mother to child transmision.
3. Through ***contact with infected blood*** or blood products using contaminated syringes or sharp instruments or through organ transplants. Due to stringent measures applied in the storage of donor blood and blood products in hospitals, less than 0.1% of new HIV infections are as a result of using blood and blood products.

**Window period** is the period from the time when one is infected up to the time when the body produces *antibodies* and this is anytime from 2 weeks to 6 months. Since the common HIV test methods only detect the presence of antibodies for HIV, testing should best be done at least 3 months after suspected exposure to HIV, and repeated if necessary to allow enough time to check one’s blood for the presence of HIV anti-bodies.

**Incubation period** is the time it takes for a person who is infected with HIV to begin showing symptoms of AIDS. This is about 3 – 15 years in adults and about 2 months – 3 years in children.

**CD4** (clusters differential) count is a measurement of the number of T4 lymphocytes (immune cells) that are in the blood. The normal CD4 count of a healthy Zambian ranges between 500 and 1200 per microlitre (drop) of blood. It should be noted however that CD4 levels between 485 and 500 may also be normal for many HIV-infected individuals who have suffer from chronic stress, malnutrition, pregnancy, alcohol and drug abuse, TB, measles including cancers.

**Viral load** count is an estimation of the number of viral particles found in a drop of blood and a high viral load is and indication for antiretroviral drugs (ARVs). The viral load of an HIV infected person taking ***antiretroviral drugs*** is significantly lower than that of an HIV infected person who is not on treatment.

Antiretroviral treatment is capable of reducing the viral load to ***undetectable levels*** if the HIV infected persons follows treatment instructions (***adherence***).

#### Signs and Symptoms of AIDS

The World Health Organization has produced a clinical AIDS definition for Africa. In adults it is defined by existence of two major signs and one minor sign in the absence of other known causes of immune-suppression.

#### Major Symptoms

1. Loss of ten percent of body weight within a short period
2. Chronic diarrhea persisting for more than a month
3. Chronic fever persisting for more than month.

##### Minor Signs

1. Persistent cough for more than a month
2. Generalized Itchy lesion (dermatitis)
3. Recurrent herpes zoster (Shingles) commonly known as “*mulilo wa nyambe”, “vipyazimu”, or “umulilo wa kwa lesa”* in Zambian local languages.
4. Oral candidiasis (thrush)
5. Generalized Lymphadenopthy

NB: Presence of diseases such as aggressive Kaposis sarcoma or cryptococcal meningitis is usually indicative of very low immunity due to underlying AIDS. The diagnostic criteria in children are as follows.

Major Symptoms**:**

#### Failure to thrive or weight loss

#### Chronic diarrhea

#### Prolonged fever persisting for more than a month

#### Minor signs:

#### Generalized lymphadenopathy

#### Oral candidiasis

#### Repeated common infections

#### Generalized dermatitis

#### Confirmed maternal HIV infection

**WHO Clinical Staging of HIV/AIDS**

| Stage 1 | Stage without symptoms (asymptomatic). May last 2 – 12 years depending on a person’s life style and natural immunity. |
| --- | --- |
| Stage 2 | Characterized by minor symptoms which are on and off. |
| Stage 3 | Characterized by more serious (major) symptoms. ART should be instituted by this stage. CD4 count may be ≤200 |
| Stage 4 | Stage of advanced AIDS symptoms characterized by marked weight loss and ill health. CD4 count may be ≤ 50 |
| *Stage 5* | *Death* |

**Drivers for the epidemic in Zambia**

- Inconsistent use of condoms
- Intergenerational sexual activities
- Migrant workforce
- Alcohol and drug abuse
- Multiple sexual contacts
- Customary practices which promote multiple sexual partners
- Early sexual debut by young people
- Poverty
- Poor Health Systems
- Limited Resources for Prevention and Care
- Gender Inequality, Denial and Stigma
- Prevalence of STIs

**Strategies used to mitigate the spread of HIV in Zambia**

In Zambia, the Ministry of Health has partnered with local and international NGOs including line ministries and the National AIDS Council (NAC) to coordinate the effort in mitigating the impact of HIV and AIDS in the country.

This collaboration has led to the reduction of HIV prevalence from 21% in 1996 to 11.3% by the year 2016. The interventions include the following:

- Provision of antiretroviral therapy
- Promotion of HIV Counselling and Testing services in all the districts to ensure prompt treatment with ART
- Prevention (elimination) of mother to child transmission of HIV (PMTCT) with ART
- De-stigmatization of people living with HIV
- Condom promotion
- Promoting Voluntary Medical Male Circumcision (VMMC)
- Cervical cancer screening
- Diagnostic counselling and testing for HIV
- Provider Initiated HIV counselling and testing
- Health education
- Advocating for the rights of PLWAs
- Early treatment of TB & STIs

**CHAPTER TWO: Antiretroviral Drugs (ARVS)**

These are drugs that are used to treat AIDS symptoms. When properly used, they improve the quality an HIV infected person’s life thereby enabling the person to become healthy and productive. Antiretroviral drugs should be taken every day for life, following the clinicians’ instructions. They have side effects but these do not last for a long time and can easily be dealt with if one follows instructions on how to deal with them.

# How do ARVs work?

1. ARVs reduce the viral load: (Stop HIV from invading immune cells, Slow down the viral replication in the infected immune cell)
2. ARVs improve the immune functioning
3. ARVs reduce the occurrences of opportunistic infections
4. ARVs increase vitality
5. ARVs improve the general health status of an individual

# Goals of ART

- Maximal and durable suppression of HIV replication
- Restoration and preservation of immune function
- Restoration of normal growth and development in children
- Reduction of HIV related illnesses and death
- Improved quality of life
- Back to school, work, business.

# When should ART be considered?

- When the HIV antibody test is positive.
- All pregnant HIV positive women should start antiretroviral therapy regardless of the CD4 count.
- All HIV positive children from birth – 19 years should be commenced on ART immediately and regardless of the CD4 count
- In the absence of CD4 count equipment, urinalysis should be done and if no protein is found, ART can be commenced.
- Before commencing ART, ensure that other medical problems and opportunistic infections such as TB, PCP, malaria have been adequately addressed or treated.
- When potential adherence barriers have been assessed and addressed
- When the client and his family are motivated and ready
- When stable drug supply is assured

**When NOT to start ART**

- When HIV negative
- When potential adherence barriers not assessed
- When patient/family not motivated/ready
- When stable drug supply not assured
- When other medical problems create risk: e.g. active, untreated TB or acute PCP

**Advantages of Starting ART** Earlier:

1. You prevent CD4 decline,
2. You prevent escalation of opportunistic infections,
3. You protect the brain & other vital organs,
4. You preserve immune response to HIV (HIV immune response does not improve on therapy)
5. Children generally respond very well to ART

**How good must adherence be?**

- Generally > 95% of doses
- Some treatment regimens are more “forgiving” than others: D4T/3TC/NVP is a less “forgiving” regimen- but can work excellently for years if adherence is maintained.
- Missing 1 dose per week is 93% adherence
- Adherence < 80% almost always fails
- “Good” adherence taking “most” doses will lead to failure
- Rare missed dose is tolerated

**TARGET SITES FOR ARV DRUGS**

**HIV particle**

**Injection**

**of**

**contents**

**HOST CELL**

**Binding**

**Binding**

**sites**

**RNA**

**DNA**

**Reverse**

**transcription**

**Transcription**

**Integration of provirus**

**DNA into host DNA**

**Translation**

**Cell**

**membrane**

**Completed**

**HIV particle**

**Maturation**

**Budding**

**Viral**

**assembly**

**Protein**

**cleavage**

**gp41**

**gp120**

**RNA s**

**e**

**Protease**

**Integrase**

**Provirus**

**(circular**

**structure)**

**Protease Inhibitors work here**

**NRTI’s & NNRTI’s work here**

**Fusion inhibitors work here**

CD4 Cell

HIV Particle

**Integrase Inhibitors**

**Classes of Antiretrovirals**

Nucleoside Reverse Transcriptase Inhibitors (NRTIs)

- Zidovudine (ZDV or AZT)
- Didanosine (ddl)
- Stavudine (d4T)
- Lamivudine (3TC)
- Abacavir (ABC)
- Emtricitabine (FTC)

Non-Nucleoside Reverse Transcriptase Inhibitors (NNRTIs)

- Nevirapine (NVP)
- Efavirenz (EFV, also known as EFZ)
- Delavirdine (DLV)

Protease Inhibitors (PIs)

- Tenofovir Disoproxil Fumarate (TDF)
- Indinavir (IDV)
- Ritonavir (RTV)
- Lopinavir (LPV)
- Nelfinavir (NFV)
- Saquinavir (SQV)
- Atazanavir (ATV)
- Amprenavir (APV)

Entry (Fusion) Inhibitors and Other New Classes

- Enfuvirtide (T20, also known as Fuzeon®)

# Important considerations when taking ART

1. ART is life long
2. Requires adherence and discipline (compliance & commitment to treatment)
3. Regular medical check-up for infections, allergies, drug interactions. e.t.c
4. Regular biochemical check-ups:

- Full Blood Count
- CD4 count
- Viral load count
- Liver Function Test
- Kidney Function Test.

1. Requires good diet, plenty of fluids and rest
2. Should use condoms to reduce chances of re-infection and cross resistance of ARVs.

# Causes of ARVs drugs resistance

1. Incomplete treatment
2. Inadequate dosage
3. Mutation (changing characteristics of the HIV virus).
4. Mono therapy (using a single drug)
5. Resistant HIV strain

# Common side effects of ARVs

- Stomach upset leading to nausea, vomiting, diarrhoea or constipation
- Anemia (associated with AZT)
- Skin rashes
- Dizziness and or drowsiness
- Headaches
- Increased appetite
- Bad dreams (associated with Efivarence)
- Anxiety and dysphoria (associated with Efivarence)
- Rheumatism (associated with Lamivudine)

**Immune reconstitution inflammatory syndrome (IRIS):** A re-establishment of immune reactions to dormant opportunistic infections characterized by symptoms of general ill health e.g. headaches, diarrhoea, high fever e.t.c. To avoid IRIS, all clients who are eligible to ART must be thoroughly screened treated for OIs prior to initiating ART.

**Special Considerations for ART in Children**

- Consider the palatability (taste of drug)
- Syrups are generally bulkier- Fluid volume, refrigeration
- Ability to split, crush, mix and administer tablets/capsules
- Potency, pediatric experience, pharmacokinetic information
- Regimen complexity - dosing frequency, food and fluid requirements
- Presence of other infections that could affect drug choice - TB, Hepatitis B or C or chronic renal or liver disease

**Steps to ART success in children**

1. Identify child for whom benefits outweigh risks (and local guidelines permit therapy)
2. Assess prior adherence, all potential adherence barriers
3. Implement solutions to adherence barriers
4. Educate family and child about HIV & HAART
5. Select treatment that is potent, durable, convenient, non-toxic, well-tolerated, and sustainable
6. Train family and child on dosing and schedule
7. Monitor response and adherence
8. Respond promptly to problems

Viral Load: You want the viral load to be LOW! If your T-cells are high and your Viral Load is low, you can be healthy for a very long time

Post Exposure Prophylaxis (PEP): This is short term antiretroviral therapy that is given to an individual who has been exposed to HIV through accidents such as needle pricks including unprotected sex due to rape, child sexual abuse or sexual assault. PEP works by preventing HIV replication in an exposed person, thus preventing the HIV infection from becoming established. ARVs are administered for a period of 3 – 6 months to HIV negative victims after the following eligibility procedure has been undertaken:

- Immediately after the accident, victim should wash thoroughly with soap and running water
- Do HIV test immediately to verify current status
- A positive result means victim was positive even before the accident happened
- A negative result is indication for eligibility to receive PEP

**Pre Exposure Prophylaxis (PrEP):** WHO (2013) recommends that sexually active HIV negative individuals or those who are in a discordant relationship may take ART especially Truvada to protect themselves from acquiring HIV from their partners. This is known as Pre Exposure Prophylaxis.

NB: **It is important to point out that ART regimes are under constant modifications, as such the regimes outlined above may be replaced by new ones in the next few years**.

Methods of Risk Reduction: In Zambia, the most common mode of HIV transmission is heterosexual sexual intercourse followed by mother to child infection. Steps that people can take to reduce the risks of HIV transmission:

- Abstaining from sex unless one can effectively use condoms.
- Using condoms consistently whenever they have sexual intercourse even with a trusted partner
- Reducing the number of sexual partners to just one, but still using condoms unless their HIV status (negative) is verified.
- Avoiding sex with people who may have multiple partners.
- Visiting the antenatal clinic as soon as one discovers that she is pregnant
- Being faithful to one sexual partner and ensuring that each partner is safe and protected
- Teaching adolescents about reproductive health issues and not waiting until they much older

**CHAPTER THREE: Prevention (elimination) of mother to child transmission of HIV (PMTCT)**

The rate of Mother-to-child transmission (Vertical transmission) in an Untreated Breastfeeding Population is up to 30 - 45%

- Pregnancy (womb): 5 - 10%
- During birth: 10 – 15%
- Breastfeeding: 5 – 20%

**Three Objectives of the next Five Year PMTCT Scale-Up Plan in Zambia**

(Virtual Elimination of MTCT of HIV and provision of care and treatment for pediatric HIV)

- To reduce the transmission of MTCT to HIV to less than 5 percent by 2015
- To reduce the unmet needs for family planning by 50% from the current levels of 27% by 2015
- To provide antiretroviral therapy to at least 95% of HIV positive children in need of treatment by 2015

**Scaling Up PMTCT Services** requires the following strategies:

- Maintaining above 90% of antenatal clinic (ANC) utilization
- Increasing the percentage of women attending the first ANC by 14 weeks gestation
- Improving acceptance of testing to 100%
- Improving adherence to antiretroviral therapy (ART) by HIV positive women to 90%
- Increasing the proportion of women delivered by skilled health workers from 47 to 70%

# Drugs for PMTCT:

**For prevention of Mother to Child transmission of HIV, the following treatment methods may be adopted**

Eligibility Criteria for Initiating Antiretroviral Treatment (ART) or Prophylaxis in HIV Positive Pregnant Women Based on CD4 Cell Count and WHO Clinical Stage.

**Points to note:**

1. Assessment for ART eligibility for HIV positive pregnant women should be initiated in the Maternal Child Health (MCH) department before referral for enrolment into care.
2. All HIV positive pregnant women must be started on ART regardless of gestational age. This is for their own health and also intended to build the foundation for the delivery of a healthy baby. Antenatal care aims at making pregnancy and delivery a safe experience for the mother. All pregnant women are encouraged to attend the first antenatal visit as soon as they suspect that they are pregnant. They should attend at least four ‘focused’ visits, and more as required, to cover the following:
3. Clinical screening and examination, monitoring of blood pressure, urinalysis, and weight at each visit.
4. Active detection and effective treatment of Sexually Transmitted Infections (STIs). The Rapid Plasma Reagent (RPR) test kit is used for syphilis screening, and if positive benzathine penicillin 2.4 million units once only is used as treatment. If RPR is found negative in the first trimester, the test must be repeated at 36 weeks.
5. Prevention, detection and treatment of anaemia in line with Safe Motherhood guidelines: All pregnant women must be given daily supplements of ferrous sulphate and folic acid to prevent anaemia. Screening for anaemia preferably includes laboratory monitoring of Hb levels but can be done by clinical assessment if this is not available. Any clinically significant sign of anaemia should be routinely treated as part of ANC.
6. Pregnant or breastfeeding women should be started on a non-AZT containing regimen (AZT replaced by TDF) while the anaemia is being corrected. Systematic de-worming with mebendazole should also be provided to all pregnant women.
7. HIV testing: As soon as possible after confirming the mother is HIV positive, the woman should be engaged in comprehensive, integrated HIV care. She should be enrolled into continuous counseling activities that include adherence, infant and young child feeding, couples counseling (disclosure, partner testing, family planning), other aspects of the continuum of care such as early infant diagnosis, testing for all family members and HIV care and treatment.
8. Co-trimoxazole prophylaxis: Co-trimoxazole is recommended for all HIV positive pregnant women after the first trimester. The dose of co-trimoxazole for pregnant women is one double strength tablet or two single strength tablets once daily (total daily dose of 800mg sulfamethoxazole and 160mg trimethoprim).
9. Nutrient balance for the prevention of low birth weight to all antenatal attendees.
10. Counseling on infant and young child feeding options including the health benefits and challenges of each option so that women can make an informed choice.
11. Intermittent Presumptive Treatment (IPT) with Sulphadoxine-pyrimethamine (Fansidar®) for malaria prophylaxis. A dose of 3 tablets should be given starting in the second trimester. The IPT should be administered at least after every 4 weeks to ensure that a woman has at least three treatments before delivery. HIV Positive pregnant women receiving daily co-trimoxazole should not be given sulphadoxine - pyrimethamine for malaria prophylaxis. Co-trimoxazole has been proven to have prophylactic effect on malaria as well as other opportunistic infections and is sufficient in this case. All pregnant mothers must sleep under an insecticide treated mosquito net every night. Tuberculosis (TB) clinical screening in HIV infected mothers with history taking, examination and sputum smear if indicated. If diagnosed positive, refer for appropriate TB care.
12. Promoting and supporting couples counselling, partner disclosure and male involvement in antenatal care.
13. If indicated continuous, comprehensive HIV care should be provided at a minimum of every 4 weeks during the antenatal period, or sooner e.g. if ART has just been initiated, if there are signs of illness or medication toxicity and if there are adherence or other psychosocial issues that need closer follow up. This should include, but is not limited to, the following:
    - Repeat clinical assessment, including assessment of foetal well-being.
    - Follow up of lab results and repeat monitoring if clinically indicated and continue providing counseling.
    - At these visits the woman should be assessed for the presence of anemia (Hb estimation if necessary), assess adherence and other issues such as disclosure and side effects of drugs.
    - Messages such as infant and young child feeding, family planning, early infant HIV testing, HIV care and treatment and other aspects of continuum of care can also be emphasized.
14. Towards the end of pregnancy, pregnant women who live far from delivery centers should be encouraged to utilize waiting homes / mothers shelters where available.
15. Delivery options should be discussed with the woman. Elective caesarean section in combination with other PMTCT strategies is of value in reducing the risk of transmission where feasible and appropriate.
16. If chooses not to breastfeed, the health care professional must ensure they give full information on the replacement feeding with baby formula milk.

| **Sulphadoxine-pyrimethamine and co-trimoxazole are not given in the first trimester because both drugs have anti-folate properties that may cause fetal malformation** |
| --- |

1. In addition to the above interventions the following strategies are also considered equally important to consider:

- **Non-intrusive** delivery procedures, that is to say avoiding forceps deliveries
- Use of savlon, EUSOL and indeed any other **clinically proven antiseptic** to clean the vaginal area during delivery
- **Avoiding early rapture of membranes** also improves the chances reducing mother to child transmission of HIV
- Prevention and **treatment of malaria** during pregnancy in HIV infected improves the chances of reducing mother to child transmission of HIV

# Full ART: The option is supported in view of the fact that ART reduces the viral load and improves the general health status of the mother before she can consider conceiving. Many mothers may therefore opt to receive full ART before considering the prospects of conceiving.

**Caesarian operation:** This option greatly reduces the chances of Mother to child transmission of HIV by up to 1%. The only problem with this option is that there is not enough staff and theatre space for all women wanting to deliver by caesarian section.

#### CHAPTER FOUR: HIV Testing

HIV ***rapid tests*** work by detecting antibodies to HIV in the blood. Test methods include the following:

- Capillus HIV 1 & 2
- Abbott determine HIV 1 & 2
- Unigold HIV 1 & 2
- HIV spot check HIV 1 & 2
- Hema strip HIV 1 & 2
- Multispot HIV 1 & 2
- The Bionor

Other HIV tests which are ***more specific*** work by detecting HIV infected T-lymphocytes in the blood. These include:

- ELIZA Agglutination blot assays
- LIA-Line immunoassay
- Western blot
- Gene II. HIV 1 & 2
- Polymerase chain reaction (P.C.R)
- Indirect Immuno florescent assay (IFA)
- Viral load

**Confirmatory Tests:** Confirmatory test can also be done by doing two different rapid test on one sample. PCR method can be used if a client is suspected to be in the window period or testing children. Below are the confirmatory tests:

- Western blotting/ELIZA
- PCR
- Culture
- R.I.P.A-Radio immuno-precipitate assay.
- Bionor

**GENERAL PRINCIPLE OF HIV TESTING**

This is based on demonstrating the presence of HIV antibodies in a given sample. Presence of the antibodies means the test is positive to HIV and absence of antibodies means Negative.

- **Positive Result (Reactive):** There is a reaction between Antibody + Antigen (AbAg )
- **Negative result (non- reactive):** There is no reaction between Antibody + Antigen (Ag )

**Polymerase Chain Reaction Test (PCR): Principle:-** These are based on quantitatively identifying the presence of infected T. lymphocytes. The poly virus DNA

**Samples for HIV Testing:** These should be any one of the following:

- Whole blood
- Plasma
- Serum
- Saliva

**Test One: Abbott Determine HIV 1 & 2**

- An in vitro, visually read, qualitative immuno assay for the detection of antibodies to HIV 1 and HIV 2. In human serum, plasma as whole blood.
- The test as intended as an aid to detect anti bodies to HIV 1/ HIV 2 from infected individuals

**Procedure**

- Determine HIV 1 / 2 Is an immuno chromatographic test for the qualitative detection of anti-bodies to HIV 1/ 2
- Sample is added to the sample pad
- As sample migrates through the conjugate pad, it reconstitutes and mixes with the selenium colloid-antigen conjugate.
- This mixture continues to migrate through the solid phase to the immobilized recombinant antigens and synthetic peptides at the antigen window site
- Wait for 15 minutes
- If antibodies to HIV-1 or HIV-2 are present, a REACTION shows in form of 2 red lines and the results is Reactive or Positive.
- If antibodies to HIV-1 or HIV-2 are absent, there is NO REACTION and the result is Non-Reactive or Negative.
- To ensure assay validity, a procedural control bar is incorporated in the assay device

**Storage**

- Store ABBOT DETERMINE at 2 - 30° until expiration date
- Store GENIE II at 2 - 8° celcius
- Do not store beyond expiration date

**Specimen Collection**

- Plasma serum and whole blood collected by venus-puncture in EDTA bottles may be used
- Whole blood collected by finger prick may also be used

**Specimen Storage**

- Serum or plasma should be store at 2-8°C if test is to be run within seven days. If testing is delayed specimen should be frozen (-20° or colder)
- Whole blood collected by venus-puncture, should be stored at 2-8°C, if the test is be run within seven days of collection. DO NOT FREEZE WHOLE BLOOD SPECIMEN

#### Test Results

1. Positive results- Means that person has been exposed to HIV infection.
2. Negative results- Means that either a person has not been exposed to HIV infection or may be in the window period.
3. Indeterminate result- Means the result is equivocal, i.e. neither positive nor negative. The test needs to be repeated
4. False result – This result is due to technical error in interpreting/documentation of results on the part of person conducting the test or as a result of using expired test kits.

**Diagnosis of HIV Infection in Children**

Diagnosis may be made at two levels:

***Clinical diagnosis*** – based on the symptoms and signs the child presents with. This should always be confirmed by laboratory tests.

***Laboratory diagnosis***

- To confirm suspected HIV infection in a sick child or
- To determine HIV infection or exposure status in a child with no symptoms

**Laboratory Diagnosis**

There are two types of laboratory tests for HIV diagnosis:

(1) ***Antibody tests*** (identify antibody that the human body produces against the HIV):

- HIV ELISA, Western blot (performed in laboratory, 4 hrs)
- Rapid tests (e.g. determine, bioline, unigold – performed by lab. or counselor, may take 15-20 minutes to have results)

(2) ***Virologic tests*** (identify HIV in blood): HIV PCR (DNA or RNA/viral load assay), **DBS** method now being used increasingly.

**Laboratory diagnosis in children**

< 18 months age

*Antigen (viral) tests*

- DNA PCR (whole blood or DBS)
- RNA PCR

> 18 months age

*Antibody (serology) tests*

- - Rapid
  - ELISA

**HIV Tests and placental transfer of antibodies**


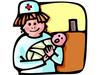


**Placenta**

**Unborn fetus in utero**

**************************

********

**Newborn**

**Maternal IgG antibodies**

**Therefore, all newborns are born with maternal IgG antibodies**

*(Follow the laboratory test algorithm in your setting/country)*

CHAPTER FIVE: Introduction to Counselling

Counselling has been practiced for as long as mankind has been in existence. Two major types of counselling can be described as the ***customary type*** and ***professional (modern) type***.

The former is the oldest type of counselling and currently practiced by traditional counsellors who, in customary circles, are known as *alangizi*, *bashibukombe, ankoswe.* Customary counsellors are selected based on socially approved personal attributes such as a successful marriage, religious affiliation, and personal character to mention just a few.

Professional counselling has also been recognized in Zambia and its impact in the mitigation of psychosocial problems such as HIV/AIDS, gender based violence, child abuse including other issues has been documented in works carried undertaken by human service agencies world over.

Carl Rogers contributed greatly to what is today considered modern counselling. Notwithstanding the complex nature life today, professional counselling is systematized and carried out more scientifically with the application of professional skills and techniques.

**Definition of Counselling:** Counselling is a process by which a TROUBLED PERSON (**client**) is helped to feel and behave in a more personally satisfying manner through interaction with a SKILLED HELPER (**counsellor**) who provides information and reactions which stimulate the client to develop behaviors which enable him to deal more effectively with himself and his environment (Lewis, 1970)

Several words in this definition are important. The word "process" the connotation to be deceived from this factor is that counselling is progressive, dynamic, methodical and employs special skills and techniques.

- The word "**process**" means that counselling is on-going, progressive, dynamic, and employs special skills and techniques.
- The word "**help**” means willingness to facilitate meaningful change and motivate client think rationally. The role of the counsellor is that of a helper, facilitator and motivator.
- The word "**interaction**” refers to the dialogue, relationship and communication between the counsellor and the client.

**Counselling Theories:** The profession of counselling has been developed from a number of theories and principles such as the Humanistic theory, Psychoanalytic theory, Behavioural theory, Cognitivist theory including the Eclectic (combined) approach to counselling.

Psychoanalytic Theory according to Sigmund Freud: The theory asserts that human behaviours are motivated by underlying unconscious processes and mainly influenced by childhood experiences.

According to Freud, the bulk of these unconscious processes emanate from a range of repressed sexual conflicts mainly from the formative stages of psychological development of humans.

Freud has postulated some stages of human development from birth up to old age and states that if an individual misses experiencing any one of these stages; h/she is likely to display some behaviour abnormality later in life: **Fixation.**

Behavioural Theories: The theory according to ***Albert Ellis*** is that ‘it is not the action (event) but the belief (value) that makes a person react/behave the way they do (consequences)

- A - Action (events).
- B - Belief (values)
- C - Consequences (response).

The other Behavioural theory according to ***B.F. Skinner*** is that since most of the behaviours are learned, therefore they can also be unlearned.

He demonstrated this using ‘Conditioning’. That is to say if human beings are continually exposed to certain situations, they eventually become accustomed (conditioned) and will no longer have to think about the situation before reacting to it [Experiment of the dog, food and bells (Association learning)]

He therefore believed that even if behaviour is difficult to change, they can change either through training/retraining, rewards or punishment – compliment clients for coming.

Humanistic theories: Humanists see it as essential to trust clients to follow their own self-discovery, at their own pace and direction. Notable among those who contributed to the development of the humanistic theory is Carl Roger who perceived humans being worthy of dignity, self-direction. He asserted that all humans are born with a manifold of inert potentialities and that the realization of their life goals depended very much, also, on the emotional climate surrounding their environment and upbringing.

Humanistic Theory according to Carl Rogers: The theories developed by Carl Rogers have been adopted as suitable for counseling purposes in Zambia.

Carl Rogers developed the ***person-centered*** approach that promotes the following attributes:

- Uniqueness of individuals: No two people are the same – even identical twins are believed to be different from each other.
- People respond according to their threshold (tolerance). Some people have low thresh hold while others have higher threshold to situations such as life problems or physical pain; aggressive psychopaths tend to exhibit a higher thresh hold to pain.
- Human beings are rational and capable of taking responsibility for themselves
- Autonomy (being in charge of themselves) should be respected.
- Humans have the ability to determine own destiny
- Humans have the innate potential to learn and attain goals.

**Approaches To Counselling According To Carl Rogers**

Non-directive approach**:**

- Acceptance of the client
- Establishment of a positive non-judgemental climate
- Trust in the client's wisdom, autonomy and permissiveness.
- Helping client to perceive himself and problem clearly (clarification)

Client-centred approach :

- - Help client to recognize and acknowledge his feelings, beliefs, and experiences, and how these impair or sustain normal functioning (Feelings).
  - Recognizing client as a person
  - Resolving incongruities between the "ideal self" and the "real self"
  - Empowering client to take responsibility for defining goals and action

Person-Centred Approach**:**

- - Explore personal involvement
  - Explore interpersonal relationship issues
  - Explore present experiences ("here-and-now")
  - Self-disclosing role for the counsellor (self-disclosure and client praise)

Systemic Counselling Approach**:**

- - Systemic counselling is a relatively new form of counselling that is particularly useful in addressing problems affecting groups of people living together such as families.
  - Systemic counselling recognizes the fact that an individual’s problem often occur within a context such as the family, work place, school set up, relationship etc. The goals of systemic counselling are therefore mainly to assist families to function effectively and to empower them to view their concerns from multiple perspectives, and to generate solutions that are appropriate to their perspective needs.
  - Systemic counselling also recognizes the importance of interdependence between individuals and the people that surround them.

**Basic Features of Counselling**

Counselling is an art, in the sense that it deals with practical life issues that affect people from all walks of life. Those practicing counselling should have certain values, skills and competencies required to practice the art (ZCC, 2003).

*Purposeful*: This feature refers to the need for counselling. Counselling is said to be purposeful essentially because it is always at the request of the client or by referral. It is entered into following an agreement between the client and counsellor.

*Privacy:* This Refers to the need for privacy in the counselling process. It essentially relates to the professional boundaries in the counselling interaction such as sitting distance, manner of addressing the client, bodily attractions (i.e. type of dress or make up) occupational background and respect for the client. The interaction is purely personal and should be treated as such. The client requests for counselling help in a personal capacity, the agreement is between one person (the client) and another (the counsellor) and not between one person and several others. The concept of privacy also refers to the location, i.e. venue or room where the counselling takes place. The location should be conducive for counselling and for maintaining confidential the counselling context. The room itself should be quiet and free from disturbances or frequent interruptions.

*Confidentiality:* Counselling is a helping relationship, which often involves clients in revealing information about their current and past situations, their opinions and innermost feelings. Confidentiality entails entrusting information to another person with the expectation that it will be kept private, secret and not divulged to a third party.

Should the need to breach confidentiality occur, the client must be reasonably and adequately informed by the counsellor about the nature and reasons for disclosure. It is always advisable to make thorough consultations with a counselling supervisor or an experienced counsellor and to obtain the written consent from the client.

*Autonomy*: Refers to the individual’s capacity to think, decide and act freely and independently. It is not simply doing what one wants to do, but is doing so on the basis of thought or reason. Autonomy is the cornerstone of the client's freedom to voluntarily participate or decline to participate in counselling. Because of this, the counsellor is responsible for working in ways which promote the client's control over his/her own life, and respect the client's ability to choose, make decisions and change in the light of his/her own beliefs, values and circumstances.

*Relationship*: A mutually acceptable interaction and relationship between the client and the counsellor is the basis of counselling. The relationship between counsellor and client should be a therapeutic one and nothing short of that. At the end of the counselling session the client should feel helped in one way or another. Therefore, counselling should essentially be based on mutual trust, honesty and acceptance. It should not be exploitative i.e. the counsellor should not gain at the expense of the client or vice versa. In other words, the relationship is not for sexual gratification, favours or material gain.

**The Therapeutic Relationship**

The success of any counselling intervention depends to a great extent on the following Core Conditions of Counselling: Genuineness, empathic understanding of the client and an unconditional positive regard of the client.

A mutually accepted relationship in counselling entails that the counsellor should exhibit a reasonable degree of personal qualities or attitudes in relation to the client. These would be expressed by way of her feeling (affect), reactions towards the client (behaviour) and thoughts or beliefs (cognition) about the client. How the counsellor responds to her client has a lot of meaning to and can be interpreted in different ways by the client. Hart & Tomlinson in MOH (2001) further amplifies the three (3) personal qualities or attitudes of a counsellor that impinge upon the counselling relationship as follows:

*Genuineness (Congruence)*: The counsellor should be real, and not hiding behind a mask or professional impersonality, or indeed, not merely playing a role. Genuineness also implies that the counsellor is aware of his/her competences and ability to interact not only with clients, but other people too. She wears many roles as a member of the community

*Empathic understanding*: Refers to the accurate moment by moment awareness of the client’s feelings, behaviour and cognition as expressed and experienced in the "here and now" frame of reference. It also refers to the counsellor's ability of intellectual and emotional identification with the client as well as appreciations of the client's problem situation. While mingling freely in the client's perceptual world, the counsellor must stand aloof to master objectivity. Accurate empathy makes the client understand and accept it as a true reflection of his present and experience. The counsellor needs to be sensitive to what is currently going on in the client and of the meanings which are just below the level of awareness. Accurate empathy coupled with genuineness enables the client to explore freely and deeply and, thus to develop a better understanding of himself and his concerns.

*Unconditional position regard (acceptance)*: Refers to a condition in which the counsellor values or prizes all aspects of the client, including his conflicts and inconsistencies, good and bad points, or parts appear wrong in the eyes of society. It is an unconditional positive regard or respect for the client as a person of worth. The counsellor must exhibit a deep and authentic caring for the client in his present situation, in a non-judgmental manner and a high sense if willingness to assist the client. The counsellor should not manipulate the client into behaving in a way deemed acceptable to him/herself.

**Counselling contract**

It is important to agree on the dos and don’ts of the counselling relationship with your client. The contract outlines the relationship boundaries and helps the client and counsellor to be focused on the therapeutic relationship.

The contract essentially spells out counsellor-client obligatory issues such as the problem a hand, confidentiality, number of sessions, when to meet, when to terminate the on-going counselling, issues of gifts and presents, payments, who to tell, who not to tell, “penalty” for disrespecting contract and so on and so forth.

**The Counselling Process According To Gerald Egan**

Gerald Egan developed the three staged counselling process as follows:

*Current scenario*: This is the stage at which the client is given space to elaborate his/her problems and during which the counsellor clarifies issues. It is also known as the story telling or problem identification stage.

*Preferred scenario*: During this stage the counsellor helps the client to think of possible solutions or options that would assist resolving the problem at hand.

*Action plan*: This is the implementation stage during which the counsellor helps the client to plan how he/she is going to go about working on the problem. The counsellor also ensures that the client is committed to the action plan

**Counselling Interventions**

*Decision-making*: As the phrase implies, decision-making is applied when the client is not able to make choices regarding his/her immediate situation. To help such a client move forward, s/he made to list the choices in a chronological order of importance, and then using a process of elimination, assisted to eliminate those choices that appear to have more harm attached to them.

*Problem-solving*: Very similar to goal-setting in that the client chooses goals that s/he thinks will make life more meaningful. And then using the three stage model, the client is helped arrange the goals in chronological order of importance so that h/she can focus only on those goals that appear more realistic to achieve. The problem-solving model is often applied in alcohol and drug abuse counselling.

*Crisis intervention*: This is also known as crisis management and is used to help a client who is experiencing very difficult situations that need urgent attention.

**Qualities of a Good Counsellor**

- A good counsellor should be knowledgeable and skillful
- A good counsellor should be warm hearted and friendly
- Counsellor should be deliberate-not hasty but thoughtful and quiet
- Counsellor is empathic-not disregarding, not detached
- Counsellor should not be biased/judgmental/condemning
- A good counsellor should be able to interpret issues critically
- Counsellor should be objective- not to be emotionally involved (Compassionate people should guard against this)
- A good counsellor should be realistic; knowing that it is not always that he or she will get positive results
- A good counsellor should be authentic, genuine and not artificial i.e. accept personal and professional limitations
- A good counsellor should be open minded and not defensive
- A counsellor is warm, friendly with the heart to help
- A good counsellor should be even tempered and honest
- A good counsellor should be sober minded and a role-model
- A good counsellor should be a good listener

**What counselling is Not**

- Counselling is not a mutual friendship but a problem-centered and goal-oriented interaction
- Counselling is not a conversation. It is not simply two people exchanging information and opinions
- It is not a discussion differing points of review are not being argued
- Counselling is not an interrogation. The client is not being questioned in order to find out the truth
- Counselling is not discourse by the interviewer- The counsellor does not use counselling as a forum in which to listen to himself speak or air his own opinions
- Counselling is not a confession, the client is not being morally evaluated, pardoned, or absolved by the counsellor
- Counselling is not a search for a diagnosis- The client is not questioned so that the counsellor can find a label or diagnosis for their problem

**The Dos and Don’ts of counselling**

- Always enter into a counselling contract with your clients to avoid unnecessary
- Do offer your services to clients but do not force them to be counselled by you!
- Do not criticize your clients, accept them the way they are
- Clients who come for counselling in a drunken state should be encouraged to come when they are sober
- Do not connive with your clients; instead help them to face the reality no matter how painful it might be because even a painful situation will soon come to pass.
- Do not have dual relationships with your clients; the counselling relationship should always therapeutic.
- Do not date a client (old and new); it is not morally right
- Avoid giving clients personal details of your life, your private mobile phone number or home address. If your clients request your phone number, you may give them the office phone number which they should use to call you during work hours. However, there may be a few situations in which clients may need your private mobile phone number such as clients who are in need of constant surveillance by the therapeutic team.
- Avoid gifts and presents from clients if you can; these can compromise the integrity of your practice. The issues of gifts and presents should be outlined within the contents of your contract with the client at the beginning.
- Do not test clients for HIV without their understanding (pre test counselling) unless their competency to give such consent is compromised by serious mental disorder, coma/unconsciousness or other serious illness. In such cases however, the next of kin should be counselled and empowered to give signed consent on behalf of the incompetent client and this should be in the client’s best interest. The details of such actions should given to the client as soon s/he recovers enough to understand the circumstances.

**Pretest counselling**

This is the counselling that is done before the test is carried out to prepare the client psychologically for the outcome of possible test results.

How to conduct pre test counselling

- Prepare the counselling room in a well-ventilated room that assures privacy, away from noise and other disturbances. Avoid conducting counselling sessions next to a busy OPD or antenatal view.
- Welcome the client and make him/her to feel comfortable
- Initiate introductions
- Get to know the client better first before attempting to discuss anything
- Explain the services that you provide and emphasize on confidentiality
- Establish the reasons for coming (Do not assume that all clients come for tests)
- If client wants an HIV test, find out what h/she knows about HIV and AIDS and fill in the knowledge gaps in a gentle and friendly manner
- Discuss modes of transmission and explain the importance of treatment with ART
- Discuss the test procedure, types of results and establish how the client would like to be given their results
- Establish how client will deal with either positive or negative results
- Discuss partner notification and help client to plan how h/she will disclose her status to a trusted person in his/her life.
- Address clients outstanding psychosocial concerns
- Allow time for questions
- Give appointment for the results

**Post Test counselling**

This is the counselling that is done after the client has been given his/her results in order to help the client come to terms with their results. It is recommended that results always be given out by the same counsellor who conducted the pre-test counselling.

How to conduct post-test counselling

Welcome the client back and make him/her to feel comfortable. Find out if the client has come to collect the results (DO NOT ASK IF CLIENT IS READY FOR RESULTS).

- If client has not come for the results, find out when they would want to know their results and encourage him/her to get results as soon as possible.
- If the client says s/he has come for their results then give them their results immediately.
- Allow time to absorb the result
- **If result is negative**, review the implications of negative results, discuss window period, encourage safe sex methods and other preventive measures.
- Suggest a repeat test after three months
- **If the result is positive:**
  - Find out what they mean to the client
  - Review the implications of a positive result
  - Explain difference between HIV infection and AIDS
  - Ask client about who he is going to share results with
  - Encourage the client to share results with his/her partner(s)
  - Discuss available treatment and other community support services, support groups including positive living
  - Discuss safe sex methods and encourage condom use
  - Emphasize the need for ongoing medical review and supportive counselling
  - Discuss client’s immediate psychosocial concerns and future plans
  - Refer client to other available services
  - Arrange for next visit

**CHAPTER SIX: Self-Awareness and Human Development**

**Introduction**

Self awareness is a very vital life awareness skill. It is intended to give participants an opportunity to explore and discover more of who and what they are. The topic also helps the counsellor to explore his/her personality or character (conscience) with a view to understanding one’s strengths and weaknesses.

**Meaning of self-awareness**

- Knowledge and understanding of oneself (strengths and weaknesses)
- Insight into one’s character and personality traits
- Examination of how one’s character affects others
- Self-awareness is important for all counsellor practitioners because you can only provide good counselling if you know yourself better first.

What is the importance of self-awareness to counsellors? Knowledge about what is going on around your space (feelings, thoughts, ideas, personal attitude, beliefs and behaviour) helps you to navigate your way around issues that affect the you think, feel, believe and behave.

- If we are aware of pain we want to do something about it, be it physical or emotional.
- Self-awareness helps us to be drivers of our lives, rather than passengers.
- If we are aware of how we feel, we can deal with these feelings, rather than suppress them and then let those feelings unknown to us, drive us to some actions which are either not acceptable to us or others.

**Feelings**

- We are human beings and we are alive to the extent we are capable of experiencing a variety of feelings.
- All feelings are natural and neutral.
- There is nothing good or bad about them.
- They can be pleasant or unpleasant.
- The outcome of certain feelings can be helpful or harmful, depending on how we deal with them. For example, dealing with anger

We therefore need to:

- Identify our feelings
- Know the feelings;
- Name it
- Claim it
- Tame it
- Aim it
- Don’t ASSUME!

Feelings are supposed to be dealt with each time they come in a positive way. When feelings are not attended adequately or immediately, they become unfinished business

**The Unfinished Business**

Every time we deny a feeling, it becomes unfinished business. It doesn’t go away, it waits to re-appear. Soon or later unfinished business demands attention. If we don’t express feelings directly, they have a way of coming out sideways and unexpectedly.

We say something mean or foolish or we act inappropriately. The point is we are not living in the present but we are acting out old emotions.

Unfinished business can also affect our health, unfelt feelings can lodge in our muscles, tissues and nervous system and if not expressed can eventually make us sick. Fortunately we have an indicator of unfinished business which is FEAR. We live in fear because we don’t deal with feelings effectively.

**Personality and behaviour:** Personality is the sum total of one’s character: temperament, attitude, likes and dislikes, habits including genetic traits. **Behaviour** on the other hand is any observable activity of an organism (humans included) such as walking, jumping, eating, smiling, crossing one’s legs e.t.c.

**Types of personality according to Hippocrates**

- **Sanguine**: Extrovert (outgoing and sociable), Talker (talks quite a lot) and Optimist (positive about the future)
- **Choleric**: Extrovert, Doer (practical), Optimist
- **Melancholic**: Introvert (in-door person), Thinker (analytic), Pessimist (low esteem)
- **Phlegmatic**: Introvert, Watcher (observer), Pessimist

NB: Which one of these personalities do you associate with?

**The self concept**

- Self-image: The type of person you perceive yourself to be e.g. “mother”, “pupil”, “fighter”, “pastor”, “leader”, “worker”, “lover”, “politician”
- Body image: how one views his physical body e.g. “slim”, “well built”, “beautiful”, “ugly”, “fat”,
- Self-esteem: The inner confidence that drives one to assert him/herself. People with low self esteem tend to withdraw or surrender easily
- Ideal self: What one wishes to become (fantasies, ambitions, dreams)

**Self-awareness window (JOHARI window)**

| **What I know and others know (public domain** **window):** Aspects of one’s character that is easily shared with others. | **What I don’t know, others know:** The things I do not know myself which others know. |
| --- | --- |
| **Only I know, others don’t (Private window):** What is only known to you, mainly secrets. | **Hidden window:** No one knows (blind spots, hidden abilities, dreams) |

**Ego states (psychic energies)**

1. Id: This is the impulsive and irrational psychic energy that animals (including humans) are born with. The Id controls basic instincts that humans are born with such as hunger, sexual impulses and aggression. The Id accounts for most of actions that occur before thinking and is also referred to as the animal in man.
2. Ego: As humans grow, interacting with parental figures and other social animals, they learn to control the Id impulses and the result of this is rational thinking [thinking before actions]. The psychic energy responsible for such refined behavioural and emotional state of affairs is the Ego.
3. Super ego: This is an advanced form of the Ego state that results into extreme control of one’s impulses to an extent that the person puts others before self (altruistic). Martyrs and saints can be likened to those with a super ego state of the mind.

**Ego boundaries and anxiety**

- **Anxiety** is the product of the conflict between the Id (emotional demands) and ego mind state (ability to resolve the conflict).
- The Id state of the mind always creates unnecessary emotional conflicts which results in feelings of anxiety as the ego attempts to resolve.
- People with loose ego boundaries lose tempers easily, cry easily and laugh very easily!

**Transactional analysis (TA)**

- **Child state** of mind: Since we were once children, we carry some aspects of the child state in us and this makes us to retreat to childhood tendencies (acting like kids) whenever we are in experiencing joy or trouble.
- **Parent state** of mind: Being brought up by parent figures teaches us to act like them at certain times especially when we have our own children or are teaching others.
- **Adult state** of mind: This is the rational state of the mind that we use whenever we are required to make an important decision for others.

Human Development

**Psychosocial Stages of Development according to Erik Erickson**

At each stage in their development, a person faces a psychosocial challenge. Erickson’s view is that the person has to master a challenge both socially and psychologically. Their success or failure in mastering the challenge of a particular stage has profound influence upon the later development and also upon how they live their lives and how they interact with others.

Developmental Tasks and Interventions for the eight Stages of Human Development:

Basic Trust versus Basic Mistrust: (Birth to 1yr - 6months)

*Task*: Children need to develop trust in their environment and in their parents and caregivers. Through their trust, children learn that their world is a safe, secure, consistent, predictable, interesting, friendly place.

*Interventions***:** Children need parents and caregivers with all the traits mentioned in the Task to help them achieve trust in their world and in others. Children need affectionate, consistent, predictable, and high-quality care to help them learn to bond with other people.

Autonomy versus Shame and Doubt (1 year – 3years)

*Task*: Children need to gain a sense of self-control as well as control over their environment.

*Interventions***:** Children need to experience success in doing things for themselves: expressing themselves, feeding, developing toilet behaviours, and performing various other motor tasks with hands and feet. Children often express their new feelings of autonomy by saying “no” to all requests and through frequent use of “me”, “mine”, and” I”. They respond well to choices.

Initiative versus Guilt: (3 years – 6 years)

*Task*: Children need to develop a sense of initiative as opposed to feelings of guilt about never doing the right thing.

*Interventions***:** Children need to begin setting goals, taking leadership, and carrying out projects. Parents need to empower children and let them participate in family work activities and projects. When children’s initiative carries them into unacceptable thoughts and behaviours, parents need to correct these behaviours in a loving, caring way as they teach their children what is and what is not acceptable. Discipline based on logical consequences should help these children develop a sense of purpose and goal-directedness.

Industry versus Inferiority: (6 years – 11 years)

*Task*: Children need to learn a variety of skills that will help them find a place in the adult world. The necessary skills range from academic and social to physical and practical.

*Interventions***:** Children need large doses of encouragement and praise to help them achieve the competence they need to eventually find a place in the adult world. Academic, physical, social, and work skills are all important in developing healthy self-esteem. Children need nurturing adults who will help them discover and develop their special talents and abilities.

Identity versus Confusion: (Age of puberty and early adolescence, 12 years - 18 years)

*Task*: teenagers need to develop a self-image. They need to know who they are and how their roles will fit into their future. This is the time for discovering ones identity as a young man or woman. This identity should already

*Interventions***:** Teenagers need to feel that others accept them as they work toward self-acceptance and a sense of identity. Identity can be found in joining a group or cause. Another way to achieve a sense of identity is to find things they do well in work and play. It is often good to permit adolescent time-out periods for self-study and exploration before making commitments to further education or training, jobs, careers, and marriage.

Intimacy versus Isolation: (Adult stage, 18 years- 25 years)

When the young person has reached a degree of self-assurance in relation to being a sexual person, then s/he is able to learn to develop intimate relationships, If not the person will remain in isolation, “a loner”

*Task*: The primary task in the young-adult stage is to achieve intimacy through sharing in a close friendship or love relationship. Middle adulthood tasks revolve around proper care of children and a productive work life. Older adults are concerned with ego integrity, which involves an acceptance of past life, a search for meaning in the present and continued growth and learning in the future.

Generativity versus stagnation: (25 years -55 years)

When the ability to know another person has developed, there is a giving life to others, either in the married state or in a helping occupation or simply through informal relationships, otherwise the person will be unable to contribute to the overall growth of human society.

Ego integrity vs. Despair (55 years onwards)

In the mature years of life, perhaps from 55 onwards, a person who has successfully completed all the tasks of the previous stages will be able to look back and integrate all her/his life experiences

*Interventions***:** Counselling interventions for adults are most effective when they match the client’s learning style. Client preferences for interventions are nearly equally divided between affective, behaviour, cognitive, and eclectic methods. Once again, we must remember that age does not guarantee that any particular stage of development has been reached. Many adults use concrete rather than abstract reasoning in solving problems and making decisions. Issues in counselling will often centre on relationships, careers, and the search for meaning and purpose in life.

##### Summary: Erickson helps the counsellor to understand powerful issues of a particular age that their client is seeking to resolve.

**CHAPTER SEVEN: Professional and Ethical Issues**

Professional and ethical issues are enshrined in the professional code of Ethics as outlined by the Zambia Counselling Council. The code of ethics outlines the fundamental values of counselling namely integrity, impartiality and respect, and a number of general principles arising from these. It addresses such issues as client safety, clear contracting and competence. Counselling is a non-exploitative activity and counsellors should take the same degree of work ethically whether the counselling is paid or voluntary

Counselors’ Responsibilities**:** To maintain effectiveness, commitment and ability to help clients.

**Maintain Confidentiality**

- Every care giver who practices counselling is required by the law to maintain the confidentiality of matters that are discussed with clients.
- Ensure adequate privacy before proceeding with the counselling sessions.
- Must not engage in activities that undermine public confidence of counselling profession.
- Appropriate use of counselling skills within the context of code of conduct
- Maintain high standards of professional conduct
- Must not attend to clients when her functioning is impaired due to emotional disturbances, illness, drug or alcohol intoxication, or for any other adverse reason.
- Must bring to the attention of appropriate authorities the misconduct of another counsellor
- Must inform clients about the nature of counselling offered and contractual obligations, e.g. timing, duration, confidentiality and boundaries (counselling contract)
- Must keep all information about the client (including his/her identity) confidential. This applies all clients, previous, current, children, students including those who are dead. Confidentiality may only be broken when court proceedings require so and or when a client’s details have to be discussed with another care giver for case management purposes (shared confidentiality)

**Client Safety**

- Counsellors should take all reasonable steps to ensure that the clien suffers neither physical nor psychological harm during counselling
- Counsellors do not normally give advice.
- Client safety: The wellbeing of the client must supercede all other issues. Therefore, the counsellor must ensure that client’s affairs and safety are protected at all costs.

**Client Autonomy**

- Counsellors are responsible for working in ways which promote the clients control over his/her own life, and respects the clients’ ability to make decisions and change in the light of his/her own beliefs and values
- Counsellors do not normally act on behalf of their clients unless at the express request of the client or in certain exceptional circumstances
- Counsellors are responsible for setting and monitoring boundaries between the counselling relationship and any other kind of relationship and making this explicit to the client
- Counsellors must not exploit their clients financially, sexually, emotionally or in any other way. Engaging in sexual activity with the client is unethical
- Clients should be offered privacy for counselling sessions. The client should not be observed by anyone other than their counsellors unless they give informed consent. This also applies to audio/video taping of counselling sessions
- The counsellor is just an agent of facilitator of change. This means that the client decides who he/she wants to attend to him/her and what h/she wants at the end of the sessions. The job of the counsellor ends at helping the client to make informed decisions

**Contracting**

- Counsellors are responsible for communicating the terms on which counselling is being offered, including availability, the degree of confidentiality offered, and their expectations of clients
- It is the client’s choice whether to participate in counselling. Reasonable steps should be taken in the course of the counselling relationship to ensure that the client is given an opportunity to review the terms on which counselling is being offered and the methods of counselling being used.
- If records of counselling sessions are kept, clients should be made aware of this. At the request of a client, information should be given about access to these records, their availability to other people and the degree of security with which they are kept
- Counsellors have a responsibility to establish with clients what other therapeutic or helping relationships are current. Counsellors should gain the clients permission before conferring with other professional workers.

**Competence**

The counsellor must:

- Ensure that she has received appropriate training in counselling
- Seek on-going training, supervision and consultative support. Counsellors should monitor actively the limitations of their own competence through counselling supervision or consultative support, and by seeking the views of their clients and other counsellors
- Work within her limits of competence and is an indication of competence of counsellors when they recognize their inability to counsel a client and make appropriate referrals
- Regularly monitor her counselling skills and maintain a desired level of competence.
- Counsellors should not counsel when their functioning is impaired due to personal or emotional difficulties, illness, disability, alcohol and drugs or for any other reason

**Responsibility to former clients**

- Counsellors remain accountable for relationships with former clients and must exercise caution over entering into business relationships, friendships, sexual relationship, training and other relationships likely to breach the contractual obligations. Any changes in relationship must be discussed in counselling supervision. The decision about any changes in relationship with former clients should take into account whether the issues of power dynamics present during the counselling relationship have been resolved and properly ended
- Organization and management: the counsellor is expected to operate within the confines of the rules and regulations as stipulated by the Zambia Counselling Council. The organization of the counselling space should be in accordance with what is considered appropriate. The counselling environment should be well organized as to provide ample space and privacy to the clients. Records and registers of client matters should be well kept under key and lock.

**CHAPTER EIGHT: Counselling Supervision (Professional and Emotional Support for Counsellors)**

**Definition**

A working alliance between a supervisor (experienced counsellor/trainer) and a counsellor or a group of counsellors in which the counsellors can reflect on himself/herself and on his or her work. He/she gives an account of his/her work and receives feedback from the supervisor

Objective: To maximize the competence of a the counsellor in providing a helping service to clients

Supervision is for:

- The protection of clients’ welfare
- The on-going accountability and professional development of the counsellor
- The supervisor can only “work” with what the counsellor brings
- It is essential for the counsellor to have the intention and responsibility to use supervision well and to know how to do this
- There is real need to training counsellors as supervisors
- The counsellor counsels the client
- The supervisor does not directly counsel the client

**The Supervision matrix**

**Core Supervision Process**

- Attending
- Awareness
- Exploration
- Integration
- The counselor –supervisor relationship contains all the elements of a counsellor-client relationship
- The supervisor relationship is essentially educational in nature. The supervisor is there for;

1. Challenge and

2. Support

- The supervisor helps the counsellor discover and unlock his/her own resources

**Working with the supervisor**

- The counsellor MUST have the intention and responsibility to use supervision well and to know how to do this
- The supervision can only work with the material that the counsellor brings to supervision
- Supervision sessions (individual) normally last one hour and time intervals between sessions is arranged between supervisor and counsellor
- Group supervision sessions normally lasts 1½ – 2 hours every 4 - 6 weeks
- The counsellor – supervisor relationship like that of counsellor-client relationship contains the element of confidentiality
- The Supervisory relationship is essentially educational in nature – but is also is a way of helping a counsellor develop self- awareness
- The Supervisor helps the counsellor to unlock and discover their own inner and outer resources
- The supervisor acts as a mirror/reflector for the counsellor

Types of supervision

**One to one**

Supervisor and counsellor, trained or in training

(This can also be adapted to one supervisor and two counsellors)

**Two peers trained or in training**

**This can be a group of counsellors trained or in training with a supervisor leading**

**Peer Group supervision**

A group of counsellors trained or in training rotating leadership in the group. This could also be a group a group of supervisors

**Observation of a trainee counsellor in the workplace**

Please note that the supervisor is observing and not counselling, any comments that the supervisor needs to make are done after session without the client present

**CHAPTER NINE: Cultural, Traditional and Psychosocial Issues of HIV/AIDS**

The role of Culture and Traditions in the epidemiology of any disease among cannot be overemphasized. Certain cultural practices have contributed to the rate of HIV infections in Sub-Saharan Africa.

Culture is a set of customs, beliefs and mind set of a given society or ethnic entity. Culture is also a way in which a people express their values and civilization and is often expressed within the parameters of their social and ethnic boundaries.

Tradition refers to the practices that a group of people do as part of normal life such as dress code, traditional ceremonies, important observations, code of conduct and leadership etiquette.

The following cultural and traditional practices are sexual in nature and as such do have a bearing on the prevalence of HIV infection:

| **Cultural/Traditional Practice** | **Purpose/belief** | **Issues to consider** |
| --- | --- | --- |
| **Dry sex**: Removal of natural vaginal secretions before or during a sexual act using herbs, drugs or a cloth. | - To Increase friction - To please a man - To enhance a man’s performance - For maximum pleasure | - Causes irritation or inflammation - Enhances HIV transmission |
| **Sperm bath**: Smearing semen on the baby on the first sexual encounter (act) after delivery | To strengthen the baby and protect from communicable diseases | - Consider infected parent(s) - Tender skin of child - Micro cuts Discordant couples |
| **Marital shave**: The removal of pubic hair from each other by married persons (usually followed by a sexual act). | - Mutual hygiene - To enhance trust/faithfulness - Monitoring the reproductive health of spouse (STIs???) - Stimulant for coitus | - Discordant couples - Micro cuts (minor bleeding) Secretions (vaginal/semen) Re-infections The use of condoms??? |
| **Post coitus wiping**: Some women smear semen on their thighs after every sexual act. | - To demonstrate affection for the man. - To strengthen the marriage bond - Semen is believed to increase the woman’s skin smoothness. | Discordant couples  Micro cuts |
| **Sexual cleansing**: A customary practice that forces a widowed person to have sexual relations with the dead spouse’s sibling. | To make the widowed spouse feel (psychologically) free from the dead partner’s spirit. | The deceased may have died of AIDS or related condition |
| **Tattoos**: Minute cuts on the skin made deliberately with a sharp instrument e.g. razor which may be shared among close family members. Some do suck blood or even rub tattoos with each other  **Beauty tattoos**: Tiny scars made on the woman’s forehead, thighs, belly and around the waist | - Medicinal - Family bonding or budding - Ethnic identification   Beauty, Identification  Medicinal (fertility), Stimulant for coitus, To enhance coitus (when man touches/feels the scars) | Shared instruments  Blood contact  Infected family member  Shared instruments  Blood contact  Infected family member |
| **Breast milk related**: Apart from breastfeeding, women squeeze breast milk onto a baby’s genitalia and into the eyes  **Wet nursing**: A breastfeeding mother breastfeeding a dead woman’s baby | - To clean the urethral meatus - To “sweeten” the sex organs - To treat eye infections - To neutralize poison - Antidote for spitting cobra’s vernom - Nutrition   The child’s dead mother is usually related to the woman who is breastfeeding | Consider an infected woman  Consider an infected woman/child |
| **Initiation ceremonies**: that include circumcision (male and female)  Enlargement of penis and elongation of labias using herbs or pulling | Traditional education  to prepare adolescents for adulthood responsibilities including sex | - Shared instruments - Proof of manhood/maturity - Hyper-sexuality and or cross infection - STIs - Reported sexual supremacy or satisfaction |
| *sexual intercourse with a child or an old woman | - To get rid of (cure) HIV infection - Purification to chase evil spirits - To enhance business prowess | Infected perpetrator  Child abuse  Rape |
| **The wife assistant**: A young sister or cousin is appointed, with blessings/consent from the parents and the current wife, to become the second wife and assist the elder one with house chores and other marital responsibilities | - To relieve pressure from the aging woman - To maintain the man within the family - To have more children from the same man - To retain wealth within the family | Increased HIV incidence rate where on is infected |
| **Cutting fire wood**: Traditionally done by the men or husband’s male relative in his absence. Fire wood keeps the family warm. | - To provide family support - To sustain entertainment within the family - To provide protection against outsiders | Risk of extra-marital sex by migrant worker’s spouse |

NOTE: This compilation is based on experience sharing among trainee HIV and AIDS counsellors, during courses conducted by the Counselling Services and Training Unit of the Ministry of health, around the country and beyond the borders of Zambia, over a period of twenty (20) years.

Psychosocial issues

Psychosocial issues are those concerns which affect the clients’ ability to cope with life’s events effectively. These are feelings and thoughts about the situations in which the clients find themselves particularly the negative happenings that are associated with one’s HIV and health status. What the clients experience their minds (psycho) whenever a seemingly bad situation unfolds are negative emotions and feelings such worries about one’s health, fear of death, fear of rejection, stigma and discrimination, embarrassment, loss of love and suicidal feelings.

On the other hand, relationships with people in the community (social), is the clients’ other area of life that suffers setbacks whenever a bad situation happens. Many clients may think that they would be rejected, divorced, or dismissed from employment if they disclosed their HIV status to others. An HIV positive mother may think that people will know her HIV status if she were not seen breastfeeding her baby and she may therefore fear nursing her baby close to other people, lest they ask why she is not breastfeeding her baby.

**CHAPTER TEN: Counselling Skills**

Skills are abilities or competencies used by individuals to deal effectively with everyday demands and challenges. Counselling skills can be compared to a tool bag which contains an assortment of tools and gadgets. Depending on the fault, the mechanic decides which specific tool should be used to fix the problem. In the same way, a counsellor uses different skills and techniques to use during problem-solving.

**Attending skills:** Attending means more than giving someone attention. It also means paying attention to someone’s feelings, expressing awareness and interest in what client is communicating verbally and non-verbally. This shows that the counsellor is interested in the welfare of the client and makes him (client) to feel cared for. In a nut shell, attending to a client requires that the counsellor practices the following actions:

1. Receiving the client in a warm and friendly manner
2. Giving client the attention that s/he deserves
3. Making the client to feel accepted
4. Making client to feel comfortable
5. Listening to the client
6. Establishing a therapeutic relationship with client
7. Treating the client well
8. Giving client enough time to talk about himself

Attending also includes the following attributes: Friendliness, Courtesy, Eye contact, Relaxed body posture, Minding the body language and listening to voice tone and speech rate

SUB-SKILLS OF ATTENDING (SOLER)

- Sit squarely (ready to work)
- Open posture (show good attitude)
- Lean toward the client (listening type)
- Eye contact (be observant)
- Relaxed (no need to panic)

**Body language**

- Effective Counselors are mindful of the cues and messages that are expressed by their bodies as they interact with clients.
- Your verbal and nonverbal behavior should clearly indicate your willingness to work with the client.

**Emotional presence**

- Not just being physically available but:
- Giving your full attention to the client
- Being alive to the client’s immense emotional needs, moment by moment

# Listening skills: Listening refers to the ability of counselors to capture and understand the messages clients communicate as they tell their stories, whether these messages are transmitted verbally or non-verbally clearly or vaguely (Egan, 1998, p62). Clients want more than the presence of the counselor; they want the counselor to be present psychologically, socially and emotionally.

**Purpose of Listening**

- The purpose of listening is to understand:-
- The person better
- His message
- His feeling
- His problem situation
- His deficiencies
- His strengths

**Basic Levels of Listening**

- The Clients’ Verbal Communication
- Actual words (content of what is said)
- Factual information (facts of the story)
- Underlying messages (explicit and implicit meanings)
- Feelings and moods (expressed positively or negatively)
- Distortions and gaps (things avoided, omitted or misrepresented)

**The Clients’ Non-verbal Behavior**

- Bodily behavior e.g. body movements, posture and gestures.
- Facial expression e.g. twisted lips, frowns, twinkles, smile.
- Voice tone e.g. pitch, voice level and intensity, pauses, fluency.
- General appearance e.g. type of dress, walking mannerisms.
- Non-verbal cues and messages are interpreted differently in different cultures; and it is important for the counselor to develop a working knowledge of the meaning of non-verbal behavior in the environment in which she works (Ivey et al 1993)

# Listening to Oneself as a Counselor

# This in tells the counselors own understanding of feelings towards the client and aspects of this story. Very often people have a tendency of blaming or shifting blame to others. E.g. some clients with neurotic disorders may project feelings of a significant other person onto the counselor, thereby creating problems of how to progress/proceed. An effective counselor should be neutral, appreciative and sensitive to the needs and aspirations of her client while, at the same time, recognizing and addressing those feelings in a more constructive way.

**Basic Listening Sequence** (Alta van Dyk, 2001) is intended to bring out the clients story with minimum instructions on the part of the counselor. The sequence involves the use of the following techniques: Questioning, Paraphrasing, Challenging, Reflection of feelings and Summarization.

**Types of Listening**

- Pretending to Listen
- Partial Listening
- Selective Listening
- Preconditioned Listening
- Evaluation Listening
- Filtered Listening
- Sympathetic Listening
- Total Listening

**Reasons for Inadequate Listening**

- Attraction and transferences
- Physical condition of the counselor
- Unresolved issues and other pressing concerns.
- Over eagerness
- Similarity of problems
- Differences in opinion

**Joining:** Joining is the action of getting to know the client and making him/her to trust you to lead them through an intervention. Joining requires that you learn the client’s language and work at their level of doing things. It is essentially a relationship building technique that brings out the real person in your client. It is difficult to work with special clients such as children, the elderly, drug abusers or battered women if the counsellor fails to join (connect) with them

**Paraphrasing skills:** A paraphrase is a statement that is interchangeable with that of the client. It is quite similar with rephrasing. To Rephrase is to put it in another way, Reshape it, Rearticulate it, **Say it differently AND Change the meaning altogether** but to Paraphrase is to Summarize, Reword, Interpret, Translate, or to **Say it differently BUT Maintain the meaning.** Paraphrasing is repeating what the client has just said but using your own words or few of the client’s words. When paraphrasing, the counsellor tries as much as possible to interpret and think at the same level with the client:

- **“In other words** what you are saying is that you have decided to marry your neighbour’s cousin”.
- **“In short** you mean to say that you are not a graduate because you didn’t attend university”.
- **“So what you are implying** is that your girlfriend has started seeing another man”.

Uses of Paraphrasing

- Acts as a promoter and stimulator of discussion
- Is an effective way of responding
- Is a useful means of clarifying issues
- Acts as a client’s mirror during the discussion
- Provides useful hints for the client during the session

Example of a paraphrase

Client: “*Counsellor, I do not like okra. When I was 10 years old, aunt Zrai used to enter into my bedroom at night and liked to play with my ‘dudu’. She also made me to touch her ‘udu’ and held me in her arms. Sometimes she was very rough and I experienced pain on my dudu but aunt Zrai said it was alright. I didn’t like her udu because it felt oily, like okra but she forced me to play with it anyway*….”.

Counsellor: “*What I hear you say is that aunt Zrai sexually abused you when everybody else was asleep, please tell me more*”.

Exercise: paraphrase the following statements:

1. “Counsellor, my parents died when I was in grade 6. I struggled to reach grade 7 but my relatives failed to support me. I wanted to finish school but was unable. So I just decided to do business”
2. “Counsellor, I will be leaving for Japan next week with my family. I will be away for 3 years and will obtain a degree. Whilst in Japan, I will be entitled to an education allowance for my children, and paid holiday travel every year. So I have come for HIV test”.
3. “I will not go to church this Sunday. I don’t have proper shoes for the choir ceremony. I am the only one who wears torn shoes. You know it can be embarrassing wearing such tattered shoes in church”!
4. “Counsellor, Just as I arrived at the bus station, the bus was leaving. I waited for another bus in vain until I managed to board a truck. By the time I reached the offices, the interviews were over”.

**Reflective Skills**

The starting point for these set of skills is the client’s frame of reference. Capturing what the client is telling you and repeating the message in your own words. Using this skill enables you to communicate the core values to clarify and to acknowledge clients experiences. It’s a powerful skill to building relationship

Reflection of feelings is an attempt by the counsellor to paraphrase in fresh words the essential attitudes/ feelings (not so much the content) expressed by the client. The counsellor attempts to mirror the client’s attitudes for his/her better understanding and to show the client that he or she is being understood by the counsellor

**Challenging skills**

A challenge in counselling is an invitation to allow clients reflect on their self-defeating thoughts and behavioural patterns. To challenge a client means helping a client to examine thoughts and behaviours that may be harmful to self and others. In other words, challenging is helping a client to stop and think again

What should be challenged?

- Self-defeating behaviours such as intending to indulge in reckless behaviours
- Deliberate distortion of facts by the client who is fully aware of facts but wants to pretend to e ignorant
- Game playing (time wasters)
- Transferences or in a situation where the client sees ‘another person’ in the counsellor e.g. the client who likens the counsellor to a former lover
- Hesitancy to work on solutions e.g. in situations where the client has not done part of his/her assignment
- Self blame and concentration on failures (defeat)
- False identity (pretending to be another person)

What causes defeat?

- Fear of the unknown, Burnout and not being appreciated, Missed opportunities, Past experiences, Seeking recognition, Lack of courage to face reality and immature evaluations

How to challenge

- By using the skill of immediacy (The here and now)
- Use of empathy
- Cognitive restructuring (rational therapy)
- Helping clients identify their defeated behavior
- Use of reflection skill (giving time for the client to reflect on their behaviours)

Examples of challenges

- “*How is this going to help you”?*
- *“What if your spouse discovers this? “*
- *“How would you feel if your spouse did this to you? “*
- *“Don’t you think you are being unfair to others? “*
- *“What are the possible consequences of these actions? “*
- *“Apart from that, what else can you do? “*
- *“What if you are the one who is at fault? “*
- *“Have you ever considered seeking professional help? “*

**Empathy technique**

Introduction: It feels good to know that others “understand” the way you feel. Equally, clients feel good whenever we show that we understand their feelings. A good counsellor is one who acknowledges his/her client’s feelings. Showing that you’ve understood clients’ feelings strengthens the counselling relationship.

Empathy and sympathy: Empathy is a way of showing clients that you have an idea about how the situation affected your client.

- Sympathy is FEELING FOR (sorry or pity) the clients
- Empathy means FEELING WITH the client. It means Imagining the client’s problem and understanding its impact on the his/her life

**True empathy** entails “Entering the private perceptual world of another person and becoming thoroughly at home in it. It involves being sensitive, moment by moment, to the changing felt meanings which flow in this other person, to the fear or rage or tenderness or confusion or whatever he/she is experiencing.

- It also means temporarily living in the other person’s life, moving about it delicately without making judgements” (Egan, 1994)
- “Trying to live in the other person’s life” (Kwapa, 1996)
- “Wearing another person’s shoes without putting them on” (Nsalamo, 2008).

Primary empathy

1. **Listen** attentively to the client’s story.
2. Imagine the **feelings** contained in the story.
3. **Communicate** this understanding back to the client

Advanced empathy

1. **Listen** attentively to the client’s story.
2. Imagine what **feelings** are contained in the story.
3. **Communicate** this understanding back to the client
4. **Give a reason** for this understanding.

**Advantages of empathy**

- Empathy strengthens the therapeutic relationship.
- It shows that the counsellor interested in the welfare of the client
- Helps clients to confront and be in control of their own feelings
- Provides a means of assisting clients to deal with negative feelings
- Builds client’s confidence in the counsellor

Examples of empathy

- **Client:** “*I don’t know what to do, my husband is having an affair with our maid*”
- **Counsellor:** “*That sounds weird, you must be feeling* ***disappointed*** *and* ***upset***”
- **Client:** “*These days I don’t experience nightmares*”
- **Counsellor:** “*I am sure you are now* ***enjoying*** *your sleep*”
- **Client:** “*I have just been promoted as sales manager*”
- **Counsellor:** “*That sounds great. You must be feeling* ***excited*** *and* ***on top of the world***”
- **Client:** “*Counsellor, I am pregnant and am expecting twins*”
- **Counsellor:** “*You will soon be a mum, you must be* ***overjoyed***”
- **Client:** “*It is very hot these days. I tend to bleed when it is like this*”
- **Counsellor:** “*I understand, you must be feeling very* ***uncomfortable***”
- **Client:** “*Whenever I think of him, I feel so good!*”!
- **Counsellor:** “*That must be an* ***exciting*** *feeling, I’m sure you are* ***in love***”

**Probing Skills**

This is the basic tool used to explore, clarify and discover information about the client and his concerns. Probes are verbal tactics to help clients talk about themselves and define their concerns concretely in terms of specific experiences, behaviours, and feelings. Probing also helps identify themes that may emerge when exploring these elements

Probes can help clients explore their initial concerns, examine issues more fully and explore different goals. They can encourage and prompt clients when the clients fail to take those steps spontaneously. Probing can take the form of statements, interjections or questions (often open ended questions)

***Open-ended questions:*** An open-ended question will often allow the client to say more about a subject, which needs to be explored or clarified e.g. “Can you explain how you found yourself in this situation” or “Could you elaborate more on the problem?”. Clients are given the leverage to explain issues thereby expanding their thought processes and assimilation of new information.

***Close-ended questions:*** In which clients are asked specific questions for specific and direct responses e.g. “Yes” or “No” Closed-ended questions should be used to elicit specific responses to particular pieces of information e.g. “you are not sleeping badly these days, are you?” is both closed and leading. It tells what kind of answer is expected. Closed questions are intrusive and often place the client on the defensive mode and should therefore be used occasionally

***Using statements:*** More in context of a paraphrase instead of asking either open or closed-ended questions. For instance the question “you are not sleeping badly these days, tell me more” or “You have just been promoted, tell me how you feel “

***Using interjections:*** Primarily intended to focus the client’s trend of thought or discussion to a particular theme e.g. “Before you talk about the lions, can you explain what you meant by saying that are a real guy”. In practice, interjections should be used only occasionally since they tend to be intrusive

**Using prompts:** Prompts are used to encourage the client to continue narrating his story without necessarily interrupting the flow of thought. There are verbal prompts e.g. “huh, mmm, I see” and non-verbal prompts such as the nodding of one’s head to encourage the client to continue talking.

Probing as a communication skill

- Use open-ended questions mostly
- Use close-ended questions only occasionally
- Use statements to paraphrase
- Use interjections to clarify themes
- Use prompts to encourage the client talk

**Empty chair technique:** Using the empty chair technique, the counsellor can help the client to talk to an empty chair to help him/her practice how s/he will approach and talk to the real person when they happen to meet. The empty chair technique helps the client to gather courage and feel more confident when approaching the real person

**Enactment:** Demonstrating or role-playing a situation in the presence of the counsellor also enables the client to reproduce the situation in the counselling room. Enactment is particularly useful in child counselling environment when a child is allowed to play or imitate a situation.

**Reframing:** Reframing is simply renaming a negative situation to enable the client to view the situation differently. When reframing, the counsellor helps the client to think of a euphemism or user-friendly name or description of the situation to give it a human face. This way, the client may feel less troubled talking about negative of frightening concepts e.g.:

The problem of alcoholism may be reframed as “serious drinking”

- Mental patient may be reframed as “user of mental health services”
- Bedwetting may be reframed as “Paraffin episode”
- Defeacating is reframed as “Poopoo”
- Prostitution is reframed as “Sex work”

**Praise approximation:** Giving praises even for small achievements helps clients to feel important. The counsellor should have the eyes of the eagle and be able to bring out good attributes that the clients are not seeing in their selves. Praise approximations motivate clients to want to feel praised many more times. Examples of using praise approximations are:

*Client*: “*I think am not making any progress, I keep relapsing”*

*Counsellor*: *“Well JJ, you are making steady progress, at least you never miss your appointment.”*

*Client*: *“Oh my God, this won’t work. This girl will not accept to marry me.”*

*Counsellor*: *“Maybe it won’t work JJ, but you did manage to take her to dinner yesterday and you have also convinced her to give you her photo”*

**Use of silence:** Sometimes clients remain silent for many seconds or minutes and this may prompt inexperienced counsellors to want to say something in order to fill the silence with words! A good counsellor should learn to feel confortable with silence because sometimes it is therapeutic to remain silent.

Silence may help the client to rewind and refocus on important elements of the session with the counsellor. However, the client should be the to signal to the counsellor that they are ready for the discussion to resume, will do so by sighing deeply, shifting in the chair, casting an affirmative look at the counsellor or merely commenting on the process.

**The counselling environment**

The Counselling Room should be well lit, well ventilated and clean room with at least three chairs plus a table in the corner. There should also be enough IEC materials (educational magazines, pamphlets, brochures, posters)

**Privacy**: Counselling should ideally be provided in a well secured room away from the noise and eyes of others to ensure client’s privacy.

- Confidentiality: Counsellor should keep all Information about the client to himself!
- Contract: Verbal or written agreements to protect both the client and the counselor. The contract should spell out the number of sessions and time frame for each session including the dos and donts for both parties.
- Dressing: The counsellor should always be mindful of the messages that their dressing may send to their clients. Modest or smart casual type of dress is permitted for all practising counselling counsellors.
- Respect: Clients are the reason why counsellors are called counsellors, therefore there is every reason to accord them the respect that they deserve.

**CHAPTER ELEVEN: Counselling Special Groups**

Special groups refer to those persons in the population who are more likely to experience psychosocial problems such as abuse, rejection, abandonment, discrimination, illiteracy, poverty and disease. Special groups include women, children, street kids, orphans, drug addicts, prisoners, users of mental health services, sex workers, elderly people, unemployed persons and couples.

**Couple counselling**

Couple counselling is important because most of the new infections occur among people who are in a long term relationship because they do not realize that they are at risk. Additionally there are very few care and support programs that target couples. The available psychosocial interventions do not fully address the various needs of couples as a special population group.

Couple counselling refers to the therapeutic dialogue in which a skilled counsellor helps a couple to resolve issues that affect their relationship through dialogue. If couples are helped, they in turn can provide guidance to their own family members.

Couple means any of the following:

- Pair
- Two people
- Husband and wife
- Boyfriend and girlfriend
- Two people in a long-term relationship (heterosexual or homosexual)

**Why Couple Counselling?**

- There are few prevention programs for couples; and those available do not fully targeted couples as a special population group.
- Couple counselling provides a forum at which the spouses can make negotiations for safe sex.
- Couple counseling helps to strengthen marriages and family relationships.

**Common Reasons Why Couples Seek Counselling**

- Ill health due to HIV/AIDS and other health problems.
- Reproductive health issues e.g. Infertility, family planning, STIs
- Relationship issues e.g. loss of interest in partner, Unfaithfulness & lack of trust, communication problems, Alcohol and drugs,
- Sexual problems e.g. impotence, frigidity or lack of arousal, painful intercourse e.t.c.

**Causes of Conflicts in Couples/Relationships:** Psychologists have listed 10 areas of symptom formation in couples:

1. ***Economic issues***: This can be a source of conflict in a home because it paves way for insecurity and suspicion if one partner is not financially accountable to the other.

2. ***Companionship-intimacy***: Newly married couples always experience superb feelings of intimacy and closeness. These initial feelings naturally wane off as the partners pursue other interests. This may generate feelings of abandonment in each or both partners.

3***. Work and recreation***: Work and recreation have got their own share of demands on the time of the newly married. If partners do not adjust their time and plan accordingly, this may create a huge emotional drain on their relationship.

4. ***Parenting***: The arrival of children requires that spouses apportion some of their time toward the raising of children. Some partners are often caught off guard by this development and may have problems dealing with the “new arrivals”.

5***. Family involvement***: Newly married couples do experience problems dealing with relatives who have different needs. In a situation where a spouse values the relatives more than his/her spouse, it may not be long before conflicts begin.

6. ***Religion***: The influence of religion on one’s marriage is not very different from the influence of the extended family. Many individuals with strong religious values make quick decisions to marry without considering marriage obligations. This creates confusion in home marriages. It is often said that partners who attend the same church groups have a more cohesive relationship than those who attend different church groups.

7. ***Friends***: Friends can have profound influence on a relationship depending on how close they are.

Some spouses maintain a circle of friends from the work place, church or neighborhood for various reasons. Different friends bring different experiences and challenges in our lives. We learn a lot of things from our friends such as how to please or displease ourselves!

8. ***Substance abuse***: Alcohol abuse has been documented to be one of the leading causes of social, emotional, economic and physical maladies in humans. Abuse of alcohol and hard drugs has been cited as precursor to relationship problems including divorce. Heavy alcohol consumption has been known to reduce a man’s sexual potency leading to impotency including the birth of low weight babies.

9. ***Communication***: The commonest relationship problem that is usually cited nowadays is lack of adequate communication between partners. Effective communication is one that goes beyond talking, that is to say, empathizing with one another and sharing innermost feelings.

10. ***Household chores***: It is generally believed that the woman’s place is in the kitchen. A working woman will appreciate a man’s help in the kitchen because that gives her ample time to look after the kids and at the same time be able to provide the needs of the husband. This is usually another common area of conflict especially in a situation where both husband and wife work.

**Characteristics of Marital Conflicts**

- Polarization: Point at which the two spouses begin to be emotionally detached from each other.
- Faulty interpretations and or perception of events (suspicions)
- Relationship deteriorates from warm to cold (no affection)
- Changes in communication pattern i.e. from openness to withdrawal and characterized by hideous and sometimes secretive behaviours e.g. hiding pay slips, unilateral transactions e.t.c.)

**Signs of an Ailing Relationship**

- Lack of proper communication
- Infidelity (unfaithfulness)
- Alcohol/substance abuse
- Lack of respect or regard for the other
- Stealing from each other
- Neglecting the other’s emotional or physical needs
- Fights or threats of violence
- Threats to divorce

**When dealing with a problem relationship, what is the role of couple counsellor?**

- Relationship building: To assist the couple to relate and interact with each other not only as husband and wife but also as friends
- Improve communication: Encourage spouses to talk to each other, plan together, do things such as HIV testing, together.
- Enhance sexual life: Encourage couples to enjoy sex and make informed choices on issues of safe sex.
- To help couples adjust to issues of divorce

**Couple Counselling Techniques**

***Neutrality*:** Respecting the position that each spouse has taken without taking sides.

***Reciprocity negotiation:*** Helping the spouses to develop benchmarks for achieving harmony in their relationship. This should be done in consultation with each other.

***Decentering*:** Making the spouses to address each other directly instead of doing so through the counsellor as is the case in most encounters with couples.

***Causality*:** Establishing how each spouse is contributing to the problem at hand.

***Enactment*:** Making the spouses to demonstrate a problem or solution through acting

***Empty chair technique*:** Encouraging a spouse to act possible solutions by addressing an empty chair

***Hypothesizing*:** Making an assumption about possible events that have led to particular situation usually serves as a starting point in problem solving.

## Partner Notification and Disclosure

**Partner notification/disclosure**: Revealing one’s health status to a partner or significant others.

# Types of disclosure

***Voluntary***:

- Free will (own accord)
- Informed decision: when one perceives benefits after being informed about the pros and cons.

### ***Involuntary***:

### Mitigation purposes

- Pre-requisite for insurance/work, marriage, sponsorship etc.
- Mandatory e.g. contact tracing
- Accidental

**Self-disclosure**: Mainly refers to counsellors’ ability to share his/her own experiences that are similar in nature to those of the client.

**Direct disclosure**: When counsellor discloses own personal experiences

**Indirect disclosure**: Disclosing another person’s experiences

# Purpose of partner notification/disclosure is mainly to promote personal responsibility in the area of HIV prevention, care and support. Emphasis is mainly on the following risk reduction plan:

- Taking personal responsibility to protect oneself from getting re-infected
- Taking personal responsibility not to infect others
- Contact tracing to ensure that one’s sexual partners are notified/treated
- Positive living: Exploiting one’s opportunities/chances despite being infected.
- Destigmatization: reducing the shame that is attached to HIV/AIDS

# Benefits of disclosure

- Peace of mind (“everyone knows already”)
- Acceptance and adjustment
- One becomes a role model to others
- One becomes more focused in terms of life priorities (future plans)
- Access to medical care and social support
- Enhancement of family bonds

# Limitations of disclosure

- One requires a lot of courage to face the challenge
- The prospect of experiencing shame, discrimination, abandonment or even rejection is real.
- There is the possibility of suffering a broken relationship
- One’s status/position in a given community set up may pass through some changes.

**Counselling Children and Adolescents**

**A child** is a young person who is still maturing socially, physically, spiritually and emotionally

- A young person aged below the age of 18 (Min. of Education)
- A young person aged below 16 (USA)

**Adolescence** is a transition period between childhood to adulthood. It is a period during which child learns some responsibilities that go with adulthood

**Puberty** is a period characterized by development of sexual characteristics e.g. attainment of menarche, experiencing wet dreams, developing interest in the opposite sex e.t.c.

Stages of adolescence

- Early adolescence: 9 -12 years
- Mid adolescence: 13 – 15 years
- Late adolescence: 16 – 19 years

**Rights of children:** Right to life, Right to education, Right to shelter, Right to good health, Right to food, Right to a name, Right to freedom

**Child development (physical):** Increase in flow of hormones (testosterone, estrogen and progesterone), Increase in height, Development of muscles, breasts, hips, penis, Development of pimples, Deepening of voice, Wet dreams, Menarche (onset of menses)

**Child development (emotional):** Development of amorous feelings towards opposite sex, Yearning for independence, Aversive to restrictions, Increasing sense of responsibility, Increased sense of social interaction

**Common problems affecting children** include hormonal changes, lack of parental guidance, peer pressure, unrealistic social restrictions, HIV and AIDS, drunkenness by parents, guardians, lack of trust by authorities, pathological anger by parents

**Factors that affect parenting** include Poor self-esteem, Ignorance (Lack of parenting skills), Parental Mental ill health, Poverty (lack of necessary resources), Inherited aggression tendencies, Low intelligence, Negative religious beliefs

**Important points to consider when counselling children and adolescents**

- They are sensitive to strangers
- They like experimenting
- They are usually genuine/honest
- They must enjoy human rights like adults
- They are eager to learn
- They are easily influenced by peers
- Create a child friendly environment
- Use a language the child understands
- Arrange sitting to be at the same level e.g. a carpet or low chairs
- Use pictures/drawings to stimulate the child whenever necessary
- Use of **media**: Play is a child’s natural language, use play/games/story telling to draw information from a child

**Child counselling principles and strategies:**

***Use of Media*** such as play, art, games, pictures, toys, drawings, storytelling and songs. ***Play creates an opportunity for the child to join with us in a therapeutic process.***

***Setting goals***: Setting clear counseling goals help to focus the session.

Who should set the goals? - The child’s parents/guardians

- The counselor

- The child

***Proper joining***: Joining clarifies the reason for bringing the child to counseling in the presence of the child. If child has difficulty to separate from parents, both can be invited to the counseling room. At this time, child can explore while counselor talks with parents. Parents can be invited to play with the child in the play therapy room until the child feels safe and comfortable, trusts their surrounding and the counselor. Counselors need to lay down guidelines and rules in the very beginning about what is permissible and what is not.

***Child -counselor relationship*** is an important Link between the child’s world & counselor

- Exclusive – There should be good rapport involving a trustworthy
- Safe – The counselling relationship should be permissible, non-damaging and respectful of the child’s rights
- Authentic – The counselor does not pretend to be someone else
- Non-intrusive – Avoid intrusive questioning/probing.
- Purposeful – the ‘reason and goal’ for counseling should be made clear to all concerned.

***Confidentiality*** is considered the backbone of counselling and is essential for building child’s trust. It creates a safe environment for openness and should be maintained except if there is potential harm to the child or others. Parents/guardian should be made aware of confidentiality requirements and informed of the counseling progress.

**Fundamental goals for counselling children**

Counseling should enable the child to:

- deal with painful emotional issues
- achieve a therapeutic level of being themselves (congruence)
- express thoughts, emotions, and behaviors
- feel good about themselves
- accept their limitations and strengths
- change behavior – to minimize negative consequences
- function comfortably
- adapt to the external environment e.g. home, school, families etc.

**Child counselling skills/techniques**

- Observation: general appearance, moods, play, interaction with the counsellor, responses, attention span,
- Active Listening: ***Matching body language*** e.g., if child is sitting on floor, counselor to sit on floor as well, be present and available. ***Use of minimal responses -*** nodding, using minimal responses such as “ah, ha”, “Uh hm”, “yes”, “ok”, “right”, etc. Longer responses might be like “I hear what you say”, or “I understand”, or “Tell me more.” **Reflecting or Paraphrasing -** The most effective way to give the child this assurance is by using the skill called “reflection” e.g. *“It sounds like your uncle and auntie are not around very much for you” Or “You sound scared”.* **Summarizing-** A summary from the counselor draws together the key issues, that the child has been talking about. It is an opportunity to make sure you have heard things correctly.
- Helping the child to *tell the story: Ask the child to discuss non-threatening subjects (songs, likes, school, friends, home, etc.). Offer drawing materials and other media. Offer your own story to help the child to start. Ask the child, “Do you have any stories to share?”*
- Dealing with resistance and self-destructive behavior: Resistance is a way of protection /coping with stressful situations. Through therapy, child unlearns self-destructive behaviors / replaces them with productive ones. Through counseling, a child can explore productive alternatives.
- Facilitating change: Identify past experiences and behaviors which had negative consequences. Help the child to explore and weigh options, advantages/disadvantages of available choices. Weighs risks, gains, losses, costs, and also consequences, involved in making changes. Help the child to rehearse and experiment change through therapy.
- Managing termination: The decision of ‘when to terminate’ can sometimes be challenging for a counselor.

**Termination and/or referral can be considered when**:

- The child has reached his/her goal e.g. when behavior has changed, reported by parents/school. The child is happily engaged in social activities.

- The child is not moving, blocked, unable to let go of resistance.

- Focus of counseling is shifting, child continues to play rather than get involved in therapeutic work.

**Use of drawings:** Drawing picture of Reality-through drawing children can express their feelings and experiences that are hard for them to talk about. Do not influence what to draw. Suggest whatever they want to draw. Later you could suggest topics like ‘draw yourself’, or ‘draw your family’ or ‘draw a house and a tree’. When the child has drawn the picture ask him/her to tell you about it without you pressurizing him to do so. Don’t impose your interpretations on him. Drawing may be a relief in itself or it may open the way for communicating in words. Value what the child has drawn by keeping it safe.

**Use of Play technique:** Five years old boy coming from the visit to the clinic comes home and tries to prick the doll’s arm with the pen. He is conveying his experience through play. The child often shows us rather than tells us about their life. Each child has a unique way of handling and playing with human figures. Child’s play expresses something he/she has either seen or experienced. If we allow the child to choose the toy and let him play with it as he wants to, our observation can lead us to understanding that child

**Use of songs and drama:** Children can also show us their experience in a drama or a song. The choice of a song may also reveal the child’s mood.

**Counselling for Alcohol and Drug Addiction**

The aim of this topic is to increase knowledge of the biology of drug addiction, principles of treatment, and basic counselling strategies

The objectives are to achieve the following:

- Understand the reasons why people start drug use
- Identify 3 main defining properties of drug addiction
- Identify 3 important concepts of drug addiction
- Understand characteristics and effects of major classes of psychoactive substances
- Identify the treatment modalities for drug addiction

**Introduction to Psychoactive Drugs**

What are psychoactive drugs?

- Psychoactive drugs interact with the central nervous system (CNS) affecting:
- mental processes and behaviour
- perceptions of reality
- level of alertness, response time, and perception of the world

**Why do people initiate drug use?** Much, if not most, drug use is motivated (at least initially) by the pursuit of pleasure.

**Key Motivators & Conditioning Factors**

- Forget (stress / pain amelioration)
- Functional (purposeful)
- Fun (pleasure)
- Psychiatric disorders
- Social / educational disadvantages

Also, initiation also starts through:

- Experimental use
- Peer pressure

After repeated drug use, “deciding” to use drugs is no longer voluntary because **DRUGS ALTER THE BIOCHEMICAL FUNCTIONING OF THE BRAIN**

**What is Drug Addiction?**

Drug addiction is a complex illness characterised by compulsive, and at times, uncontrollable drug craving, seeking, and use that persist even in the face of extremely negative consequences.

Characteristics of drug addiction

- Compulsive behaviour
- Behaviour is reinforcing (rewarding or pleasurable)
- Loss of control in limiting intake

Important terminology

**Psychological craving:** Psychological craving is a strong desire or urge to use drugs. Cravings are most apparent during drug withdrawal.

**Tolerance:** Tolerance is a state in which a person no longer responds to a drug as they did before, and a higher dose is required to achieve the same effect.

**Withdrawal:** The following symptoms may occur when drug use is reduced or discontinued: Tremors, chills, Cramps, Emotional problems, Cognitive and attention deficits, Hallucinations, Convulsions and even Death

**Classifying psychoactive drugs**

| **Depressants** | **Stimulants** | **Hallucinogens** |
| --- | --- | --- |
| Alcohol | Amphetamines | LSD, DMT |
| Benzodiazepines | Methamphetamine | Mescaline |
| Opioids | Cocaine | PCP |
| Solvents | Nicotine | Ketamine |
| Barbiturates | Khat | Cannabis (high doses) |
| Cannabis (low doses) | Caffeine | Magic mushrooms |
|  | MDMA | MDMA |

*(Adapted from UNODC Treatnet II Training Package)*

**Long-term effects of drug use**

1. Relationship problems
2. Financial difficulties
3. Sexual difficulties
4. Unemployment
5. Mental health and behavior problems
6. Premature aging
7. Cardiovascular health problems
8. Harm to a fetus during pregnancy/spontaneous abortions
9. Premature Death
10. Increased risk for cancer, especially lung, head, and neck
11. Respiratory tract illnesses/infections
12. Immune system dysfunction
13. Seizures
14. Skin problems

**Addiction = Brain Disease:** Addiction is a brain disease that is chronic and relapsing in nature. After repeated drug use, “deciding” to use drugs is no longer voluntary because dependency sets in. Why is comprehensive addiction treatment needed?

1. Addicted individuals usually suffer from mental health, occupational, health, or social problems that make their addictive disorder difficult to treat
2. For most people, treatment is a long-term process that involves multiple interventions and attempts at abstinence

***Treatment duration:*** Individuals progress through drug addiction treatment at various speeds, so there is no predetermined length of treatment. In general, longer treatment duration results in better outcomes.

***Treatment compliance***: Client factors that affect treatment compliance are

- Readiness to change drug-using behaviour
- Degree of support from family and friends
- Pressure to stay in treatment from the criminal justice system, child protection services, an employer, or family members

***Factors within the program that affect treatment compliance are***

- A positive therapeutic relationship between the counsellor and client
- A clear treatment plan, which allows the client to know what to expect during treatment
- Medical, psychiatric, and social services
- Medication available when appropriate
- Transition to continuing care or “aftercare”

***Drug addiction treatment:*** Drug addiction treatment is offered in specialized facilities and mental health clinics by a variety of professionals such as: Medical doctors, Psychiatrists, Clinical officers, Psychologists, Social workers, Nurses, Case managers, Certified drug abuse counsellors and other substance abuse professionals

***Principles of effective treatment***

1. NO single treatment is APPROPRIATE FOR ALL
2. Treatment needs to be READILY AVAILABLE
3. Effective treatment attends to MULTIPLE NEEDS, not just to drug use problems
4. The treatment plan must be ASSESSED CONTINUALLY and MODIFIED AS NECESSARY to insure that it meets the client’s changing needs
5. Remaining in treatment for an ADEQUATE PERIOD OF TIME is critical for treatment effectiveness
6. Counselling (individual and/or group) and other behavioural therapies are CRITICAL
7. Medications are IMPORTANT elements of treatment for many clients, especially when combined with behavioural therapy
8. People with coexisting mental disorders should be treated in AN INTEGRATED way
9. Detoxification is only the FIRST STAGE of addiction treatment and by itself does little to change long-term drug use.
10. Treatment does NOT need to be voluntary to be effective
11. Possible drug use during treatment must be MONITORED continuously
12. Treatment programs should provide assessment for HIV/AIDS and other infectious diseases as well as counselling to help clients change behaviours that place themselves or others at risk of infection
13. Recovering from drug addiction can be a LONG-TERM PROCESS and frequently requires multiple episodes of treatment

**Professional Burn Out**

Burnout is emotional exhaustion which is characterized by mental fatigue and depression (depersonalisation). Symptoms are work related and it happens to employees at the work place. Burnout decreases effectiveness and work performance and reduces personal accomplishment in one’s work

“*A burned out person is someone who has invested enormous amounts of energy into making life meaningful for himself and others around him. But unfortunately s/he is running on empty. S/he is working more and enjoying it less. Life and the work place becomes a source of inner stress and turmoil* (Ponder, 1983).

Ponder (1983) further observes that burned-out persons are usually perfectionists who enter their professions with great hopes of changing the world. After a while they realize that their environment is more resistant to change than they had dreamed. Burnout is an emotional disorder. It is a form of stress.

Burnout is an indication of the need for supervision. A counsellor should be able to recognize when he/she is not able to cope with the demands of life and seek supervision from her/his supervisor.

Cause of burnout

The factors that cause burn out usually are those that prevent people from achieving their goals and expectations. High levels of commitment to one’s work without being recognized are also a prerequisite for burn out. Additionally, doing the same type of work over and over again (monotony) causes burnout.

1. Lack of work motivation (recognition, money, involvement, promotions, transport).
2. Unclear job description
3. Lack of work job satisfaction
4. Exposure to emotionally involving and demanding work situations over a long time.
5. Negative relationships with colleagues and supervisors
6. An initial state of high involvement and motivation in one’s work

Other causes

- 1. *Stressful tasks*
- Talking to clients about life threatening illnesses (depressing issues
- Talking about sensitive sexual issues
- Helping young people to face disfigurement and death
- Inadequate skills to counsel clients and their families
- Not being able to reassure clients about their condition

2. *Organisational difficulties*

- Inadequate resources to meet clients’ needs
- Lack of acknowledgement as a counsellor
- Pressure to provide other health services than counselling
- Lack of supervision or suitable supervisor
- Long working hours
- Shortage of counsellors

3.  *Personal issues*

- Anxiety regarding being infected with HIV by the client
- Unresolved personal conflicts beyond counselling work
- Over identification with client
- Giving a great deal of one’s own emotions and personal energy to others whilst receiving very little back

Symptoms of burnout

Description of burnout symptoms have been offered by many authors. Here below follows a summary of reported physical, behavioural and cognitive and affective symptoms of burnout (Miller, 1991):

- Physical exhaustion
- Emotional dumbness,
- Frequent headaches and backaches
- Emotional hypersensitivity and
- Over- pain identification
- Sleeplessness
- Grief and sadness
- Gastrointestinal disturbances
- Pessimism, helplessness, hopelessness
- Chronic, vague, physical pains
- Boredom and cynicism
- Malaise
- Depression and sense of failure
- Fatalism
- Readiness to be irritated or Frustrated
- Callousness
- Indecision
- Impulsivity, acting out
- Self-righteous heroism
- Withdrawal from colleagues
- Victimisation of patients
- Heightened irritability
- Feeling of being unappreciated

General rules for managing burnout

- Rest: Learn to knock off completely or go on leave
- Have adequate sleep
- Eat healthy food
- Be active (exercise)
- Spend quality time with your children and spouse
- Adopt more realistic expectations and more gratifying goals.
- Get a clear job description outlining your responsibilities
- Acknowledge your limitations, avoid doing what you are not qualified to do
- Avoid being a perfectionist (no one is perfect)
- Limit working hours
- Encourage departmental rotation
- Receive confidential support and supervision
- Provide opportunities for in-house- training
- Do other things, Mix career prospects to enrich your view of the world (pursue further training e.g. diploma, degree or graduate level, learn new hobbies, make new friends, learn new things, eat at a different restaurant, watch animals)
- Share or delegate some of your work (Team work)

**Management of Burn out**

1. Professional supervision and emotional support

- By external and internal facilitators
- To individual or group; regularly or on demand, can be by same or mixed professions

1. Management context

- Limit working hours
- Provide pre-work training and orientation
- Training in stress recognition and management
- Providing confidential support structures
- Enabling work variations
- Planning time away from work

1. Environment

- Expression of work stress
- Encouraging team work
- Provide appropriate work environment

# Barriers to Implementing Staff Support

# Staff may not know why they need support

# Lack of trust among colleagues

# Stigma about showing or admitting profession uncertainties

# Lack of time and support

# Role conflicts

# Counsellor should be able to identify burn out in herself or others and be able to deal with it.

**STRESS MANAGEMENT**

**Introduction to stress:** We’ve all been stressed and emotionally drained before. But if your stress seems to be going on and on, you might have a more serious condition known as **“burnout”.** When you’re burned out, you’re drained of energy to manage even the smallest problems in your life.

So how can you tell if you’re burning out? What makes it burnout? Use this short list of symptoms to see if your stress is more serious than you think. Just remember—it’s not an official depression test; it’s just a guide to point you toward answers about burning out.

Litmus test

1. Do you feel alone in the world? 2. Do you feel like not going for work? 3. Are you losing interest in your work? 4. Do you find your work overwhelming or boring? 5. Do you wonder if anything you do makes sense? 6. Does it seem like every day is a bad day? 7. Do you feel as if no one appreciates your efforts 8. Would you consider most of your life as “stressful”? 9. Do you feel that going for is just a waste of energy? 10. Are you becoming doubtful about where your life is headed?

**Scoring stress:** Any Yes answer amounts to 10% of stress in your life. If you answer Yes to more than one question, you should make an appointment to see a mental health worker because that amount of stress affects your work and relationships already.

**What is stress?**

**Stress** can be said to result from an “imbalance between demands and resources” or as occurring when “pressure exceeds one's ability to cope”. It is the conflict that results from the demands of life and one’s ability to manage these demands. Demands of life which include ***work deadlines***, ***family expectations***, ***personal ambitions*** and other ***existential situations*** all cause stress.

**Stress is a mental problem:** Stress is also called Burnout (mental exhaustion), Worry/anxiety, Post traumatic stress disorder (PTSD).

**Types of stress**

1. **Acute stress** is a short term stress and in result, does not have enough time to do the damage that long term stress causes
2. **Chronic Stress** is the exact opposite of acute stress. It has a wearing effect on people and can become a very serious health risk if it continues over a long period of time. It has been proven that chronic stress can have a huge impact on memory loss.


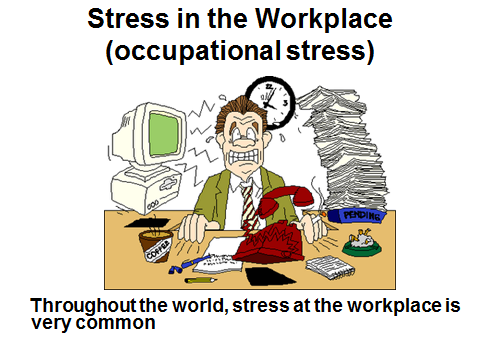


Throughout the world, stress at the workplace is very common. Managing occupational stress is vital because it improves job performance as well as relationship with co-workers and employers.

- Changing the work environment has proven to relieve work stress in some workers.
- Making the environment less competitive between employees has shown to decrease some amounts of stress.
- Recognizing that each person is talented differently is vital: some people like the pressure to perform better.

Psychologists have also found that salary predictability is equally useful. Salary cause huge amounts of stress in the workplace. Salary also affects the way a person performs because they always want promotion and in result, higher salary.

**Work place Stressors**

Stressors are factors that increase one’s stress. Among the many stressors mentioned by employees, these are the most common:

- The way my boss/supervisor treats me
- Lack of job security
- Institutional policies
- Co-workers who don't do their fair share
- Unclear expectations
- Poor communication
- Not enough control over assignments
- Relationship conflicts
- **Work place Stressors** *cont’*
- Too much work
- Long working hours
- Uncomfortable physical conditions
- Inadequate pay or benefits
- Co-workers making careless mistakes
- Dealing with rude clients
- Lack of cooperation from co-workers
- How the institution treats its workers
- Urgent deadlines

**How to deal with stress**

Since everyone has a unique response to stress, there are no “one size fits all” solutions.

No single method works for everyone or in every situation. So people should try different techniques and strategies.

Focus on what makes you feel calm and in control of the stress situation.

**Better ways to handle stress**

If your methods of coping with stress aren’t improving your emotional and physical health, it’s time to look for help. There are many healthy ways of coping with stress, but they all require change. You can either **change the situation** or **change your reaction**. When deciding which option to choose, it’s helpful to think of the four As: **Avoid**, **Alter**, **Adapt**, or **Accept**.

1. **Avoiding unnecessary stress**

- **Learn how to say “no”**– Know your limits and stick to them. Whether in your personal or professional life, refuse to accept added responsibilities.
- **Avoid people who stress you out** – If someone consistently causes stress in your life and you can’t turn the relationship around, limit the amount of time you spend with that person or end the relationship.
- **Take control of your environment** – If the evening news makes you anxious, turn the TV off. If traffic’s got you tense, take a longer but less-traveled route. If going to the market is an unpleasant chore, do your grocery shopping online.
- **Avoid hot-button topics**– If you get upset over religion or politics, then avoid the conversation.
- **Pare down your to-do list**–If you’ve got too much on your plate, distinguish between the “shoulds” and the “musts.”
- **Avoid people who stress you out**

1. **Alter the situation**

- **Express your feelings instead of bottling them up.** If something or someone is bothering you, communicate your concerns in an open and respectful way.
- **Be willing to compromise.** When you ask someone to change their behavior, be willing to do the same.
- **Be more assertive.** Don’t take a backseat in your own life. Deal with problems head on. If you’ve got an exam to study for and a friend wants to chat, do the right thing.
- **Manage your time better.** Poor time management can cause a lot of stress.
- **Don’t take a backseat in your own life – be in charge!**
- **Manage your time better**

1. **Adapt to the stressor**

- **Reframe problems.** Try to view stressful situations from a more positive perspective. Rather than fuming about a traffic jam, look at it as an opportunity to listen to your favorite radio station, or enjoy some alone time.
- **Adjust your standards.**Perfectionism is a major source of avoidable stress. Set reasonable standards for yourself and others, and learn to be okay with “good enough.”
- **Focus on the positive.**When stress is getting you down, take a moment to reflect on all the things you appreciate in your life, including your own positive qualities and gifts. This simple strategy can help you keep things in perspective.
- **Don’t try to control the uncontrollable**


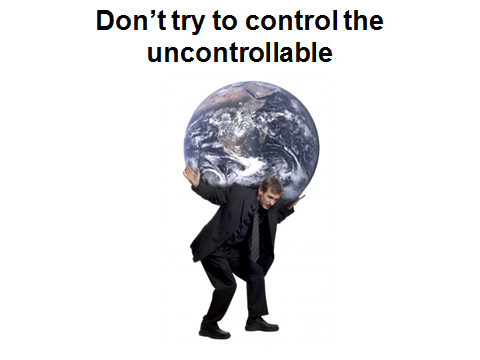


1. **Accept the things you can’t change**

- **Don’t try to control the uncontrollable.** Many things in life are beyond our control— particularly the behavior of other people. Rather than stressing out over them, focus on the things you can control such as the way you choose to react to problems.
- **Look for the upside.**When facing major challenges, try to look at them as opportunities for personal growth. If your own poor choices contributed to a stressful situation, reflect on them and learn from your mistakes.
- **Share your feelings.** Talk to a trusted friend or make an appointment with a therapist.
- **Learn to forgive.**Accept the fact that we live in an imperfect world and that people make mistakes. Let go of anger and resentments – it is negative energy.

**Balance your life**

- **Divide the day equally**


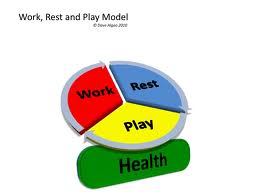

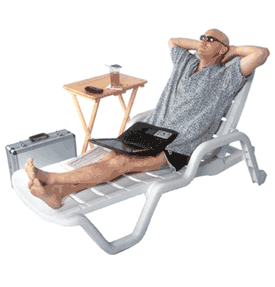
 *Set time for relaxation*

- **Connect with others.** Spend time with positive people who enhance your life. A strong support system will buffer you from the negative effects of stress.
- **Do something you enjoy every day.**Make time for leisure activities that bring you joy, whether it be stargazing, playing the piano, or working on your bike.
- **Keep your sense of humor.** This includes the ability to laugh at yourself. The act of laughing helps your body fight stress in a number of ways.
- **Set aside relaxation time:** Include rest and relaxation in your daily schedule. Don’t allow other obligations to encroach. This is your time to take a break from all responsibilities and recharge your batteries.

**Adopt a healthy lifestyle**

- [**Exercise regularly**](http://www.helpguide.org/life/exercise.htm)**.** Physical activity plays a key role in reducing and preventing the effects of stress. Make time for at least 30 minutes of exercise, three times per week.
- [**Eat a healthy diet**](http://www.helpguide.org/life/healthy_eating_diet.htm)**.** Well-nourished bodies are better prepared to cope with stress, so be mindful of what you eat.
- **Reduce caffeine and sugar.** The temporary "highs" caffeine and sugar provide often end in with a crash in mood and energy.
- **Avoid alcohol, cigarettes, and drugs.**Self-medicating with alcohol or drugs may provide an easy escape from stress, but the relief is only temporary.
- [**Get enough sleep**](http://www.helpguide.org/life/sleep_tips.htm)**.**Adequate sleep fuels your mind, as well as your body.

**Stress management techniques**

Below are some techniques that can be used to deal with life stresses:

- Engage in Social activities
- Cognitive Behaviour therapy
- Conflict resolution
- Engage in exercises
- Learn a new hobby
- Progressive relaxation
- Spend time watching nature
- Use Stress balls
- Exercise Deep breathing
- Learn Yoga Nidra or medidation
- Reading novels or any interesting book
- Set time for Prayer
- Learn relaxation techniques
- Artistic expressions, Learn Time management
- Learn Planning and decision making
- Listen to certain types of relaxing music
- Spend quality time with pets

**Unhealthy ways of coping with stress**

These coping strategies may temporarily reduce stress, but they cause more damage in the long run:

- Smoking or Using pills or drugs to relax
- Drinking too much
- Blaming or casting others to feel good
- Overeating or undereating
- Zoning out for hours in front of the TV or computer
- Withdrawing from friends, family, and activities
- Sleeping too much
- Procrastinating (postponing action)
- Filling up every minute of the day to avoid facing problems
- Taking out your stress on others (lashing out, angry outbursts, physical violence)

**Crisis Intervention**

**Concepts of a Crisis**

- A crisis is a bothersome issue/problem
- It is an emergency situation that requires immediate attention
- During a crisis, a person feels as if s/he has been knocked off balance
- A crisis is sometimes described a point of no return or a state of disequilibrium

**Characteristic of crisis:** When a crisis happens in the life of an individual, the usual coping strategies fail to work. This makes it difficult for the individual to adjust to the prevailing situation accordingly (adjustment crisis).

**Causes of Crises**

- Psychosocial causes e.g. being told that one is HIV positive, homelessness, relationship problems, economic turmoil, ”unwanted pregnancy”,
- Existential causes e.g. calamities, aging, disease, war etc

**Types of Crisis**

- Major crisis which may threaten life or peace such as coming face to face with a snake or being divorced.
- Minor crisis which is easily dealt with by many. A minor crisis sometime goes unnoticed such as forgetting one’s car keys at the office or running late to pick up the kids from school.

NB: Clients experience crises differently. They should not be expected to react the same way

**Signs of a Crisis**

- Physiological signs: Tremors, sweating, dry mouth, constipation, diarrhea e.t.c.
- Emotional signs: Confusion, inappropriate excitement, crying spells, temper tantrums, depression, silence, loss of appetite e.t.c.
- Cognitive signs: Poor memory/forgetfulness, short attention and concentration span, poverty of thought, poor judgement
- Behavioural signs: Restlessness or agitation, withdrawal from social activities,

**Aims of Counselling during a Crisis**

- To help client to gain control of the crisis situation prevent future crises
- To enable client to go through the crisis with minimum psycho trauma

**The Role of the Counsellor during a Crisis**

- Relax, remain calm (it is not your crisis)
- Be supportive through out the crisis
- Be attentive and alert to the clients immediate needs
- Encourage outpouring of emotions
- Use your problem-solving skills to help avert the crisis
- Do not force your services on a client, let client seek your help
- Discuss clients immediate concerns and deal with them promptly
- Reinforce positive reactions from client
- Help the client to plan the way forward
- Use suggestions where the client is too stressed to think clearly

**Counselling for Mental Health**

**What is mental health?**

- Mental Health is a positive sense of well-being
- It is a belief in own worth and the dignity and worth of others
- It is the ability for a person to:
  - to deal with the inner world of thinking, feeling, managing life and taking risks
  - to initiate, develop and sustain mutually satisfying personal relationships
  - to sustain a spiritual life

**What are mental health life styles?**

- Coping strategies
- Self –esteem
- Self-care
- Relationships with family members, friends, colleagues
- Utilising time, money, self
- Participation and cooperation in social clubs, religious groups, self-help groups and work

**What are mental disorders?**

- Disturbances in perception, beliefs, thought processes and mood (psychoses)
- Disturbances in mood, concentration, irritability, fatigue (neuroses or common mental disorders)
- Progressive organic disease of the brain (dementias)
- Abnormal personality traits which are handicapping to the individual and /or to others (Personality disorders)
- Excess consumption and dependency on alcohol, drugs and tobacco

**Prevalence rates for mental disorders**

- Common Mental Disorders 10-20%
- Psychoses 1%
- Personality disorders 3-5%
- Dementias 5% over 65 and 20% over 80
- Substance abuse –variable
- Childhood disorders-10%

**Symptoms of mental disorders**

- Excessive concern about bodily symptoms (headache, backache)
- Loss of enjoyment
- Low mood
- Crying
- Anxiety and panic
- Fatigue
- Poor concentration
- Impaired sleep
- Impaired appetite and weight loss
- Irritability
- Low libido
- Obsessional thoughts and actions

# Group Counselling

**Definitions:** ***A group*** is a collection of individuals who have been brought together by a common understanding or objective. ***Group dynamics*** refers to the factors that affect the formation or changes that are take place within the group from the formation to terminal stage.

**Types of group:** There are *large* and *small* groups, *closed* and *open* groups. Very large groups are usually called *crowds*.

A ***crowd*** usually forms without any common goal. Groups comprising neighbor hood misfits are sometimes referred to as *gangs*.

A ***closed group***: A group whose membership is restricted to a particular age group, social status, ethnicity, sex, religion e.g. business groups or a motor rally team

An ***open group***: Membership not restricted to any particular civil status e.g. a political party or a church.

**Characteristics of an effective group:** For counseling purposes the size of a group should be between 5 and 15. This makes it easier for the facilitator to manage the group well and be able to give adequate attention to each individual group member.

- Common goal
- Leadership
- Group roles
- Participation
- Respect
- Cohesion

**How to manage a group session**

- Organize group based on commonality of interests
- Sit in circle (be part of the group)
- Receive & greet group members
- Make self-introductions
- Introduce topic
- Discuss confidentiality with group members
- Establish group norms including suitable language
- Create responsibilities for group members including time keeper
- Play role of facilitator and not teacher
- Encourage everybody to participate and ask questions
- Be precise and stick to the topic
- Guide the discussions
- Write down important points to remember
- Be mindful of time
- Summarize contents of the group discussion
- At the end, thank all group members for their participation

# Common stages in the formation of a group: Every group passes through some stages of development before it matures as a formidable group.

- ***Forming stage***: Coming together of individuals with different backgrounds and expectations.
- ***Storming stage***: Characterized by anxieties associated with meeting strangers, viewing each other with suspicions and holding divergent views.
- ***Norming stage***: Agreeing to work together and agreeing on common goals.
- ***Performing stage***: Working together to achieve the common goals.
- ***Deforming stage***: Time to Part Company, characterized by departure tears and exchanging of phone numbers, photographs, e-mail addresses e.t.c.

**Common characters found in a group**

- **Lion**: King of the jungle and knows it all. Represents participants who think they know everything.
- **Zebra**: Best dressed animal. Represents participants whose main agenda is to out dress the others.
- **Monkey**: Typically clever. Misleads other group members but always emerges innocent.
- **Tortoise**: Very slow in every learning activity. Represents participants who always get low marks but still manage to pass.
- **Giraffe**: Long necked and long sighted. Represents the rumour mongers in the group.
- **Kalulu**: Trick star and fast animal. Usually pretends know what he does not even know. Crashes at the end.
- **Elephant**: Big, strong and reliable. Represents wise and generous group members.
- **Chameleon**: Untrust worthy. Changes color according to environment. Represents group members who always agree to anything.
- **Frog**: Enjoys making disturbing noises near water when it is dark. Represents group members who like holding some discussions while the session is going on.
- **Goat:** Confused and stubborn animal. Represents group members that appear to be confused and enjoy arguing.
- **Rhino:** The only animal that does not associate with other animals. Represents group members who do not have friends
- **Sheep:** Very obedient animal. Represents group members that always agree to everything others suggest.
- **Hippo**: Lives both in water and on land and also hates fire. Represents group members who hate change and development.
- **Grasshopper**: Enjoys hopping on lawns and tall grasses. Represents participants who hop from one workshop to another in search of sitting allowances!


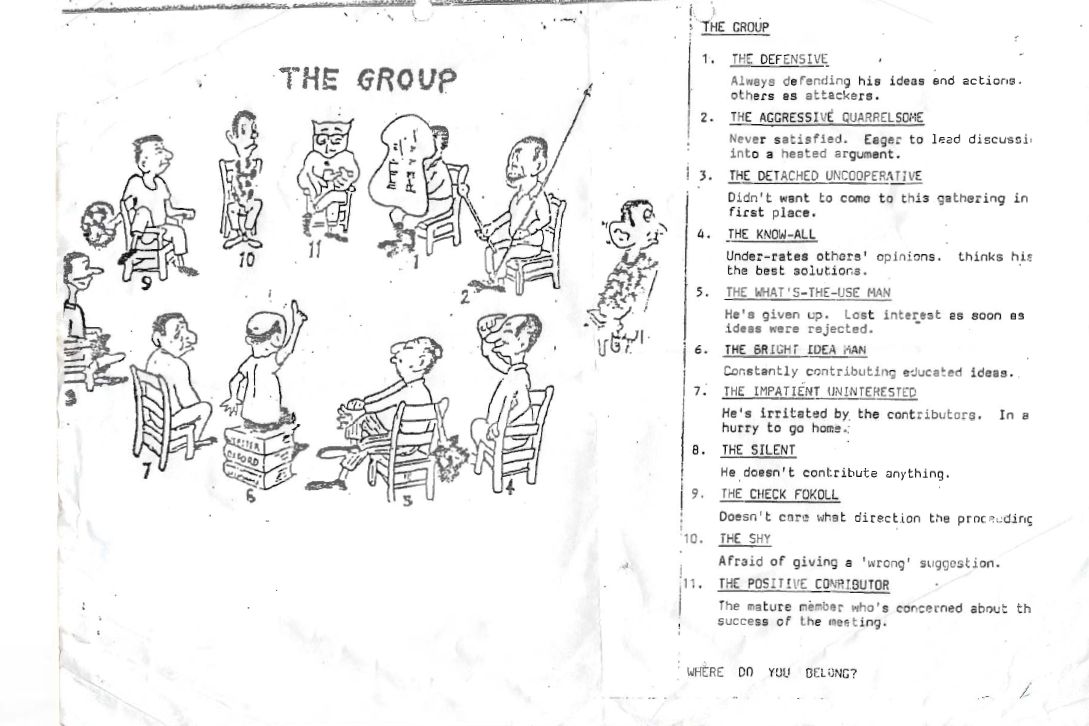


**Which Animal Character Suits Your Character?**

**CHAPTER TWELVE: Human Sexuality**

The objectives of this topic are:

- To learn about Gender and Sexual issues
- To gain knowledge on various issues that affect human sexuality
- To enhance knowledge on safe sex practices

**Rationale:** Since HIV is a sexual intercourse driven epidemic, this topic is intended to enhance knowledge on sexual issues that relate to counselling

**What is sex?** It is being male **or** female

**Sex roles** are biological functions of both males and females e.g. breastfeeding, inserting penis, giving birth, ejaculating, pregnancy, menstruation e.t.c.

**Gender:** is the general perception of men and women in society i.e. female is feminine, male is masculine

**Gender roles** are social and economic chores that both men and women are capable of performing e.g. collecting fire wood, changing nappies, cooking relish, roofing house e.t.c. Gender roles are usually interchangeable

**Sexuality** is the Maleness of femaleness of animals including humans. It includes the overall sexual behaviors and attributes of animals (both males and females)

**Sexual orientations**

- Heterosexual**: Male to female sexual attraction**
- Homosexual**: Male to male or female to female sexual attraction**
- Bisexual**: Heterosexual and homosexual at the same time**

**Common sex practices:** These include Vaginal sex, Anal sex, Oral sex (blow job), Caressing, Fondling, Giving a sexy look, Rubbing, Talking sex (hot talk), Masturbating, Looking at each other’s nakedness, Watching others in sex act, holding hands, playing house e.t.c.

**SOME BENEFITS of SEX**

### *Sex improves health and happiness*

“Sexually active people take fewer sick days, are more gregarious and enjoy life more” says Dr. Ted McIlvenna, of San Francisco’s Institute for Advanced Study of Human Sexuality. And sex guru Alex Comfort observed 20 years ago, sexually active people outlive their inactive counterpants.

### *Sex regulates your hormones*

Dr, Winnifred Cutler, director of the Athena Institute for women’s Wellness in Pennsylvania, has shown that, possibly due to prolonged exposure to male pheromones, women who have intercourse at least once a week are more likely to have normal length menstrual cycles than women who are celibate or who take a “feast or famine” approach.

*Sex boosts estrogen*

Cutler also found that women who enjoyed regular weekly intercourse had significantly higher levels of estrogen in their blood. Estrogen keeps the cardiovascular system healthy, lowers bad cholesterol, raises good cholesterol, maintains bone density, helps the skin to stay supple and prevents depression.

*Sex reduces stress*

Orgasm is a tranquilizer. During arousal, your muscles tense: during orgasm they twitch, then relax completely. This may explain the findings of the Institute for Advanced Study of Human Sexuality: People with fulfilling sex lives are less anxious, violent and hostile.

*Sex burns calories*

Vigorous sex gives you a mini work out. Dr Alfred Franger, professor of Obstetrics and Gynecology at the Medical College of Wisconsin, estimates that a 119lb woman burns 4.2 calories per minute during sex, compared to 4 calories per minute playing tennis.

*Sex boosts your immune system*

Orgasm improves immunity. Dr. Dudley Chapman, a gynecologist, monitored 24 breast cancer patients and found that those who regularly reached orgasm fared better than those who did not. Orgasm boosts infection fighting cells by up to 20 percent.

*Sex relieves menstrual cramps*

Uterine contractions during orgasm may help reduce premenstrual fluid build up in the pelvic area by forcing blood to flow back into the general circulation, relieving bloating and tightness. Cramps may also be caused by irritation of the endometrial lining by prostaglandin: orgasm may help shed this lining lowering prostaglandin levels and reducing pain.

*Sex relieves pain*

Orgasm acts as a natural analgesic. Beverly Whipple and Barry Komisaruk of Rutgers University have found that women with conditions such as arthritis and whiplash gain higher pain thresholds through regular orgasms. Midwives advise women to masturbate to orgasm to relieve labour pains.

*Sex strengthens pelvic muscles*

“Regular sex can tone the muscles of your pelvic floor” says Giovanna Ciccarelli, a trainer at NYC’s Equinox Fitness Centre. Gripping a penis with your vagina does what kegel exercises do. So vigorous contractions during orgasm. “Strong pelvic muscles stregthen your posture, back and the abdominals” notes Ciccarelli.

- *Did you know that we can determine if a person is sexually active or not by looking at her skin?*
- *Sex is a beauty treatment. Scientific tests have shown that a woman who has sexual relations produces big amounts of estrogen which makes hair shiny and soft.*
- *The more we make love, the more we have the capacity to do more. A body sexually active releases a higher amount of pheromone. This subtle aroma excites the opposite sex !*
- *Sex relieves headaches. Each time we make love, it releases the tension in brain veins.*
- *Making love can heal a nasal congestion. Sex is a natural antihistaminic. It helps fight asthma and spring allergies.*
- *Sex is not always about penetrating someone. It is mainly about how you feel when you are with that someone special*

**Common sex problems**

- ***General lack of interest in sex*** due to fear, anxiety, depression etc
- ***Impotence***: Failure by the man to have and maintain an erection
- ***Frigidity***: Lack of interest in sex by the woman and failure to experience sexual arousal
- ***Premature ejaculation***: Man reaching orgasm before full penetration is achieved
- ***Sadism***: Man experiencing sexual arousal only when he inflicts pain and humiliation on a woman
- ***Masochism***: Woman experiencing sexual arousal only when she has been exposed to physical pain, torture and humiliation
- ***Peeping Tom***: Person who experiences sexual arousal by watching others making love
- ***Exhibitionist***: Man or woman who attains sexual gratification by exposing his/her nakedness to others.
- ***Fetishism***: Experiencing sexual arousal through touching or watching garments worn by the opposite sex e.g. knickers, belt, neck tie, bra etc

**Safe sex**

- ***Unsafe sex***: Sex practices that offer no protection for either partners e.g. “skin to skin sex”
- ***Safer sex***: Sex practices that offer some protection for either partner e.g. use of condoms
- ***Safe sex***: Sex practices that offer absolute protection for either partner e.g. caressing, masturbating, hot talk e.t.c.

**CHAPTER THIRTEEN: Male Circumcision**

What is male circumcision?

- Circumcision is the removal of a simple fold of skin (the `foreskin' or `prepuce') that covers the head (glans) of the un-erect penis.
- The amount of foreskin skin varies from virtually none to a considerable amount that droops down from the end of the flaccid penis.
- In some men, during an erection, the head of the penis peeks out from the loose foreskin that surrounds it. But in men with a lot of foreskin the head of the penis remains covered, either partially or completely.


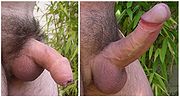


Male circumcision statistics

- According to the [World Health Organization](http://en.wikipedia.org/wiki/World_Health_Organization) (WHO), global estimates suggest that 30% of males are circumcised, of whom 68% are Muslim.
- The prevalence of circumcision varies mostly with religious affiliation, and sometimes culture.
- Most circumcisions are performed during adolescence for cultural or religious reasons; in some countries they are more commonly performed during infancy.

Controversy regarding male circumcision

- Advocates of circumcision argue that MC provides important health advantages which outweigh the risks, has no substantial effects on sexual function or performance, has a low complication rate when carried out by an experienced physician, and is best performed in the [neonatal](http://en.wikipedia.org/wiki/Infant) period.
- Opponents of circumcision typically argue that it adversely affects normal sexual pleasure and performance, is justified by medical myths, is extremely painful, and is a violation of [human rights](http://en.wikipedia.org/wiki/Human_rights).

Common problems with the foreskin

- Many males (young and old) cannot comfortably pull back the foreskin over the glans (knob) of the penis because the foreskin is too tight. If they force, the retraction results in painful cuts.
- Above the age of six or seven, it is important that boys be taught how to clean under the foreskin, making sure the skin is pulled right back. If your son cannot do that he may need circumcising.
- If the foreskin cannot easily be pulled back when the penis is erect, or if that causes pain - this is not only a childhood problem. At puberty, as the penis grows and masturbation begins, problems may emerge.
- Some men have pain on intercourse, which they then try to avoid for that reason. If so, you (and your partner) will benefit from your circumcision just as many boys do. Some men are afraid to admit to this problem, but it is curable by circumcision
- The foreskin is a warm and moist incubator under which infections easily develop. A foreskin that is not constantly cleaned develops a whitish thick, smelly and itchy discharge.
- Where you and your partner keep getting 'thrush' infections. Some call this 'sexual ping-pong'. One keeps passing it back to the other. Of course, you may first try creams or tablets from your doctor or chemist, but if it keeps coming back, circumcision will cure it.

**Male circumcision procedure**

- The adult procedure takes 20-30 minutes under local anaesthetic. Any embarrassment will quickly pass.
- It is important to be able to discuss the procedure with the clinician, and make sure you have a full, not partial, circumcision, which should leave the glans exposed at all times to get maximum benefit.
- Afterwards there can be some pain, as with any cut, but it can be managed with *Paracetamol*. Some clients feel no pain at all.
- The stitches will dissolve, but if any are left after 2 weeks, the clinician/nurse should remove them.
- It will be swollen at first, but intercourse can resume after 4 weeks and careful masturbation earlier.

**Prevention of STIs/HIV**

- The World Health Organization (WHO; 2007), the [Joint United Nations Programme on HIV/AIDS](http://en.wikipedia.org/wiki/Joint_United_Nations_Programme_on_HIV/AIDS) (UNAIDS; 2007), and the [Centers for Disease Control and Prevention](http://en.wikipedia.org/wiki/Centers_for_Disease_Control_and_Prevention) (CDC; 2008) indicate that male circumcision significantly reduces the risk of [HIV](http://en.wikipedia.org/wiki/HIV) acquisition by men during penile-vaginal sex.
- But circumcision only provides minimal protection and should not replace other interventions to prevent transmission of [HIV](http://en.wikipedia.org/wiki/HIV)
- A [meta-analysis](http://en.wikipedia.org/wiki/Meta-analysis) of observational data from twenty-six studies found that circumcision was associated with lower rates of syphilis, [chancroid](http://en.wikipedia.org/wiki/Chancroid) and possibly genital herpes
- A clinical study of 5,925 women from Uganda, Zimbabwe and Thailand found that the circumcision status of their partner did not significantly affect the incidence of [Chlamydia](http://en.wikipedia.org/wiki/Chlamydia), [gonorrhea](http://en.wikipedia.org/wiki/Gonorrhea) or [trichomoniasis](http://en.wikipedia.org/wiki/Trichomoniasis)

**Benefits of circumcision**

- Many older men, who have bladder or prostate gland problems, also develop difficulties with their foreskins due to their surgeon's handling, cleaning, and using instruments. Some of these patients will need circumcising.
- Some older men develop cancer of the penis - about 1 in 1000 - fairly rare. Infant circumcision gives almost 100% protection, and young adult circumcision also gives a large degree of protection.
- Cancer of the cervix in women is due to the Human Papilloma Virus which lives under and on the foreskin from where it can be transmitted during intercourse. The *British Medical Journal* suggests that at least 20% of cancer of the cervix would be avoided if all men were circumcised. Surely that alone makes it worth doing?
- Circumcised men are 8 times less likely to contract the HIV virus (*British Medical Journal*, 2000). (It is very important here to say that the risk is still far too high and that condoms and safe sex must be used - this applies also to preventing cancer of the cervix in women who have several partners.)
- In 2000, a *BBC* television programme showed two Ugandan tribes across the valley from one another. One practised circumcision and had very little AIDS, whereas, it was common in the other tribe, who then also started circumcising. This programme showed how the HIV infection thrived in the lining of the foreskin, making it much easier to pass on.
- As with HIV, so some protection exists against other sexually transmitted infections. Accordingly, if a condom splits or comes off, there is some protection for the couple. However, the only safe sex is to stick to one partner or abstain.
- **Lots of men, and their partners, prefer the appearance of their penis after circumcision,** It is odour-free, it feels cleaner, and they enjoy better sex. Awareness of a good body image is a very important factor in building self confidence.
- *****Orgasm, the culmination of the sex act, is not related to the foreskin, and involves activity of neurones in the hypothalamus of the brain.
- Balanitis which an unpleasant recurrent inflammation of the glans is quite common and can be prevented by circumcision
- Urinary tract infections sometimes occur in babies and can be quite serious. Circumcision in infancy makes it 10 times less likely.
- Teenagers, especially the younger ones, will have almost total ignorance. They may secretly be having problems. Maybe they wish they had been circumcised for either body image or medical reasons. Help them to be informed and aware of their options.

# CHAPTER FOURTEEN: Management of Counselling Services

Management is the process of administering and controlling things. It entails being in charge of, running affairs, regulating a program or programs. The following activities are all part of the process of management: Planning services & activities, Budgeting, Resources mobilization, Implementing activities, Coordinating activities/services, Controlling/Supervising activities, Monitoring activities and Evaluating activities.

A manager is a person who runs, manages and controls the various activities of an organization. The process of running VCT services is quite complex in that counselling involves dealing with peoples’ emotional feelings. The professional touch of a qualified counsellor therefore becomes vital right from the beginning.

A newly qualified HIV/AIDS counsellor is often required to render his/her services at an already established counseling center or establish a new facility. The provision of counselling services entails applying some managerial skills whatever the situation is. The following are the important things a health services manager should consider when planning to establish a new counseling facility or improving an already existing one:

1. **Infrastructure** (counseling building/room): Ensure the room is in a quiet environment preferably away from the hassles of a busy undertaking such as the OPD. The environment should one that provides privacy such that clients can feel free and relaxed to talk about their issues openly. The room should, however, be easily accessible to members of the public but ensure the much needed confidentiality.
2. **Materials** A good counsellor should able to use his/her networking skills to mobilize the following basic materials for the counseling room:
   - Furniture: - At least 3 chairs for counsellor & clients, Small table for writing notes, Lockable cabinet/cupboard
   - Stationary: - Paper, pens, Rule, Hard cover books, stapler, staples, cello tape, scissors, Plain folders, paper clips, paper puncher etc.
   - Test kits: - These can be budgeted for or requested for from networks such as Zambia VCT services.
   - IEC materials - Pamphlets, booklets, brochures on counseling, HIV/AIDS. These can be requested for from the Ministry of Health, Society for Family Health, ZPCT, Family Health Trust, CRS, Kara Counselling etc.
3. **Skilled (qualified) counsellors** & other supportive staff e.g. Lab tech, receptionist etc.
4. **Range of services** that can be availed to the members of the public include the following:

- Counselling: Voluntary counseling for HIV/AIDS, child counseling, couple counseling etc.
  - Testing for HIV and CD4 count
  - PMTCT services
  - Antiretroviral treatment (ARVs)
  - Referrals to Care and Support services e.g. Medical treatment, male circumcision, cervical cancer screening, Home based care, Laboratory tests for TB, Malaria, STIs, CD4 count, viral load count, DBS for infants
  - Health talks
  - IEC materials such as brochures, pamphlets
  - Outreach behaviour change activities
  - Support services e.g. Post-test club
  - Condom promotion

1. **Record keeping:** This entails keeping track of all activities so that the monitoring and evaluating of the VCT services is made easy. Record keeping involves indicating in the daily counselling register at least the following data:

- Total number of clients seen at the VCT centre on a given day
- Numbers of new clients
- Number of old clients
- Number tested
- Number tested positive
- Number of females/males tested positive
- Number tested negative
- Number of females/males tested negative
- Number of couples seen
- Number of couples tested
- Number of discordant couples
- Number of condoms supplied
- Number of referred cases seen
- Number of clients referred elsewhere

1. **Quality control** through the following activities:
   - Refreshers training to enable staff acquire new skills and techniques.
   - Regular staff supervision by a competent senior counsellor to ensure quality control of services that are given to clients
   - Affiliation to professional bodies in order to promote standards
   - Regular meetings with other counselors to exchange ideas
   - Teamwork: Successful counseling stems from efforts by all team members. This involves sharing responsibilities and results among staff.
   - Monitoring activities to ensure that all planned activities are on course and being done by the right people. This is usually done through having regular meetings to review progress and to chart the direction of the activities
2. **Research activities:** Every counselling and testing centre should be regarded as a research centre because the data generated from the centre can be utilized to generate new information that would be of use when planning new activities for the centre.
3. **Policy development:** Every counselling and testing centre is set up using standards that are contained in the guidelines for HIV/AIDS counselling and the code of conduct & ethics booklets by the Ministry of Health and Zambia Counselling Council.
4. **Networking**: Simply means collaborating and forming partnerships with other counselling centers and organizations in order to:

- Share resources
- Share costs
- Share responsibilities
- Reduce the work load
- Avoid duplicating work
- Learn from others

## CHAPTER FIFTEEN: Communication Skills and Report Writing

## Objectives

1. Explain the communication process
2. Identify barriers to effect communication
3. Explain the value of proper record management
4. Identify parts of a formal report

# Communication is without question, at the heart of our every human activity. Since time immemorial, people have primarily attempted to get others to understand them and to act upon our communication. However, that communication is a complex process because even the simplest form of communication can easily lead to miscommunication.

**Communication:** Communication can be defined as a process of sharing information, ideas, feelings and attitudes. Communication is said to be effective only when it achieves its desired response (Murphy & Peck 1972). From the training point of view, if you can not communicate, you can not train.

## The Communication process

The process of communication mainly consists of five elements, namely:-

The Sender → The medium → The Message → The receiver → The feedback

A clear understanding of each of these stages is important as communication can easily malfunction at each stage.

- **The Sender:** The interrelationship of the sender and the receiver profoundly affect the effectiveness of communication.
- **The medium:** The medium is the selected method of conveying a message.
- **The Message:** The message is a written or oral communication that expresses the ideas the sender desires to convey to the receiver. This is the information, a feeling, emotions or an argument.
- **The receiver:** The receiver, sometimes referred to as the perceiver or decoder, is the intended target for the message. The receiver must correctly interpret the message as intended for communication to be effective.
- **The Feedback:** This, without doubt, is a very critical stage as effectiveness of communication is measured here. Decisions to rephrase of entirely change the message composition are made depending on the assessed feedback by the sender. Feedback can be a direct response, an action or even observed facial expressions.

**Non – Verbal Communication (Paralinguistic aspects of communication)**

Beyond the words we use, non-verbal cues play an important role in communication as these add to the clarity of the message, and in some cases even change the message completely. This means that what may be said and what is meant may be poles apart. Non-verbal communication includes the following: Facial expressions, Gestures, Body posture, Vocal cues, Eye contact

# Communication barriers: Communication is not always as uncomplicated as it has been made to appear. As stated earlier, even the simplest form of communication can sometimes result in severe miscommunication. In some case, miscommunication is not only costly, but may also lead to loss of life. Knowledge of possible causes of communication breakdown will, however, assist one in minimizing miscommunication.

| Stage | Possible barriers |
| --- | --- |
| Sender | - Choice of the medium - Choice of words - Knowledge of the sender - Personality (in the case of face to face Communication) - Lack of adequate preparation |
| Message | - Clarity of message content - Shared assumptions |
| Medium | - Inappropriate method |
| Receiver | - Prejudgment of the sender - Interpretation of work - Perception of conveyed message - Attitude - Culture differences |
| Feedback | - Delayed response - Misinterpretation |

# REPORT WRITING

Reports writing is not only a necessary, but critical skill. Regardless of your position, there will be need for you to write reports from time to time. The frequency and intensity of the task will of court depend on the position you hold in your Organisation.

Various types of reports exist, ranging from accident report forms to huge volumes inclusive of appendices. The nature of the subject will determine the type of report to be written. Reports travel upward to supervisors and management policy makers; downwards and horizontally to those who carry out the work and policies; and outward to customers, shareholders, general public and respective government ministries (Murphy and Peck 1972: 540).

# Report structure: A formal report should be structured with clearly labeled sections. These are:

- ***Report title***: A report title should be specific and clearly indicate what the report is about e.g. ‘Report on the launch of PMTCT at Mwembeshi Satellite Station’

| **REPORT**  ON  **LAUNCH OF CHILD COUNSELLING SERVICES**  MENTAL HEALTH RESOURCE CENTRE    20^th^ February 2010  Compiled by Mental Health Unit  (2010) |
| --- |

- ***List of Contents*** such as the following:
- ***Acknowledgements***: Appreciation that is attributed to individuals or organizations who assisted in one way or another
- ***Introduction****:* Overview of what the report is mainly about, including the scope of the work that is being reported. A good introduction describes when it happened, where it occurred, why it took place, how many were involved, the venue where it was conducted, who participated, how long it took e.t.c.
- ***Objectives***: the objectives may sometimes be included in the introduction
- ***Summary or narrative of activities***: Description of daily activities beginning from day 1 up to the last day of the event.
- ***Achievements/Findings***: What were the final outcomes of the event under review? List them one by one.
- ***Constraints***: This refers to all the difficulties that were encountered in the process.
- ***Recommendations****:* What should be done next time to avoid the outlined difficulties.
- ***Appendices***: These are attachments to the report such as the list of participants, time table, pictures or indeed maps.

# *Signature/designation*: Every report should be signed and dated

# Appendix 1: Verbatim Writing

A verbatim is a **word for word** written record of a session. It is written at the end of the counseling session. The counsellor should pay attention to what is being said and done during the session and then reproduce the contents of the discussion in written form at a later time.

Counselling does not allow note taking during the session. However, immediately after the session, a summary of issues discussed is written down after the client is gone. Later you may care to expand the notes to as much verbatim as possible.

-----------------------------------------------------------------------------------------------------------

# A. GENERAL INFORMATION

a) Initials of client :…… d) Length of interview ………………….

1. Date of interview:…… e) Description of session ………………..
2. Place of interview:……. f) Name of counsellor ……………………

# B. KNOWN FACTS: Write any information you know about the client and indicate source of this information.

**C**. **PREPARATION:** Write what you did in preparation for the counselling session, e.g. how you set the room, and other preparatory arrangements. Include the agenda (what you wish to do and, what you don’t wish to do).

# D. OBSERVATIONS: Record what you see when the client comes, e.g. the client’s mood, appearance, mannerisms and gestures. This information helps you to decide how to proceed with the interview (counselling dialogue)

# E. ACTUAL INTERVIEW

1. Each entry must be indicated by initials or a code of the person speaking.
2. Record each entry as direct speech, e.g. “good morning”, or “please sit down”

|  | ACTUAL SESSION | My own thoughts | Teachers’ remarks |
| --- | --- | --- | --- |
| CLT  CNSLR  CLT  CNSLR  CLT  CNSLR  CLT  CNSLR  CLT CNSLR | (knocking)  Come in  (walks in)  Good morning (standing)  Good morning madam (shaking hands)  Please take a sit (pointing to chair)  Thank you  Welcome to the counselling center. My name is Jinx, I am a counsellor here.  My name is Keke I live in Chelston.  How can I be of help to you this morning? | I wonder who that is  This man looks tired  I wonder what he wants |  |

1. All non-verbal expressions or observations must be written in brackets, just after the entry of each speaker.
2. Leave a three centimetre margin on the right hand side of the paper for comments by the trainer

# F. CONCLUSION: Summarise your session under the following headings:

1. Social concerns of the client (what does client think about his situation?)
2. Psychological concerns of the client (How does client feel about his predicament?)
3. Personal critique of your practice (what mistakes did I make?)
4. Any future plans or goals for counseling (what do intend to do when client comes back next time)

# Appendix 2: Journal Writing

Counselling training is a professional journey during which the trainee learns about many aspects of life and how these life issues affect different individuals from a cross section of cultures. A journal is a write up or diary in which a trainee counsellor shares some important lessons picked during the learning discourse involving the trainers and fellow trainees. Some of these lessons may be personal or collective but should be discussed in the journal so that the trainers get a view of the trainee perspective. A good journal is written under the following sub-headings:

## Introduction:

Give an overview of the issues that you have identified (pertaining to your training) which you would like to discuss in your journal?

## My feelings:

What emotions (feelings) have these events and issues provoked in you. Describe the events/issues in details and explain how they felt as a result. Feelings should be emotional feelings and expressed as “*I felt Excited, overwhelmed, disappointed, frustrated, anxious, worried, furious, happy”* e.t.c.

## Lessons I have Learnt about myself

What new things have you begun to realize about yourself as a result of these events. What new things have you begun to realize about your own private life, family life, social life, personality and character? Example: “*After the topic on listening, I discovered that my children are closer to my spouse because s/he listens to them more than I do*”

## Lessons I have learnt about counselling

What new things have you learned about the course you are pursuing which you never knew before? What have these new experiences taught you about the program you are pursuing?

**Conclusion**: what are your closing remarks?

**Appendix 3: The Vocabulary of Feelings** (Hammond, Epworth & Smith, 1978,)

Feelings are the emotions that we experience in different situations. Feelings can either be good, bad or confused but it is always better to pin point the exact feeling that best suits the situation. Below is a catalogue of Strong Intensity Words: Please use them to empathize during role-plays and in journal writing.

| **Happy feelings** | **Sad feelings** | **Caring feelings** | **Inadequate feelings** | **Fearful feelings** | **Confused feelings** |
| --- | --- | --- | --- | --- | --- |
| Thrilled  On cloud 9  Ecstatic  Overjoyed  Excited  Elated  Sensational  Exhilarated  Fantastic  Terrific  On top of the world  Happy  Turned on  Euphoric  Enthusiastic  Delighted  Marvelous  Great | Dissolute  Dejected  Hopeless  Alienated  Depressed  Gloomy  Dismal  Bleak  In despair  Empty  Barren  Grieved  Grief stricken  Grim | Tenderness toward  Affection for  Captivated to  Devoted to  Adoration  Loving  Infatuated  Enamored  Cherish  Idolize  Worship | Worthless  Good for nothing  Washed up  Powerless  Helpless  Impotent  Crippled  Inferior  Emasculated  Useless  Finished  Like a failure | Terrified  Frightened  Intimidated  Horrified  Desperate  Panicky  Terror stricken  Stage fright  Dread  Vulnerable  Paralyzed | Bewildered  Puzzled  Baffled  Perplexed  Trapped  Confounded  In a dilemma  Befuddled  In a quandary  Confused |

| **Hurt feelings** | **Angry feelings** | **Lonely feelings** | **Guilt/shame feelings** |
| --- | --- | --- | --- |
| Crushed  Destroyed  Ruined  Degraded  Pained  Wounded  Devastated  Tortured  Disgraced  Humiliated  Anguished  At the mercy of  Cast of  Forsaken  Rejected  Discarded | Furious  Enraged  Seething  Outraged  Infuriated  Burned up  Pissed off  Fighting  Mad  Nauseated  Violent  Indignant  Hatred  Vengeful  Hateful  Vicious | Isolated  Abandoned  All alone  Forsaken  Cut off | Sick at heart  Unforgivable  Humiliated  Disgraced  Degraded  Horrible  Mortified |

**Appendix 4: Consent Forms (*sample*)**

| **CONSENT**  FOR INDIVIDUAL HIV COUNSELLING/TESTING  I ………………………………………… understand that I will be tested for HIV, the implications of which have been explained to me. I further declare that I am aged 18 years and above and the results of the test will be given to me.  Declared this ………………… day of ……………… 20……………….. in the presence of ……………………………………………….. (counsellor)  Signature or thumb print (client): ………………………………….  Signature of counsellor: ………………………………………….. |
| --- |

| **CONSENT**  FOR COUPLE HIV COUNSELLING/TESTING  We ………………………………………… understand that we will be tested for HIV, the implications of which have been explained to us. We further declare that we are both aged 18 years and above and the results of the test will be given to us.  Declared this ………………… day of ……………… 20……………….. in the presence of ……………………………………………….. (counsellor)  Signature or thumb print (client): ………………………………….  Signature or thumb print (client): ………………………………….  Signature of counsellor: ………………………………………….. |
| --- |

**Appendix 5: Guide for Psychosocial Counselling Training Practicum Session Assessment**

Name of Trainee:………………………………………………………………………………..

Date:………………………………………………………………………………………………….

**Instructions**

For each behaviour, knowledge and attitude listed below, tick the number that most accurately reflects the present level of performance. Write comments remarks on the extreme right column.

**Key to Rating**

5 - Demonstrates outstanding competence in knowledge, skills and attitude

4 - Competent performance

3 - Average performance needs improvement

2 - Below average performance

1 - Poor performances repetition

0 - Omissions of knowledge skills and attitudes

|  | **COUNSELING BEHAVIOUR/SKILLS/ATTITUTE** | **5** | **4** | **3** | **2** | **1** | **0** |
| --- | --- | --- | --- | --- | --- | --- | --- |
| 1 | Welcomes and makes client comfortable |  |  |  |  |  |  |
| 2 | Makes introductions, and initiates a working relationship |  |  |  |  |  |  |
| 3 | Explains the counselling contract |  |  |  |  |  |  |
| 4 | Encourages the client to talk |  |  |  |  |  |  |
| 5 | Demonstrates active listening |  |  |  |  |  |  |
| 6 | Shows respect for the client |  |  |  |  |  |  |
| 7 | Provides clients with accurate information |  |  |  |  |  |  |
| 8 | Demonstrates empathic understanding |  |  |  |  |  |  |
| 9 | Demonstrates non possessive warmth |  |  |  |  |  |  |
| 10 | Displays congruence/genuineness |  |  |  |  |  |  |
| 11 | Portrays acceptance |  |  |  |  |  |  |
| 12 | Picks feelings and emotions |  |  |  |  |  |  |
| 13 | Reflects feelings and emotions |  |  |  |  |  |  |
| 14 | Asks open ended questions |  |  |  |  |  |  |
| 15 | Answers questions appropriately |  |  |  |  |  |  |
| 16 | Directs flow of the session |  |  |  |  |  |  |
| 17 | Facilitates identification and prioritization of problems and options |  |  |  |  |  |  |
| 18 | Uses silence effectively |  |  |  |  |  |  |
| 19 | Facilitates discussion on planning |  |  |  |  |  |  |
| 20 | Manages time effectively |  |  |  |  |  |  |
| 21 | Observes client’s non-verbal cues |  |  |  |  |  |  |
| 22 | Clarifies issues |  |  |  |  |  |  |
| 23 | Paraphrases client statements as necessary |  |  |  |  |  |  |
| 24 | Summarises the counselling session |  |  |  |  |  |  |
| 25 | Engages client in commenting on the session |  |  |  |  |  |  |

**Marks Available**:…………… **Marks Obtained**:………………

Comments by Trainee Counsellor: …………………………………………………………………………

……………………………………………………………………………………………………………….

Signature:……………………

Comments by supervisor: ………………………………………………………………………………….

………………………………………………………………………………………………………………

**Appendix 6: PSYCHOSOCIAL COUNSELLING COURSE - TIME TABLE**

**Course objectives**

At the end of the course, participants should be able to:

1. Acquire knowledge on the key facts about HIV and AIDS
2. Appreciate the self-concept
3. Demonstrate the use of counselling skills
4. Show understanding of counselling in special situations
5. Demonstrate critical thinking skills and stress management skills
6. Show understanding of common public health issues

|  | ***08:30*** | ***10:30*** | ***11:00*** | ***13:00*** | ***14:00*** | ***15:30*** | ***15:45-17:00*** |
| --- | --- | --- | --- | --- | --- | --- | --- |
| Day 1 | Registration, Introductions, Objectives, Expectation, Group norms | T | Self-awareness |  | Facts about HIV/AIDS: Prevention strategies | T | HCT guidelines |
| Day 2 | eMTCT | E | Psychosocial and Cultural Issues of HIV/AIDS |  | Introduction to Counselling Theories and Practice | E | Counselling interventions |
| Day 3 | Pre-test counselling and Post-test counselling | A | Attending skills (relationship building) demo Listening skills (demo) | L | Probing skills (demo) | A | Journal writing assignment |
| Day 4 | Challenging skills (demo) |  | Paraphrasing skills (demo) | U | Empathy skills (demo) |  | Crisis intervention |
| Day 5 | STIs |  | Male circumcision | N | Cervical cancer screening |  | Verbatim writing assignment |
| Day 6 and 7 | **VERBATIM WRITING** | | | | | | |
| Day 8 | Human sexuality | T | Treatment of Alcohol and Drug Abuse | C | Counselling children and adolescents | T | Case study |
| Day 9 | Couples counselling | E | Stress Management | H | Mental Health Aspects of Counselling | E | Case study |
| Day 10 | Emotional care to cancer patients | A | Working with Groups |  | Death and dying (counselling) | A | Critical thinking |
| Day 11 | Provider Initiated HCT |  | Professional burnout |  | STUDY |  | STUDY |
| Day 12 | Written Exam |  | Evaluation, close |  |  |  |  |

**Bibliography**

Central Statistical Office (2007) ***National Demographic Health Survey***. Lusaka: CSO

Chiboola, H (Ed 2006) ***HIV/AIDS Counselling***: ***A HANDBOOK***. Lusaka: Lioness and Dove

[http://www.malecircumcision/googlesearch [accessed](http://www.malecircumcision/googlesearch%20%5baccessed) on 20^th^ January 2010]

Catholic Relief Services (2008) ***Psychosocial Support to HIV infected Children and Adolescents***. Kampala: CRS

Lewis, E. C (1970) ***The Psychology of Counselling***. New York: Hort

Ministry of Health (2007) ***HIV/AIDS COUNSELLING***: **A *Generic Training Manual***. Lusaka:

Associated printers Limited

Ministry of Health (1999) ***HIV/AIDS IN ZAMBIA: Background, Projections, Impacts and***

***Interventions***. Lusaka: Ministry of Health

Ministry of Health (2000) ***HIV/AIDS Counselling Guidelines***. Lusaka: UNZA Press.

Ministry of Health (2009) ***Treatment Technical Guidelines for frontline health workers***.

Lusaka: MOH

Ministry of Health (2004) ***PMTCT guidelines for health care professionals***. Lusaka

(unpublished)

National AIDS Council (2004) ***National Guidelines on Management and Care of Patients***

***With HIV/AIDS***. Lusaka. NAC

Ponder, T, (1983) **How to Avoid BURNOUT**. California: Pacific Press Publishing Association

UNODC (2008) Treatnet II programme: ***VOLUME B*** (***Psychosocial counselling for the***

***treatment of alcohol and drug abuse disorders***). UNODC

Zambia Counselling Council (2001) ***CODE OF ETHICS FOR COUNSELLORS***. Lusaka: UNZA Press
