# Supplementary material for: Peer community health workers improve HIV testing and ART linkage among key populations in Zambia: retrospective observational results from the Z‐CHECK project, 2019–2020
Source: J Int AIDS Soc. 2022 Nov 1;25(11):e26030. doi: 10.1002/jia2.26030 (PMC9624072; doi:10.1002/jia2.26030)
Supplement: Supplementary file 2 — Participant Workbook [file JIA2-25-e26030-s001.docx]

2016


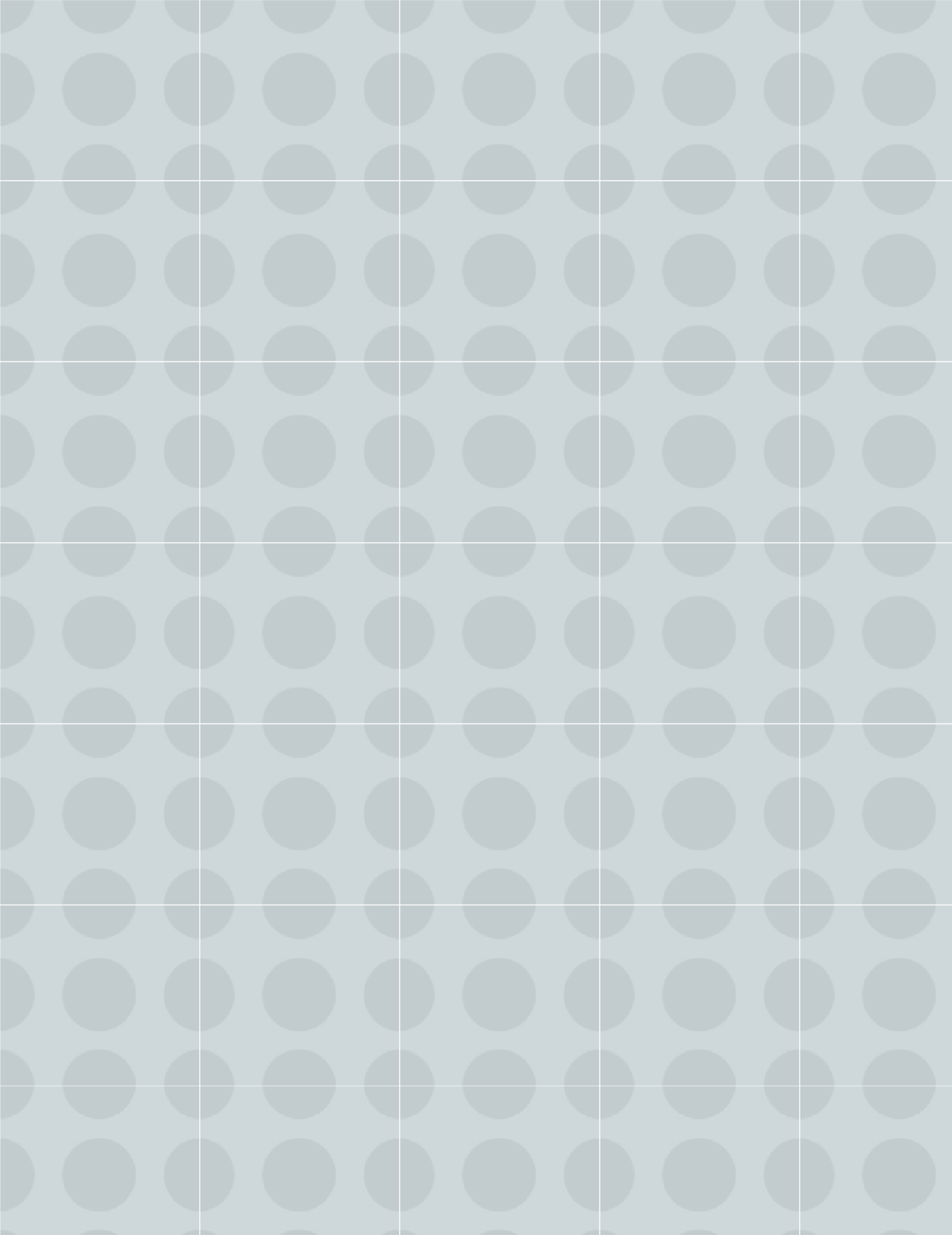


Providing Key Population-Friendly Services: A sensitivity training for health care workers (HCWs)

Participant Workbook

**MODULE 1** PREPARING FOR THE TRAINING

**Introductions**

Who are the trainers?

**Ground Rules**

**Learning Objectives**

- Learn more about:
  - Key populations in the context of an HIV epidemic
  - How stigma and discrimination affect key populations
  - Overcoming stigma and discrimination to provide competent care
- Understand the importance of getting to know every client
- Learn techniques for identifying clients’ unique risks and needs
- Improve ability to provide appropriate care based on individual client **Notes on Training Agenda**

**MODULE 2**

KEY POPULATIONS, HIV AND HIV PREVENTION & RESPONSE FOR KEY POPULATIONS

**Learning Objectives**

- Define key populations in the context of an HIV epidemic
- Understand concepts related to HIV among key populations including:
  - Summarize why key populations are at higher risk for HIV
  - Understand the prevalent and overlapping risks between the key population groups
  - Explain why key populations experience stigma and marginalization and how it affects them
  - Differentiate between key populations and other vulnerable populations
  - Describe the comprehensive response to HIV for key populations

**Understanding Risk and Vulnerability**

| **Factors that put people at risk for HIV** | **Factors that prevent people from using HIV**  **services** |
| --- | --- |
|  |  |

**Who are Key Populations?**

- In the context of an HIV epidemic
- Key populations are
  - People who inject drugs
  - Sex workers
  - Men who have sex with men
  - Transgender populations

**What makes them key populations?**

- Disproportionately affected by HIV
- Engage in behaviors that:
  - Put them at high risk for HIV
    - Injection drug use, sharing of injection equipment
    - Other drug use
    - Unprotected, condomless vaginal and anal sex
    - Multiple sex partners
  - Are socially unacceptable
  - Often criminalized
  - Experience stigma and discrimination for engaging in these behaviors
- A comprehensive package of services has been defined to respond to HIV among key populations

**Key Populations versus Other Vulnerable Populations**

|  | High burden of HIV? | Behaviors are highly stigmatized? | Behaviors are illegal or criminalized? | Low service use? |
| --- | --- | --- | --- | --- |
| Key Populations   - PWID - SWs - MSM - TG | YES | YES | YES  in many countries | YES |
| Other Vulnerable Populations   - Mobile pops/migrants - Clients of SWs - Women - Youth - Displaced pops | MAYBE  context- specific | MAYBE  context-specific | NO  some exceptions | MAYBE  context-specific |

**Defining Sex and Gender**


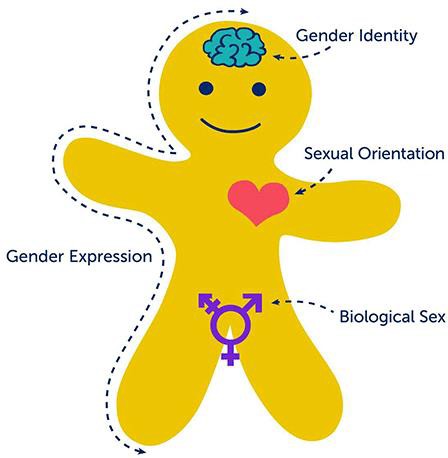


- Biological sex
  - A medical term that refers to the chromosomal, hormonal, and anatomical characteristics that are used to classify an individual as female or male.
  - Intersex is an umbrella term that refers to a variety of conditions in which a person is born with a reproductive or sexual anatomy that doesn’t seem to fit the typical definitions of male or female
- Gender expression
  - The external display of one’s gender through a combination of appearance, attitude, social behavior, and other factors, generally measured on a scale from masculine to feminine
  - Gender norms: culturally-defined set of roles, responsibilities, rights and obligations associated with being male or female
- Gender identity
  - A person’s deeply felt internal and individual experience of gender, which may or may not correspond to their biological sex
  - Transgender: refers to an individual whose gender identity is different from their biological sex
- Sexual orientation
  - An enduring emotional, romantic, or sexual attraction primarily or exclusively to people of a particular gender
  - Heterosexual and homosexual are two commonly used categories of sexual orientation

People who Inject Drugs (PWID) and HIV

- HIV Risk:
  - Sharing of injection equipment
  - Unsafe sex under influence of drugs
  - Addiction can fuel sex exchange
  - Type of drug used, quantity and pattern of use, and administration route impact risk
- Impact:
  - PWID account for 10% of all people infected with HIV globally
  - Female PWID often face unique challenges and vulnerabilities

Sex Workers (SWs) and HIV

- Sex work
  - Can be performed by males, females, TG men and TG women who exchange sex for money
- HIV risk in the context of sex work
  - Unprotected vaginal and anal sex
  - Multiple and concurrent sexual relationships
  - Variations in formality of employment, venue and length have impact on HIV risk
    - Organized versus informal?
    - Street-based versus brothel-based?
    - Short-term versus long-term?

**HIV among SWs**

Impact

- Up to 30% of all HIV infection attributed to sex work
- Female SWs over 13 times as likely to be infected with HIV compared to women in general population


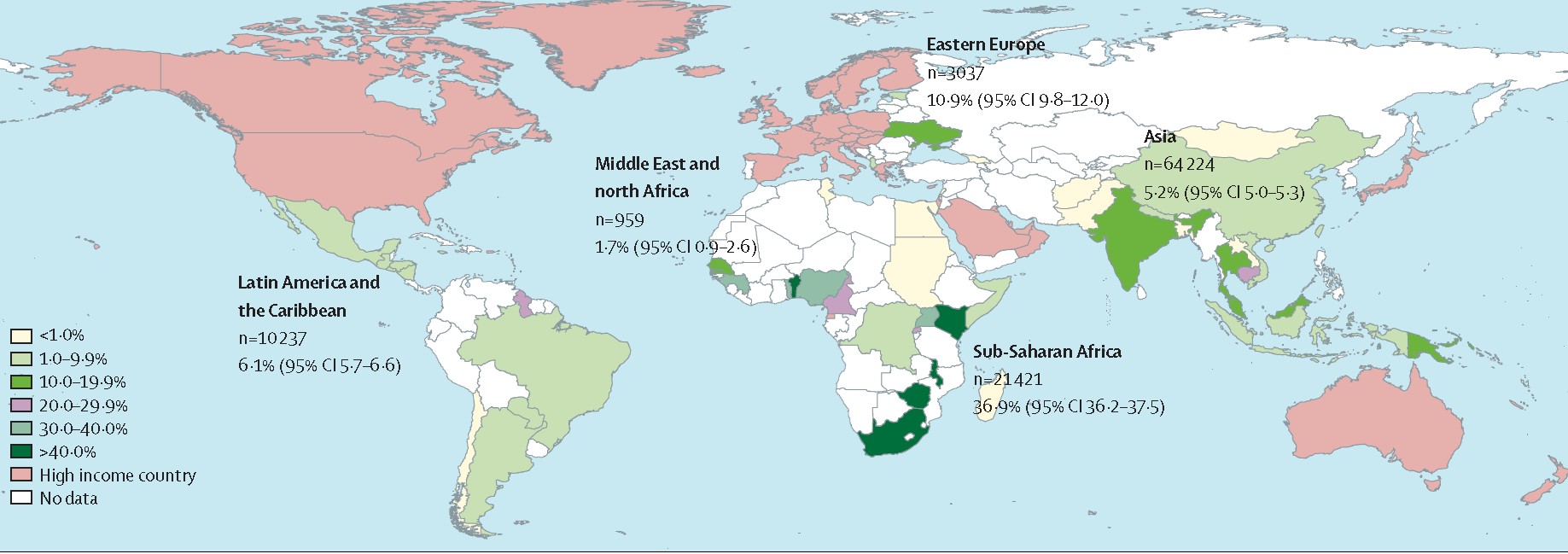


(Baral et al 2012) **Men who have Sex with Men (MSM) and HIV**

- MSM exist in all countries in cultures regardless of sexual orientation
- HIV Risk
  - Unprotected receptive and insertive anal sex carries higher HIV transmission risk than vaginal sex
  - High frequency sex, number of lifetime partners
  - Injection drug use and use of amphetamine-type stimulants
- Impact
  - Up to 10% of all new HIV infections transmitted through male to male sex
  - MSM 19 times more likely to be infected with HIV compared to general population

**HIV among MSM**


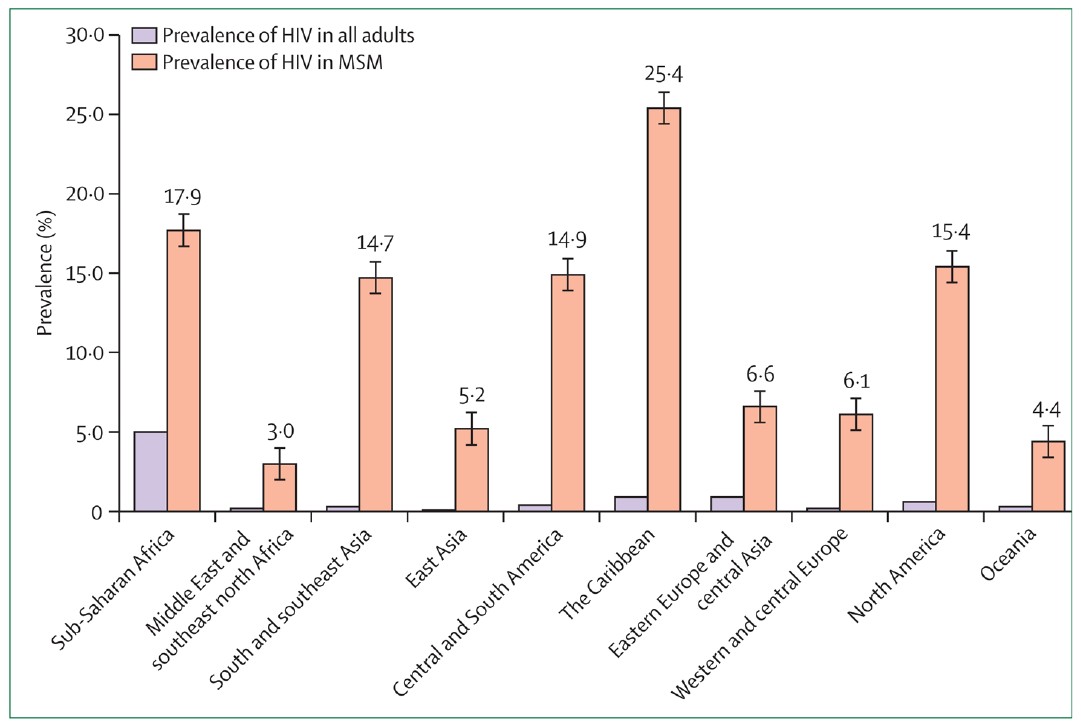


(Beyrer et al 2012)

**Transgender persons (TG) and HIV**

- TG persons
  - Any person whose gender identity or expression differs from their biological sex at birth
    - Transgender women: born biologically male and identify as female gender
    - Transgender men: born biologically female and identify as male gender
  - Includes those between male-female binary
- HIV Risk: Heightened risk is generally linked to position outside social and cultural mainstream
  - Unprotected vaginal and anal sex
  - Multiple sex partners
  - Drug use
  - Sex work

**HIV among TG**

Impact

- TG women are 48 times as likely to be infected with HIV compared to all adults
- More data needed


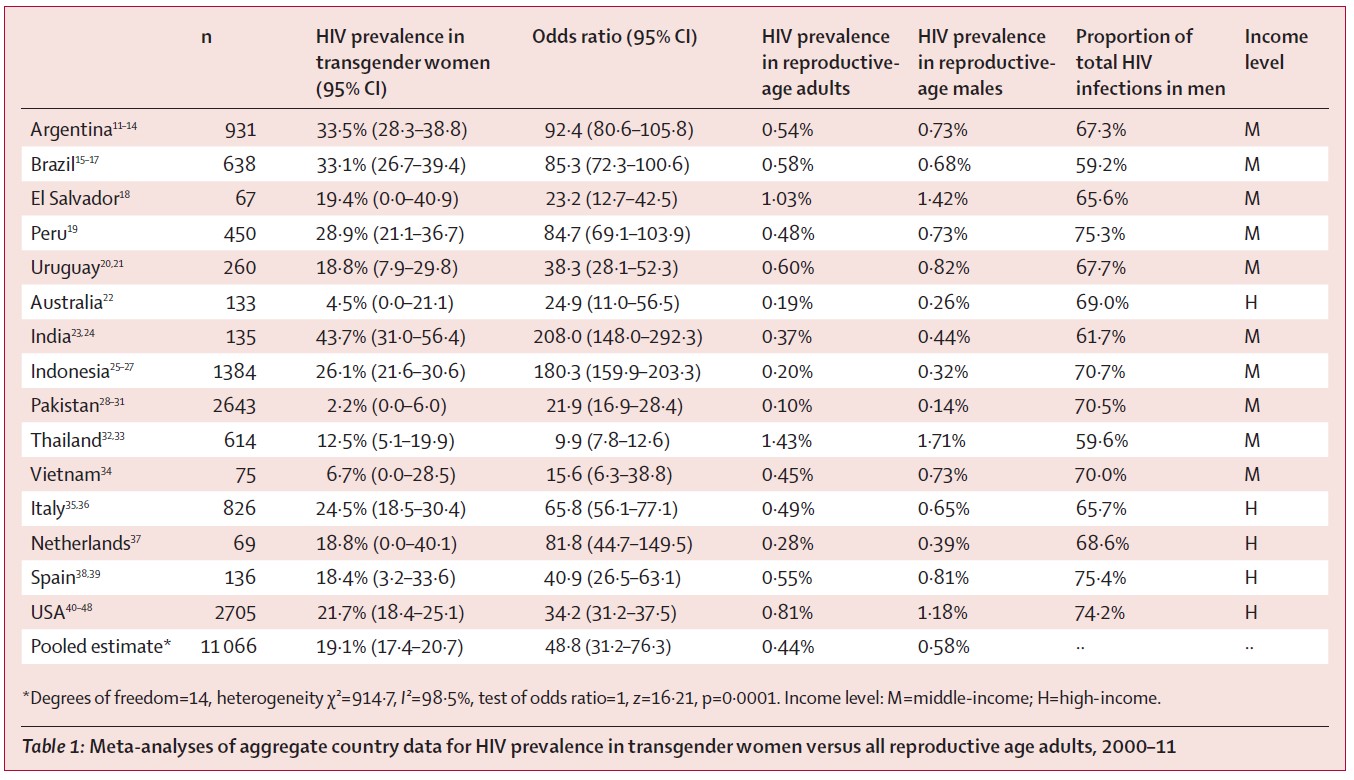


(Baral et al 2013)

**HIV among Key Populations**

- Key populations are disproportionately affected by HIV globally
- Even in countries with high HIV rates in the general population, HIV has a disproportionate impact on key populations
- Stigma and discrimination limits access to HIV prevention and treatment services

Overlapping Risk Behaviors


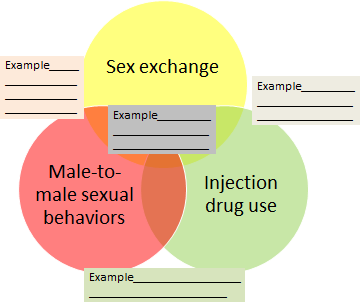


**Low Use of HIV Services among Key Populations**

- Very low use of HIV prevention & treatment services among key populations
- Barriers include:
  - Fear of police harassment and arrest
  - Fear of discrimination by health care providers
  - Services don’t meet unique needs
  - Lack of resources
  - Others?

**Comprehensive Package of HIV Services for Key Populations**

- Peer education and outreach
- Sexual and drug use assessment and risk reduction
- Condoms and condom-compatible lubricants
- HIV Testing and Counseling
- ART for all KP living with HIV
- Post Exposure Prophylaxis (PEP)
- Prevention and management of co-morbidities including STIs, TB, Hepatitis B & C, and mental health disorders
- Pre Exposure Prophylaxis (PrEP) for MSM and uninfected partners in serodiscordant couples
- Harm reduction for People Who Inject Drugs (PWID): Needle and Syringe Programs (NSP), Opioid Substitution Therapy (OST) and Opioid overdose prevention and treatment
- Reproductive health services including PMTCT for women of child-bearing age and pregnant women

How does providing a comprehensive package of services for key populations different than providing services to other groups?

- Outreach essential part of package
- Difference in risk reduction messages
- Appropriate commodities available

**Critical Enablers to Key Population Programs**

- Supportive legislation and policies
- Addressing stigma and discrimination
- Community empowerment
- Addressing violence against KPs

**Notes on Local Epidemiology**

**Module 2 Summary Points**

**MODULE THREE**

THE HEALTH CARE PROVIDER AND KPS

**Learning Objectives**

- Define stigma and discrimination
- Describe how stigma and discrimination affect vulnerability and risk, particularly among key populations
- Differentiate between facts and myths related to KPs
- Identify ways to address stigma and discrimination in your workplace
- Separate personal beliefs from ability to provide competent care
- List characteristics of an effective HCW

**An Overview of Stigma and Discrimination**

- What is stigma?
  - Strong negative feelings towards a person, group or characteristics
  - Significant disapproval of a person or quality that’s considered to be different
  - An attribute or quality that shames an individual or group in the eyes of another
- What is discrimination?
  - Differential and inferior treatment based on a quality or characteristic
  - Prejudiced treatment based on actual or perceived membership in a certain group

**Personal Experiences of Stigma and Discrimination**

Reflect on personal experiences of stigma and discrimination; a time when you were treated differently by other people

**Stigma and Discrimination towards Key Populations**

- People tend to have strong negative feelings about drug use, certain sexual orientations and sexual behaviors. Therefore, key populations face widespread stigma because of their identities and behaviors. This leads to discriminatory treatment in a variety of settings including in healthcare settings.
- Unfortunately, key populations continue to face stigma and discrimination in health care settings. This has been documented in many research studies. These negative attitudes and behaviors by health care professionals are a serious problem. They discourage members of key populations from seeking needed services.
- We can see clear evidence that key populations are discouraged from using health care services. Key populations have a disproportionately high burden of HIV and low rates of service utilization.
- Given their front-line role in the HIV response, stigma and discrimination toward key populations by health care workers can undermine the overall effectiveness of HIV prevention, care and treatment efforts. The World Health Organization therefore recommends training to sensitize health care workers to serving key populations.
- Many key populations have come to expect stigma and discrimination in health care settings. The resources in your workbook provide more information on this. Health care workers who anticipate the expectation are better prepared to provide better care to their clients.

**Effects of Reducing Stigma and Discrimination among Key Populations**

- Greater *availability* of services
- Better *access* to services
- Improved *uptake* of services
- Higher levels of *retention* in services

**Cycle of Stigma, Discrimination and Risk**

Myths and Stigmatizing beliefs

HIV risk and vulnerability

Stigma

Discrimination

**Where do Stigmatizing Attitudes and Myths Come From?**

- Lack of knowledge or understanding
- Lack of information or misinformation
- Ignorance
- Religious or cultural beliefs
- Society’s norms and expectations
- Fear

**Myths Related to Key Populations**

| Myth | How to respond/Why its untrue |
| --- | --- |
| Drug users cannot  adhere to HIV treatment |  |
| Sex workers never use  condoms |  |
| Sex workers do not have  stable partnerships |  |
| Being gay is a choice |  |
| People only have sex  with either men or women |  |
| People can stop using  drugs whenever they want |  |
| Transgender people are  mentally disturbed |  |

**Actions to Address Stigma and Discrimination towards Key Populations**

| Suggested Action | Why and how? |
| --- | --- |
| Get to know any key  populations who visit your clinic to break down stereotypes |  |
| Treat clients equally |  |
| Remind staff to treat all  clients with respect |  |
| Discourage use of language  that is stigmatizing (druggie, homo, hooker, etc) |  |
| Focus on health risks and  needs |  |
| Share case studies from  interactions with clients that are key populations |  |

**Providing Key Population-Friendly, Competent Services**

Why provide key population-friendly services?

- It’s a legal and ethical obligation
- It has an impact on the broader community, not just on key population
- Health care workers should think about the broader community where key populations live and work
- It addresses comorbid conditions that are common among key populations including TB, STIs and Hepatitis
- Providers will have KPs as clients, whether or not they know it or like it: Key populations are a part of every country and every community in the world

General considerations for making health care facilities friendly to all clients, including key populations

- Establish welcoming environment
- Establish trusting and supportive relationship with clients
- Providing services and referrals in a non-discriminatory manner
- Become familiar with local ‘scene’ and use community organizations for this

**Personal Beliefs versus Competent Care**

As a health care worker, you may have to keep your personal beliefs separate from providing this level of competent care, depending on what those beliefs are. The following principles should be considered:

- Accept that our goal is to treat people with dignity, respect and the best health care possible
- Accept that our goal is to reduce HIV transmission and improve health in the communities we serve
- Accept that we may not personally agree with a client’s behaviors
- Accept that talking about sexual relationships, homosexual behaviors and drug use may bring up strong feelings for us
- Accept that success may be defined as risk reduction, not risk elimination

**Characteristics of an Effective Health Care Worker**

- Informed
- Non-judgmental, neutral
- Confidential
- Engaging
- Pragmatic
- Client-centered

**Module 3 Summary Points**

**MODULE FOUR**

GETTING TO KNOW YOUR CLIENTS: WHAT AND WHY?

**Learning Objectives**

- List benefits of conducting a risk assessment
- Identify barriers to conducting risk assessment and list ways to overcome them
- Describe when to conduct a risk assessment
- Provide clients with appropriate messages, services and referrals

**What is a Risk Assessment?**

- A conversation between a health care worker and their client guided by a few key questions
- Goal: identify client characteristics that will improve quality of care
- Getting enough information to know:
  - What prevention messages are needed?
  - What service referrals are needed?
  - What are potential barriers for client?
- Goes beyond a routine assessment; asking more than:
  - “What brings you in today?”
  - “What medical problems are you having?”

**What is Assessed**

- Sexual behaviors
  - Number of partners
  - Gender of partners
  - Types of sex
- Drug use
  - Medical or recreational
  - Type of drugs
  - Type of use
- Medical history
  - HIV/STD/TB/Hepatitis diagnoses
  - Chronic and acute conditions
- Contextual issues
  - Mental health
  - Employment
  - Social support

**Benefits of Conducting a Risk Assessment** For the client:

- Client can think about behaviors that affect risk
- May motivate behavior change
- Opportunity to ask questions
- Normalizes the process: all clients know to expect questions and no one feels singled out

For the provider:

- Assists in clinical exam and intervention
- Provides direction for giving messages and referrals
- Increases skill and comfort in talking about sensitive issues
- Provide better care to clients

For the community at large:

- Those at highest risk of becoming infected or transmitting HIV are identified and served
- Services get better reputation for being accessible and acceptable leading to higher uptake

**Overcoming Challenge of Conducting Risk Assessments**

| Challenge | Response/Strategy to overcome it |
| --- | --- |
| Not enough time |  |
| Not enough  privacy/client not alone |  |
| Language barriers |  |
| Client or provider  discomfort |  |
| Culturally insensitive to  discuss these issues |  |
| Provider fear of issues  that may arise |  |
| Provider discomfort |  |

**When to Conduct a Risk Assessment**

- Routinely with all clients, regardless of what a client looks like
- When confidentiality can be assured
- Alongside routine assessment
- After client understands purpose and importance

**What Messages, Services and Referrals can I provide Clients with?**

| Risk Reduction Messages | Services | Referrals |
| --- | --- | --- |
| - Example: Condom use during anal sex | - Example: STI testing | - Example: HIV test |

**Module 4 Summary Points**

**MODULE 5**

HOW TO GET TO KNOW YOUR CLIENT: CONDUCTING RISK ASSESSMENTS

**Learning Objective**

- Effectively conduct risk assessments

**Notes on what was Observed during Role Plays**

- How did the discussion begin?
- What types of questions were used?
- How did the health care worker react and respond to the client?
- What helped the client feel more comfortable and open?
- Other notes

**11 Tips for Conducting Risk Assessments**

1. Begin by assuring confidentiality
   - For a risk assessment to be effective, the client must feel safe.
   - Remember that your client is more likely to open up after you assure clients that confidentiality will be maintained.
   - You can begin your conversation by saying phrases like:
     - “Everything you say will remain between you and me”
     - “You have a right to privacy and confidentiality that I will respect”
     - “It is my ethical and legal obligation as a health care provider to protect your privacy”
2. Convey routine nature of risk assessments
   - Use language that emphasizes that this type of discussion happens with all patients.
   - It is important that your client does not feel like they are being singled out.
   - Consider phrases like:
     - “I’m going to ask you some questions that I ask all my clients, because they have a big impact on people’s health”
     - “The following topics are standard”
     - “These topics are discussed with all clients”
3. Acknowledge that personal and sensitive information will be assessed
   - Giving the client an introductory notice that you are about to talk about personal topics will help prepare them and may result in them being more open.
   - Consider phrases like:
     - I’m going to ask you about some personal topics. I talk to all my clients about these topics because they affect your health. Many people find it hard to discuss these issues at first.
     - The next set of questions cover sensitive information, but are routinely discussed with all clients at this clinic.
     - It may be uncomfortable to discuss some of these topics at first. I talk to all my clients about these issues and am able to provide the best care to those who are open.
4. Use exploratory questions, open-ended questions
   - Better for engaging in conversation rather than interviewing
   - Open-ended questions allow client to explore their risks and needs, open up and speak freely about their experiences
   - They do not have an expected answer like “yes” or “no”
   - Questions that start with “what” or “how” are particularly useful
   - Phrases like “tell me about” or “it would be helpful to know about” are also useful
   - 5 second rule: Giving clients time to respond
5. Go from general to specific
   - Most common way to arrange questions when getting to know client
   - Questions go from broad to narrow
   - Helpful to imagine a funnel
   - Use a funnel to think about asking clients about sexual risk and behaviors

Tell me about current or recent sexual relationships

How many/what kind of partners

What kind of sex

Use condoms and lube

1. Use the types of words the client is using
   - Creates a stronger sense of understanding and connectedness
   - Ensure client is comfortable as their own language is emphasized
   - Okay to ask for clarification when unsure of term or wording used by client
2. Reinforce healthy behaviors
   - Give client praise when you can
   - Clients likely to receive a lot of advice and suggestions in the context of accessing health care
   - Recognize and commend healthy behaviors when you can
   - Recognize and commend positive contextual factors when you can
3. Remain neutral when hearing sensitive information
   - Normalize potentially sensitive behaviors verbally
   - Maintain neutral body language
     - Smiling or relaxed mouth
     - Nodding
     - Relaxed arms and legs
     - Making eye contact
   - Avoid cold and judgmental body language
     - Looking away
     - Rolling eyes
     - Crossed arms and legs
     - Strong gestures or sudden movements in response to client
4. Focus on risk reduction
   - After learning about client, the next step is to help reduce risk and improve health
   - Remember that success is defined as risk reduction
   - Abstinence-only messages are not effective or appropriate
   - Goals for clients should be achievable and realistic
5. Affirm concerns
   - Clients may express concern in response to disclosing sensitive information
   - To help ease this concern, provide the client with affirmation of their concerns
     - “I’m glad you told me this.”
     - “I know it isn’t east to talk about this, and I appreciate your honesty.”
     - “I understand why you may be concerned, but let me see how I can help you with that”
   - Be prepared for what you might hear
   - Ask your clients to explain when unfamiliar with what they are saying
6. Give clear and consistent messages
   - After getting to know clients, provide them with messages on
     - Risk reduction behaviors
     - Use of additional health services
   - What makes messages clear and consistent?
   - Messages can also be called goals
     - Require client’s action
     - Require health care provider to follow-up

**Reflection**

- Which tip did you find the most helpful and why?
- Which tip would be hard for you to implement and why?
- Which ones do you have questions about or require further explanation?

**Guiding Questions**

- Two helpful tips specific to asking client questions
  - Using open-ended, exploratory questions
  - Go from general to specific, like a funnel
- Keep in mind
  - The goal is to provide client-centered care
  - Find out enough information to provide highest quality of care

**For each topic…**

- Start with an overview question
- Continue with more specific questions and probes, as you receive positive responses
- Stop when
  - You have enough information to give prevention message, service or referral
  - It is clear that line of questioning is not relevant or appropriate for client
  - Move on to the next topic

**Specific Guiding Questions**

- To Begin
  - Tell me about yourself
  - What brings you in today?
  - What can I do for you?
- Sexual Behaviors
  - Tell me about your current or sexual relationships
    - Do you have sex with men, women or both?
  - Tell me about the type of sex you have with these partners. Do you practice oral, anal and/or vaginal sex?
    - If you practice anal sex, are you the insertive or receptive partner?
  - How often do you use condoms during each kind of sex?
    - What kind of lubrication do you use with condoms?
    - How do you access to condoms and lubricants?
  - What (else) are you doing to protect yourself from HIV and other sexually transmitted infections?
- Illicit Drug Use
  - What types of medications or treatment do you take on a regular basis?
  - What is your experience using drugs from a non-medical source?
    - For what reason do you use the drugs?
    - What kind of drugs?
    - How often?
    - How do you use each kind?
      - If injecting, how you get injection supplies?
      - Who do you share supplies with?
- Medical History
  - Tell me about other medical problems you have dealt with
  - To your knowledge, have you even been tested for or diagnosed with HIV, other STIs, TB or Hepatitis?

**Module 5 Summary Points**

**MODULE 6** SUMMARY AND REVIEW

**Learning Objectives**

- Summarize the main points from this training
- Identify specific areas of change to address in your health facility
- Set a goal for change
- Complete the course assessment correctly **Summary Points from the Trainings**

**Action Planning**

- What can you do to make health care services more accessible to high risk clients, including key populations?
- Action planning steps:
  - Understand barriers to key populations accessing health services
  - Identify underlying cause of barrier
  - Set goal that will be achieved by addressing barrier and its cause
  - Break down large barrier into smaller action steps, if needed

| **Challenge** |  |
| --- | --- |
| **Cause(s)** |  |
| **Goal** |  |
| **Time needed** |  |
| **Resources needed** |  |
| **People involved** |  |
| **Goal is reached when…** |  |
| **Challenges to achieving goal** |  |

**COURSE EVALUATION (7 minutes)**

Take some time to complete the evaluation below. Be honest, as your responses will help improve the training in the future. Your responses will be anonymous; you do not have to include your name.

Strongly Agree

Agree Neutral Disagree Strongly

Disagree

1. I obtained a great deal of practical knowledge in this course

⃝ ⃝ ⃝ ⃝

1. The course objectives were clear ⃝ ⃝ ⃝ ⃝
2. My understanding of key populations is clear ⃝ ⃝ ⃝ ⃝
3. The activities in this training were interactive and appropriate

⃝ ⃝ ⃝ ⃝

1. The pace of the training was too fast ⃝ ⃝ ⃝ ⃝
2. The content of the training was too complicated and difficult to understand
3. The trainers were able to answer all questions

⃝ ⃝ ⃝ ⃝

⃝ ⃝ ⃝ ⃝

1. The trainers were considerate ⃝ ⃝ ⃝ ⃝
2. The trainers were competed and well- informed
3. I would encourage other colleagues to take this course
4. I thought the format of this training was good/appropriate

⃝ ⃝ ⃝ ⃝

⃝ ⃝ ⃝ ⃝

⃝ ⃝ ⃝ ⃝

1. My expectations for this training were met ⃝ ⃝ ⃝ ⃝

The major strengths of this training were:

The major weaknesses of this training were:

This course could be improved by:
